# The diversity of the intestinal microbiota in patients with alcohol use disorder and its relationship to alcohol consumption and cognition

**DOI:** 10.3389/fpsyt.2022.1054685

**Published:** 2022-12-22

**Authors:** Yishan Du, Lin Li, Chengcheng Gong, Ting Li, Yan Xia

**Affiliations:** Mental Health Centre, The First Affiliated Hospital of Harbin Medical University, Mental Health Institute, Harbin Medical University, Heilongjiang, Harbin, China

**Keywords:** alcohol use disorder, cognitive function, intestinal microbiota, 16S rRNA gene sequencing, alcohol consumption

## Abstract

**Introduction:**

Alcohol use disorder (AUD) has evolved into a severe social and medical issue. However, the exact environmental factors triggering AUD pathophysiology remain unknown. A growing body of research has shown that environmental elements can affect the brain *via* the microbiota-gut-brain axis.

**Methods:**

We employed 16S rRNA gene sequencing technology to investigate the composition and diversity of intestinal microbiota in 32 AUD males and 35 healthy controls (HCs), as well as its relationship on cognitive function.

**Results:**

Our findings showed that the alpha diversity indices in AUDs were much lower than HCs. The abundances of *Faecalibacterium*, *Gemmiger*, *Lachnospiracea_incertae_sedis*, *Megamonas*, and *Escherichia* were significantly different between AUD and HC groups and could be used as a basis for judging whether excessive drinking. The abundances of *Faecalibacterium*, *Gemmiger*, *Escherichia*, and *Fusobacterium* can be used to judge the cognitive function of the population.

**Conclusion:**

These data suggested that the gut dysbiosis in AUD patients, and some specific microbiota were considered to be related to alcohol intake and cognitive function. This study provides important information for further study of the pathogenesis of AUD from the perspective of intestinal microbiota.

## 1 Introduction

Alcohol consumption is the third largest cause of illness and disability in the world, accounting for 5.3% of all fatalities globally (1). It is well known that alcohol consumption is a risk factor for a variety of health problems. Currently, most research has concentrated on metabolism, neurotransmitters, neuroimaging, and the effect of alcohol use on neuronal functioning in the brain (2). However, the pathophysiology of AUD remains unknown, and there is a paucity of neurobiological markers to diagnose and forecast the risk of AUD, as well as new therapy targets and directions.

The human intestinal microbiota is a complex community of more than 100 trillion microorganisms with coding genes that are 100 times larger than the human genome (3). It is well known that the gut microbiota plays crucial physiological functions and is required for human survival (4, 5). However, the composition of gut microbiota can be affected by intrinsic and extrinsic factors, including genetic predisposition, diet, antibiotics, alcohol, circadian rhythm disruption, psychological stress, and aging (6).

A growing number of research in rodents and human have indicated that the composition of gut microbiota and the gut barrier can be effected by acute or chronic alcohol consumption and can also be linked to behavioral symptoms (7–9). Yang et al. demonstrated in an alcohol-dependent rat model that alcohol consumption affected the composition and community structure of the gut microbiota, particularly the commensal microbes such as the *Lachnospiraceae* and *Prevotellaceae* families, which account for a relatively high abundance. Alcohol-induced dysbiosis of the colonic microbiota was closely linked to leaky gut, serum metabolism, and imbalance in neurotransmitter concentrations (10). Several longitudinal population studies on alcohol withdrawal have also been published. Leclercq et al. reported that cytoderm components derived from intestinal microbiota activate inflammatory pathways in peripheral blood mononuclear cells that are associated with alcohol addiction (11). Excessive alcohol consumption promotes permeability of gut and endotoxin translocation into the peripheral circulation. Hélène et al. studied microbial translocation markers and gut permeability markers in AUD patients and discovered that abstinence enhances intestinal barrier function and liver health (12). Ames et al. clarified that the Shannon diversity index was connected with anxiety in AUD patients, and *Erysipelotrichaceae* and *Lachnospiraceae* abundances were associated with alcohol consumption in a longitudinal study (13). Prebiotic fiber supplementation during alcohol withdrawal significantly altered microbiota abundance, increased serum levels of brain-derived neurotrophic factor and improved sociability scores in AUD patients, implying that prebiotics can modulate the intestinal microbiota and social behavior in AUD patients (14).

Long-term alcohol usage contributes to alcohol-related brain damage and extensive cognitive impairment (15, 16) and those with AUD are also more likely to develop Wernicke–Korsakoff syndrome. These result in diagnostic characteristics such as rapid memory loss, learning disabilities, and amnesic confabulatory syndrome (17). Alcohol addiction results in defects in intellectual function including cognitive impairment and memory degradation, and these impairments may last long after withdrawal (18). The Health Council of the Netherlands discovered evidence that alcohol consumption can harm brain development in teenagers and young people whose brains are still developing (19). Individuals with AUD who have impaired cognitive function have more severe alcohol addiction. As a result, appropriate therapies are required to avoid cognitive decline in people with AUD.

Studies directly assessing alterations in gut microbiota in AUD patients without abstinence were scarce, especially studies on the effects of gut microbiota on cognitive function in AUD patients. In this study, we first looked at the alterations in cognitive performance of AUD patients who had never experienced alcohol withdrawal. The intestinal microbial communities of 32 AUD patients and 35 healthy controls (HCs) were then examined using 16S rRNA gene sequencing to see if changes in the gut microbiome were related to alcohol consumption. Furthermore, we investigated whether there is a link between cognitive impairment and alteration in the intestinal microbiota in AUD patients.

## 2 Materials and methods

### 2.1 Subjects

We recruited 32 male AUD patients who meet the diagnostic criteria for moderate to severe alcohol use from the outpatient clinic of the First Affiliated Hospital of Harbin Medical University. Diagnostic criteria were based on the fifth edition of the Diagnostic and Statistical Manual of Mental Disorders, and the presence of four or more symptoms in the criteria was defined as moderate to severe alcohol use. AUD patients were between 18 and 60 years of age without smoking and were not abstaining from alcohol at enrollment. Age, body mass index (BMI), and diet-matched healthy control participants who did not smoke or drink (HCs; *n* = 35) were recruited through the medical examination facility.

All subjects were free of functional digestive disorders and other drug addictions, had not used antibiotics, probiotics, or prebiotics in the 3 months prior to research participation, did not use any routine medicines, and did not adhere to any diet (e.g., gluten-free/casein-free diet).

This work was approved by the Human Research Ethics Committee of Harbin Medical University and was performed in accordance with the Declaration of Helsinki. All individuals supplied written informed consent before inclusion, and there was no remuneration for their involvement in the study.

### 2.2 Demographic and neurocognitive assessment

Every participant completed a self-report questionnaire that collected socio-demographic information such as age, BMI, education level, marital status, occupational status, and dietary information. The overall number of drinking years, the average daily drinking volume, the number of drinking days in the past month and the number of alcohol-addicted years were all investigated in this study. Participants’ cognitive ability was evaluated using the Montreal Cognitive Assessment (MoCA) and Mini-mental State Examination (MMSE). The total score of MoCA is 30, when its score is 26 or more, it is considered normal cognitive function, when its score is less than 26, it is considered impaired cognitive ability; MMSE scores 30 out of 30, the assessment score is related to the literacy level, when the illiterate score is 17 or more, elementary school literacy score is 20 or more, junior high school and above literacy level is more than 24, it is considered normal cognition. These tests had been frequently used in prior studies and were reasonably practicable (20, 21).

### 2.3 Fecal sample collection and 16S rRNA gene sequencing

Fecal samples were obtained from recruited participants, then promptly frozen and stored at −80°C before examination. All stool samples were sequenced uniformly after 3 months for 16SrRNA sequencing. High-throughput sequencing technology based on 16SrRNA gene has been widely used. By analyzing the species distribution, community characteristics and functions in the microbial community, the differential flora between different samples or groups is found, and the interaction between microorganisms and the environment is clarified. Bacterial DNA was extracted according to the manufacturer’s instructions using Qubit^®^ dsDNA BR Assay Kit (Axygen Biosciences, USA). The quality and quantity of the extracted DNA samples were verified by Agarose Gel Electrophoresis (Concentration of Agarose Gel: 1%, Voltage: 150 V, Electrophoresis Time: 40 min). Primers 5′ACTCCTACGGGAGGCAGCAG3′ and 5′GGACTACHVGGGTWTCTAAT3′ were used to amplify the V3–V4 hypervariable regions of 16S rRNA, with 460 bp average sequence length. DNA libraries were qualified by the Agilent Technologies 2100 bioanalyzer. The qualified libraries were sequenced pair-end on the Hiseq 2500 with the sequencing strategy MiSeq-PE300 (MiSeq Reagent Kit).

### 2.4 16S rRNA gene sequencing analysis

Raw reads were filtered to remove adaptors and low-quality and ambiguous bases: (1) Take the reads that can match the primers, intercept off the primers and junction contamination (cutadapt v2.6), and get the fragment of the target region; (2) The reads were truncated at any site that received an average quality score < 20 over a 30 bp sliding window. (3) The reads containing N and low complexity (10 consecutive ATCGs) were removed. Then paired-end reads were added to tags by the Fast Length Adjustment of Short reads program (FLASH, v1.2.11) to get the tags. The tags were clustered into OTUs with a cutoff value of 97% using UPARSE software (v7.0.1090) and chimera sequences were compared with the Gold database using UCHIME (v4.2.40) to detect. Then, OTU representative sequences were taxonomically classified using Ribosomal Database Project (RDP) Classifier v.2.2 with a minimum confidence threshold of 0.6, and trained on the Greengenes database v201305 by QIIME v1.8.0. The USEARCH_global was used to compare all Tags back to OTU to get the OTU abundance statistics table of each sample. Alpha and beta diversity were estimated by MOTHUR (v1.31.2) and QIIME (v1.8.0) at the OTU level, respectively. Principal Coordinate Analysis (PCoA) was performed by QIIME (v1.8.0). Partial least-squares discrimination analysis (PLS-DA) was performed by R package mixOmics. The characterization of microorganismal features differentiating the gastric microbiota was performed using the linear discriminant analysis (LDA) effect size (LEfSe) method^1^ for biomarker discovery, which emphasizes both statistical significance and biological relevance. All sequence data has been deposited in a publically accessible database, with serial number PRJNA867698.^2^

### 2.5 Statistical analysis

Age, BMI, education, and other factors such as MOCA, MMSE scores were compared between the AUD group and the HC group using independent samples *t*-test or chi-square test by SPSS 25.0. The samples were grouped according to whether drinking, MoCA score and MMSE score, and three different sample distributions were obtained. Using the wilcoxon text method, the data were screened for differential flora according to different sample distributions. Then, the logistic regression algorithm was used to verify whether the differential bacteria could be used as an indicator for evaluating sample grouping, and the verification results were displayed using the Receiver Operating Characteristic (ROC) curve. All tests of significance were two sided, and *P* < 0.05 or corrected *P* < 0.05 was considered statistically significant.

## 3 Results

### 3.1 Characteristics of participants in the study

The demographic data and alcohol consumption data were collected from 32 AUD patients and 35 demographically matched healthy controls. Table 1 showed the detailed characteristics of these recruited subjects. The pure alcohol intake was calculated as drinks per day (one drink was defined as 10 g of pure alcohol). There were no statistically significant differences between the two study groups in term of age, BMI, education, marriage, occupation, and diet.

**TABLE 1:** Demographic data and alcohol consumption of participants.

	AUD (*n* = 32)	HC (*n* = 35)	*P*
Age, year	47.16 ± 9.89	48.00 ± 11.31	0.747
BMI (kg/m^2^)	25.20 ± 3.77	24.85 ± 3.21	0.690
Level of education (years)	11.16 ± 3.00	11.20 ± 3.16	0.954
Marital status (married/single)	28/4	28/7	0.517
Occupational status (employed/unemployed)	26/6	29/6	1.000
Staple food (rice/noodles)	16/16	21/14	0.467
Meat and vegetable intake (balanced/imbalanced)	26/6	26/9	0.566
Average daily drinking volume in the past month (drinks)	6.00 (4.00, 9.75)	NA	
Drinking days in the past month (days)	23.50 (20.00, 30.00)	NA	
Years of drinking (years)	30.00 (20.50, 35.70)	NA	
Years of alcohol addiction (years)	10.00 (5.00, 26.50)	NA	

NA, not applicable.

### 3.2 Cognitive function in people with AUD and HC

The MoCA (*t* = −4.074, *P* = 0.000) and MMSE (*t* = −5.170, *P* = 0.000) test scores of AUD patients were significantly lower than those of HC, and the differences were statistically significant (*P* < 0.001) (Table 2).

**TABLE 2:** Comparison of MoCA and MMSE scores between AUD and HC.

	AUD (*n* = 32)	HC (*n* = 35)	*t*	*P*
MoCA	23.28 ± 3.19	25.89 ± 1.95	−4.074	0.000**
MMSE	26.09 ± 2.07	28.37 ± 1.52	−5.170	0.000**

**P < 0.001.

### 3.3 Correlation between cognitive function and alcohol consumption in AUD

The MoCA score was negatively correlated with the years of drinking (*r* = −0.621, *P* = 0.000) and the years of alcohol addiction (*r* = −0.357, *P* = 0.045); the MMSE score was negatively correlated with the year of drinking (*r* = −0.540, *P* = 0.001) (Table 3).

**TABLE 3:** Correlation between the scores of MoCA and MMSE and drinking in AUD.

	MoCA		MMSE	
	*r*	*P*	*r*	*P*
Average daily drinking volume in the past month (drinks)	0.033	0.856	0.040	0.829
Drinking days in the past month (day)	–0.313	0.081	–0.284	0.116
Years of drinking (years)	–0.621	0.000**	–0.540	0.001*
Years of alcohol addiction (years)	–0.357	0.045*	–0.268	0.139

*P < 0.05, **P < 0.001.

### 3.4 Comparison of intestinal microbiome between the AUD and HC groups

#### 3.4.1 Alpha diversity indices

We used Spearman method to analyze the correlation of HC group, and there was no obvious correlation. Estimates of community richness were derived from alpha diversity indexes such as Sobs, Chao, Ace, Shannon, Simpson, and Coverage. The Sobs, Chao, Ace, and Shannon indices were lower, with a larger Simpson diversity index in AUDs compared with HCs. There was a significant difference in the Ace, Simpson, and Shannon indices between the two groups (*P* < 0.05) (Table 4). Therefore, the richness and diversity of intestinal microbiota in AUDs were lower than in HCs. According to the Coverage Index, the sequencing results can accurately describe the distribution of microorganisms in the sample.

**TABLE 4:** Comparison of the alpha diversity index in the two groups.

	AUD (*n* = 32)	HC (*n* = 35)	*P*
Sobs	158.66 ± 38.74	182.63 ± 54.86	0.070
Chao	190.59 ± 42.69	218.62 ± 62.39	0.062
Ace	191.06 ± 37.98	219.52 ± 57.84	0.031*
Shannon	2.31 ± 0.50	2.79 ± 0.46	0.000**
Simpson	0.23 ± 0.11	0.16 ± 0.09	0.001*
Coverage	1.00 ± 0.00	1.00 ± 0.00	0.171

*P < 0.05, **P < 0.001.

#### 3.4.2 Beta diversity indices

**FIGURE 1:**
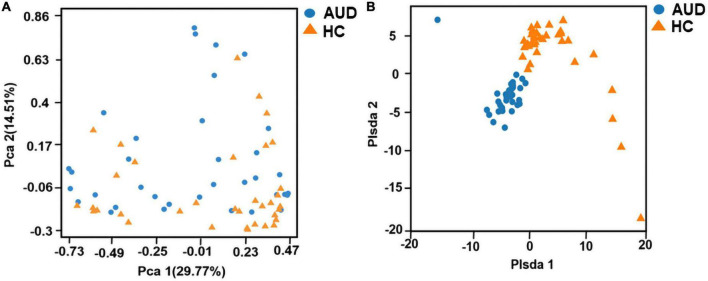
Beta diversity indices based on weighted UniFrac. Principal coordinates analysis (PCA) based on weighted UniFrac **(A)** and partial least squares-discriminant analysis (PLS-DA) **(B)** generated from fecal samples of patients with AUD and healthy controls (HC).

Principal component analysis (PCA) and Partial least-squares discrimination analysis (PLS-DA) based on the weighted-unifrac distance algorithm were performed on the two groups of samples to test the similarity and difference of community composition between the two groups of samples. Adonis test was used for the differences between groups. The results showed that there was a statistically significant difference in community composition between the two groups (*P* < 0.0001) (Figures 1A, B).

#### 3.4.3 Relative abundance analysis of fecal bacterial

**FIGURE 2:**
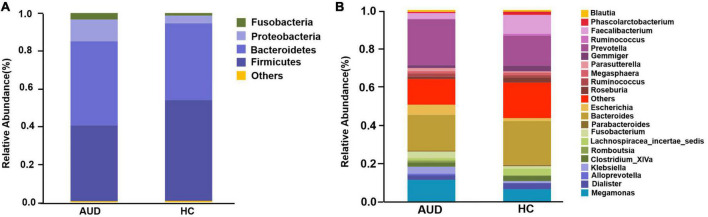
Comparison of the relative abundance of gut microbiota at different levels between AUD and HC groups. **(A)** The phylum level and **(B)** the genus level.

Based on AUDs and HCs, we investigated at the abundance and distribution of intestinal microbiota. At the phylum level, those discriminative OTUs were mostly allocated to the phyla *Firmicutes* (40.05 vs. 53.20%), *Bacteroidetes* (44.45 vs. 40.67%), *Proteobacteria* (11.49 vs. 4.04%), and *Fusobacteria* (3.42 vs. 1.34%) (Figure 2A). The relative abundance of *Firmicutes* was significantly lower in AUD subjects compared to HCs (*P* < 0.05), and the abundance of *Cyanobacteria* increased in AUDs compared to HCs (*P* < 0.05). *Bacteroides* (19.57 vs. 23.34%) and *Prevotella* (22.84 vs. 15.22%) were the dominant genus in both groups. The abundance of *Bacteroides, Faecalibacterium, Dialister, Clostridium_ XlVa, Gemmiger, Lachnospiracea_incertae_sedis* was lower in AUDs compared to HCs, and the changes in the abundance of *Faecalibacterium, Gemmiger*, and *Lachnospiracea_incertae_sedis* were statistically significant (*P* < 0.05). The abundance of *Prevotella, Megamonas, Escherichia*, and *Fusobacterium* was higher in the AUD group compared to HC, and changes in the abundance of *Megamonas* and *Escherichia* were statistically significant (*P* < 0.05) (Figure 2B).

#### 3.4.4 Composition of changed fecal microbiota in AUD patients

**FIGURE 3:**
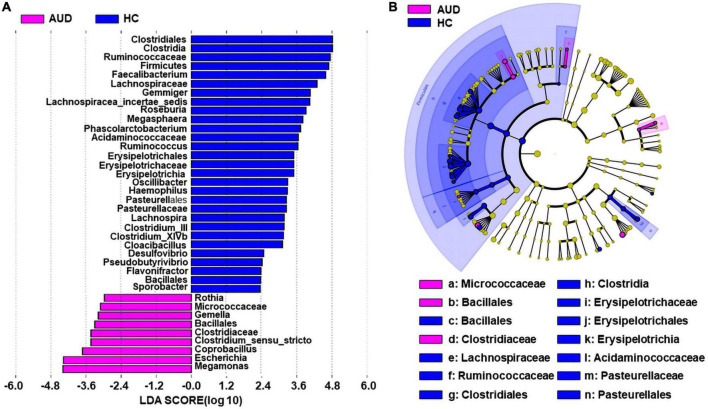
Differential bacterial taxa between AUD patients and HCs. The LEfSe identified the taxa with the greatest differences in abundance between AUD patients and the HCs. Only the taxa meeting a significant LDA threshold value of > 2 are shown **(A)**. Cladogram showing phylogenetic relationships between taxa that are statistically different **(B)**.

We applied linear discriminant analysis of effect size (LEfSe) to identify differentially expressed taxa. First, we generated a bar plot of the effect size of taxa with differential relative abundance, and our discriminant analyses showed that many key taxa were clearly different between the AUD and the control group (LDA score > 2, *p* < 0.05, Figure 3A). Of note, we found that *Firmicutes* had significantly decreased at the phylum level in AUD. Compared with HCs, at the genus level, *Megamonas*, *Escherichia*, *Coprobacillus*, *Clostridium*, *Gemella*, and *Rothia* had increased in AUD patients. Secondly, we generated a cladogram to illustrate the relationship between different taxa (Figure 3B). This figure also showed that *Micrococcaceae*, *Bacillales*, *Clostridiaceae* a higher relative abundance of AUD patients.

### 3.5 Differential bacterial screening for different sample distributions

**FIGURE 4:**
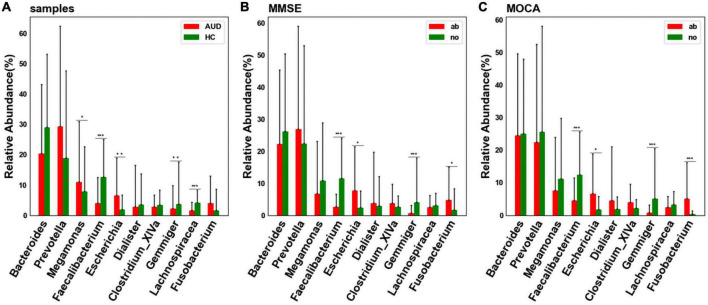
Differential bacterial screening for different sample distributions. **(A)** Comparison of differences in major bacteria at the genus levels between AUD and HC groups; **(B)** screening of differential bacteria by MMSE Score; **(C)** screening of differential bacteria by MoCA Score [*indicates statistical differences (*P* < 0.05), **(*P* < 0.01), *** (*P* < 0.001)].

At the genus level, considering the influence of the number of single bacteria, the median test method was used to test and analyze the top ten bacteria. We detected differences in bacterial abundance between the AUD and HC groups (Figure 4A). Compared with the control group, the abundance of *Faecalibacterium* (*P* < 0.005), *Gemmiger* (*P* < 0.01), and *Lachnospiracea_incertae_sedis* (*P* < 0.005) decreased in the AUD group; the abundance of *Megamonas* (*P* < 0.05) and *Escherichia* (*P* < 0.01) increased.

Grouped according to cognitive function, we found that the abundance of *Faecalibacterium* and *Gemmiger* decreased (*P* < 0.005) in cognitively impaired subjects (Figures 4B, C). Interestingly, we found that the abundance of *Faecalibacterium*, *Gemmiger* and *Escherichia* also varied significantly in AUD patients.

### 3.6 ROC curve for disease differentiation

**FIGURE 5:**
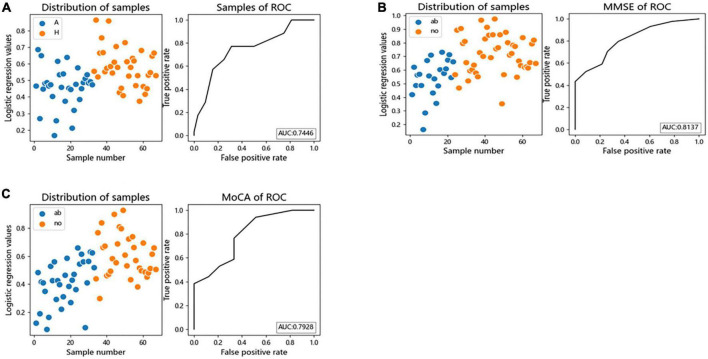
The differential genera as AUD and cognitive function diagnostic markers. **(A)** Receiver operating characteristic (ROC) curves of subject genus-level samples used to differentiate AUD patients from HC; **(B)** ROC curves classified by the MMSE scores of subject were used to predict cognitive function; **(C)** ROC curves classified by the MoCA scores of subject were used to predict cognitive function. AUC, the area under the receiver operating characteristic curve; ab, abnormal cognitive function; no, normal cognitive function.

We performed logistic regression and Receiver Operating Characteristic (ROC) curves for differential bacteria at the genus level to assess the value of differential bacteria as biomarkers, finding that the area under the receiver operating characteristic curve (AUC) was greater than 70% (AUC = 0.7446), indicating that the abundances of *Faecalibacterium*, *Gemmiger*, *Lachnospiracea_incertae_sedis*, *Megamonas*, and *Escherichia* the could be used as indicators for predicting alcohol consumption (Figure 5A).

Logistic regression and ROC curve were performed on the differential bacteria obtained by MoCA and MMSE scores, and it was found that the AUC of the two models were both greater than 70% (MOCA:AUC = 0.7928, MMSE:AUC = 0.8137), indicating that the abundances of *Faecalibacterium*, *Gemmiger*, *Escherichia*, and *Fusobacterium* could be used as indicators to predict cognitive impairment (Figures 5B, C).

## 4 Discussion

In the present study, by sequencing the hypervariable V3–V4 region of the 16S rRNA gene, we were able to investigate bacterial diversity and community structure in 67 stool samples. We demonstrated that the gut microbiota was dysregulated in AUD patients compared to HC. Alpha diversity decreased and PLS-DA analysis of beta diversity was able to discriminate the AUD group from the HC group, and the beta diversity of AUD group increased. At the level of phylum and genus, the distribution of bacterial groups in AUD patients was significantly different from that in HC, and the differential bacteria could be used as a basis for judging whether the subjects had excessive drinking.

Previous studies have shown that the abundance of *Bacteroidetes* and *Fusobacteria* increased with malignant colorectal cancer (22). Tonatiuh et al. found that the gut microbiota in patients with alcoholic cirrhosis showed a significant increase in the pathogenic/pathogenic genera *Escherichia/Shigella* and *Prevotella*, and a decrease in beneficial bacteria such as *Blauella*, *Faecalibacterium* and alpha diversity decreased (23). The correlation of alcohol consumption with the abundance of *Fusobacterium* OTUs in cancer tissue suggested a possible link between alcohol metabolism and subsequent tumorigenesis caused by *Fusobacterium* (24). We found that the abundance of *Bacteroidetes* and *Fusobacteria* increased in AUD patients, suggesting that alcohol consumption may have an effect on colorectal disease. There are increases in the *Proteobacteria* phylum and the class *Gammaproteobacteria* in alcoholics (20, 21); many members of this class are enteric pathogens. *Proteobacteria*, including *Escherichia*, have been linked to inflammation (25). The abundance of *Proteobacteria* and *Escherichia* were higher in AUD patients, suggesting that the inflammatory microbiota of AUD patients may be increased. Another study discovered that *Escherichia* spp. could create endogenous ethanol, and its abundance correlated with the amount of ethanol in peripheral blood (26). *Escherichia coli* has been shown to induce inflammation and anxiety-like phenotypes in mice through an NF-kB-dependent pathway and hippocampal involvement (27). *Klebsiella* oxytocin colonization in mice led to a similar outcome with increased anxiety-like phenotype following increased inflammation through NF-kB-dependent pathways (28). *Faecalibacterium* is widely thought to defend against gastrointestinal and extraintestinal diseases (29), and also appears to be beneficial to overall health, minimizing the occurrence of metabolic disorders as well as a variety of chronic diseases and inflammatory pathologies (30). Our study showed that the abundance of *Faecalibacterium* was reduced in AUD patients, indicating that alcohol consumption can destroy beneficial bacteria in the gut, rendering the microbiota detrimental to the human body and weakening their protective functions. *Megamonas* and other microorganisms are involved in nutrient metabolism, however, excessive metabolism may cause changes in the intestinal microenvironment. This can include changes in the intestinal acid-base balance. Therefore, it is unknown if the rise in this microorganism is beneficial or harmful to the human body.

Prior research studies have found that drinking altered the microbial composition of the gut, although the findings were inconsistent. A study involving 24 AUD patients reported that, when compared to the control group, the intestinal microbiota of AUD patients changed. This was primarily manifested in an increase in the abundance of *Proteobacteria* and a reduction in the abundance of bacteria from genus *Faecalibacterium*, which is consistent with our findings. In the same study, the AUD group also exhibited a higher abundance of bacteria from the genera *Sutterella*, *Holdemania*, and *Clostridium* (31). In contrast, we found that the abundance of *Clostridium* was lower in AUD subjects compared to controls. Another study discovered that the alpha diversity of gut microbiota and of the microorganism *Akkermansia* was lower in AUD patients, while the abundance of the genus *Bacteroides* was higher, compared with HC. The identification accuracy of *Akkermansia* and *Bacteroides* to AUD patients was 93.4% (21). A meta-analysis revealed that the gut microbial changes in AUD patients were relatively consistent, with the abundance of *Akkermansia muciniphila* and *Faecalibacterium prausnitzii* in the intestine decreasing significantly and the abundance of *Enterobacteriaceae* increasing significantly. The analysis of changes in *Bifidobacteria* abundance was inconsistent. At the phylum level, the number of *Proteobacteria* increased significantly, while *Bacteroidetes* decreased significantly (32). Research has revealed that alcohol consumption can produce changes in gut microbial composition in animal models, with most of the data focusing on changes in the abundance of *Firmicutes* or *Bacteroidete*s, although the results have been inconsistent (33–35). The researchers found that gut microbiota depletion by antibiotics administration causes a reduction in alcohol consumption and altered the relative abundance of bacteria such as *Firmicutes*, *Bacteroidetes* and *Cyanobacteria* (36). Leclercq et al. showed that AUD patients have changes in serum metabolites from the kynurenine/tryptophan pathway compared to control individuals, the ratio of KYNA/QUIN was positively correlated with the fecal abundance of *Faecalibacterium*. Plasma tryptophan and KYNA levels were also inversely associated with depression and alcohol craving, respectively (37).

The discrepancies between these studies could be attributed to differences in sample sizes, demographic, and clinical characteristics of the AUD participants, and/or various statistical approaches for detecting AUD-related intestinal microbiota changes. However, these studies all consistently show that alcohol consumption was associated with significant changes in the composition of intestinal microbiota. The variation in the intestinal microbial communities seen between AUD individuals and HCs were found to be independent of confounding factors such as age and BMI, basically consistent with previous research (38). Individuals in Heilongjiang have extremely similar lifestyles and dietary habits (that is, rice intake is generally dominant, along with small amounts of local meats and vegetables). There are no significant differences in the staple meat-vegetable balance between the two groups. As a result, the dietary element is unlikely to have a major impact on our current findings. To summarize, we discovered that AUD was related with distinctive abnormalities in the gut microbiota by enrolling well-matched patients, implying that the gut microbiota may play an essential role in the progression etiology of AUD.

We found that the abundances of *Faecalibacterium*, *Gemmiger*, *Escherichia*, and *Fusobacterium* can be used as indicators to predict cognitive impairment. Interestingly, the abundances of *Faecalibacterium*, *Gemmiger*, and *Escherichia* were associated with alcohol consumption and cognitive function. Throughout the development of alcoholism, individuals were accompanied by numerous cognitive changes and dysfunctions, characterized by changes in behaviors such as emotional processing, memory, and executive function (39). Carlson et al. first showed that microbial composition and abundance were tightly correlated with cognitive function (40), and that changes in intestinal microbiota affected exploratory and communicative behaviors, as well as cognitive ability. An increasing amount of research suggested that the intestinal microbiome was essential for emotional and social cognition, which provided us additional justification to investigate the relationship between microbiome and alcohol addiction (41). Changes in the intestinal mucosa and the community of microorganisms of bacteria that colonize the gut (42) may deepen addiction *via* metabolic and inflammatory pathways, resulting in heightened emotional and social cognitive deficits. While there have been few studies examining the effects the microbiome has on the cognition in alcoholism, there have been many studies explaining the impact the microbiome has on alcoholic liver disease, which is known involvement in neurological diseases and conditions such as hepatic encephalopathy and neuroinflammation (43).

Consistent with the findings of our study, Ling et al. reported that the abundant butyrate-producing genera such as *Faecalibacterium* and *Gemmiger* decreased significantly, which was positively correlated with clinical indicators such as cognitive function scores, and were negatively correlated with inflammatory cytokines, such as TNF-α and chemokines in Alzheimer’s Disease patients (44). Liu et al. found that *Gemmiger* was positively related with the MoCA scores in patients with post-stroke cognitive impairment (45). Overgrowth of endogenous or exogenous *Escherichia* leads to altered gut microbiota and disrupts gut immune homeostasis, leading to gut inflammation, depression, and cognitive impairment by inducing IL-1β and corticosterone production (28, 46). Jiao et al. reported that the abundance of *Faecalibacterium* was significantly reduced in the drinking case group and its protective effect on inflammatory liver injury (47).

*Faecalibacterium*, a major member of the Firmicutes phylum, was considered one of the most important bacterial indicators of a healthy gut, regulating inflammation at the level of the gut epithelium (48). It has been found that in many cases of intestinal diseases, beneficial fecal bacilli were reduced. Biagi et al. and Wang et al. showed that such butyrate producers as *Faecalibacterium, Roseburia, and Coprococcus* were negatively correlated with age (49, 50). Tongeren et al. observed a decreased relative abundance of *Faecalibacterium* in frail and elderly patients (51). In line with these findings, the decreased proportion of *Faecalibacterium* and increased *Bifidobacterium* have been found in elderly patients with Parkinson’s disease (52, 53). Previous studies have found that *Faecalibacterium* has anti-inflammatory properties due to its capability to produce butyrate and induce a tolerogenic cytokine profile. Decreased abundance of *Faecalibacterium* may contribute to a pro-inflammatory gut environment. Liu et al. found that high-altitude Tibetan fermented milk could increase microbial diversity and elevate the levels of *Bacteroides* and *Faecalibacterium* in AD mice model, which were associated with cognitive improvements in mice afflicted with AD (54). The clinical comparative analyses and studies on animal mechanics confirmed the beneficial roles of *Faecalibacterium* on mental health. *Faecalibacterium* has been found consistently associated with higher quality-of-life (QoL) indicators (55).

Some constraints should be mentioned here. First, because all patients were Han Chinese, it is impossible to rule out site-specific and ethnic biases in microbial phenotypes. Additional investigations including ethnically diverse patients from different clinical settings are needed to corroborate the current data. Second, because this is a cross-sectional study, the causal relationship between changes in intestinal flora and drinking and cognitive function cannot be determined. Future research will focus on expanding pre-and post-control surveys of abstinence patients to assess changes in gut microbiota after abstinence. Third, the current study only confirmed phenomenological gut microbiota changes in AUD patients and did not investigate intermediate pathway changes of the “microbiota-gut-brain axis” and the precise pathophysiological mechanism of gut microbial impact on cognitive function. Fecal microbiota transplantation (FMT) may be used in the future to confirm the causal relationship between intestinal microbiota composition and AUD symptoms.

In conclusion, we found that the bacterial composition of the fecal microbiota was altered and bacterial diversity was reduced in patients with AUD compared with healthy individuals. The structural dysbiosis of the fecal microbiota in AUD patients is characterized by a decrease in the abundance of beneficial bacteria such as *Faecalibacterium* and *Gemmiger*, and an increase in the abundance of harmful bacteria such as *Escherichia* and *Fusobacterium*, and these bacteria serve as biological markers for distinguishing between alcohol consumption and cognitive impairment. The gut microbiota may play an important role in the pathogenesis of AUD and cognitive impairment caused by alcohol consumption, and provide a reference for the development of microbiota-targeted therapeutic drugs in the future.

## Data availability statement

The original contributions presented in this study are publicly available. This data can be found here: https://www.ncbi.nlm.nih.gov/bioproject/PRJNA867698/.

## Ethics statement

The studies involving human participants were reviewed and approved by the Human Research Ethics Committee of Harbin Medical University. The patients/participants provided their written informed consent to participate in this study. Written informed consent was obtained from the individual(s) for the publication of any potentially identifiable images or data included in this article.

## Author contributions

YD and YX conceived and designed the research, wrote the initial manuscript, and revised the manuscript. YD, TL, and CG collected the data and conducted the research. YD and LL analyzed and interpreted the data. YX had primary responsibility for final content. All authors read and approved the final manuscript.

## Footnotes

1
https://huttenhower.sph.harvard.edu/galaxy/

2
https://www.ncbi.nlm.nih.gov/sra/PRJNA867698
