# Supplementary material for: The diversity of the intestinal microbiota in patients with alcohol use disorder and its relationship to alcohol consumption and cognition
Source: Front Psychiatry. 2022 Dec 22;13:1054685. doi: 10.3389/fpsyt.2022.1054685 (PMC9814012; doi:10.3389/fpsyt.2022.1054685)
Supplement: Supplementary file 1 [file Table_1.DOCX]

**SUPPLEMENTARY TABLES**

**Table S1. Detailed Characteristics of Recruited Subjects**

**Table S2. Demographic and Clinical Details of Recruited Subjects**

**Table S3. 16S rRNA Sequencing Data from Recruited Subjects**

**Table S4. Assessment of (α) Phylogenetic Diversity between AUD Patients and Healthy Controls**

**Table S5. Discriminatory OTUs between AUD Patients and Healthy Controls**

**Table S1. Detailed Characteristics of Recruited Subjects**

| **Group** | **Age (years)** | **BMI** | **Level of education (years)** | **Marital status** | **Occupational status** | **Staple food** | **Meat and vegetable intake** | **Average daily drinking volume in the past month (drinks)** | **Drinking days in the past month** | **Years of drinking** | **Years of alcohol addiction** | **MoCA Scores** | **MMSE Scores** |
| --- | --- | --- | --- | --- | --- | --- | --- | --- | --- | --- | --- | --- | --- |
| AUD | 31 | 30.5 | 19 | single | employment | noodles | imbalanced | 4 | 10 | 10 | 1 | 30 | 29 |
| AUD | 32 | 30.2 | 19 | married | employment | noodles | balanced | 15 | 26 | 11 | 5 | 30 | 30 |
| AUD | 36 | 30.1 | 12 | married | unemployment | rice | balanced | 9 | 26 | 13 | 0 | 29 | 30 |
| AUD | 32 | 25.9 | 5 | married | unemployment | rice | imbalanced | 1.5 | 10 | 20 | 1 | 27 | 29 |
| AUD | 52 | 24.4 | 16 | married | employment | rice | balanced | 5 | 25 | 35 | 0 | 23 | 25 |
| AUD | 40 | 26.2 | 19 | married | employment | rice | balanced | 1.5 | 20 | 5 | 20 | 25 | 26 |
| AUD | 57 | 29.0 | 12 | married | unemployment | rice | balanced | 20 | 30 | 40 | 20 | 23 | 26 |
| AUD | 61 | 19.4 | 16 | married | employment | noodles | balanced | 20 | 30 | 43 | 5 | 25 | 26 |
| AUD | 35 | 24.2 | 16 | married | employment | noodles | imbalanced | 6 | 30 | 23 | 10 | 23 | 25 |
| AUD | 49 | 18.8 | 19 | married | employment | rice | balanced | 6 | 20 | 31 | 30 | 24 | 26 |
| AUD | 40 | 25.7 | 19 | married | employment | noodles | balanced | 3 | 30 | 26 | 25 | 21 | 26 |
| AUD | 56 | 24.5 | 12 | married | unemployment | noodles | balanced | 10 | 25 | 36 | 6 | 19 | 24 |
| AUD | 49 | 26.9 | 12 | single | employment | noodles | balanced | 6 | 20 | 30 | 20 | 29 | 30 |
| AUD | 54 | 19.9 | 12 | married | employment | noodles | imbalanced | 3 | 30 | 34 | 6 | 20 | 24 |
| AUD | 52 | 19.0 | 12 | married | employment | rice | balanced | 3 | 12 | 22 | 10 | 20 | 24 |
| AUD | 55 | 26 | 16 | married | employment | noodles | balanced | 14 | 30 | 27 | 27 | 22 | 25 |
| AUD | 59 | 29.8 | 9 | single | employment | noodles | balanced | 5 | 30 | 40 | 40 | 19 | 22 |
| AUD | 42 | 24.6 | 19 | married | employment | rice | balanced | 6 | 12 | 35 | 35 | 26 | 28 |
| AUD | 52 | 27.7 | 16 | married | employment | rice | balanced | 5 | 12 | 30 | 30 | 23 | 27 |
| AUD | 59 | 23.8 | 16 | married | employment | noodles | balanced | 6 | 30 | 32 | 20 | 20 | 26 |
| AUD | 55 | 24.4 | 16 | married | employment | noodles | imbalanced | 6 | 30 | 40 | 40 | 23 | 26 |
| AUD | 58 | 22.8 | 16 | married | employment | noodles | balanced | 6 | 28 | 40 | 40 | 20 | 25 |
| AUD | 55 | 24.1 | 12 | married | employment | noodles | balanced | 4 | 25 | 40 | 15 | 19 | 24 |
| AUD | 58 | 22.6 | 16 | married | employment | rice | balanced | 10.5 | 30 | 40 | 35 | 22 | 26 |
| AUD | 52 | 26.8 | 16 | single | employment | rice | balanced | 3 | 28 | 32 | 0 | 25 | 27 |
| AUD | 29 | 17.3 | 16 | married | employment | rice | imbalanced | 9 | 25 | 9 | 2 | 27 | 28 |
| AUD | 34 | 31.7 | 12 | married | employment | rice | balanced | 6 | 20 | 20 | 3 | 26 | 28 |
| AUD | 50 | 21 | 12 | married | employment | rice | balanced | 6 | 30 | 29 | 10 | 26 | 27 |
| AUD | 50 | 27 | 9 | married | unemployment | rice | balanced | 8 | 20 | 30 | 20 | 20 | 22 |
| AUD | 34 | 28.4 | 16 | married | employment | rice | balanced | 10 | 20 | 15 | 10 | 25 | 27 |
| AUD | 46 | 29.4 | 12 | married | employment | noodles | balanced | 14 | 10 | 26 | 10 | 23 | 26 |
| AUD | 45 | 24.2 | 12 | married | unemployment | noodles | balanced | 1.5 | 20 | 25 | 10 | 25 | 28 |
| HC | 26 | 27.1 | 19 | single | employment | rice | balanced | – | – | – | – | 30 | 30 |
| HC | 28 | 20.5 | 19 | single | employment | rice | balanced | – | – | – | – | 27 | 30 |
| HC | 55 | 24.8 | 9 | married | employment | noodles | balanced | – | – | – | – | 26 | 28 |
| HC | 53 | 22.5 | 9 | married | employment | noodles | imbalanced | – | – | – | – | 26 | 28 |
| HC | 52 | 24.2 | 12 | married | employment | noodles | imbalanced | – | – | – | – | 26 | 28 |
| HC | 57 | 20.0 | 9 | married | employment | rice | imbalanced | – | – | – | – | 24 | 29 |
| HC | 52 | 25.7 | 9 | married | employment | noodles | balanced | – | – | – | – | 24 | 32 |
| HC | 27 | 21.6 | 19 | single | employment | rice | balanced | – | – | – | – | 29 | 30 |
| HC | 26 | 19.4 | 16 | single | employment | noodles | balanced | – | – | – | – | 29 | 30 |
| HC | 56 | 28.4 | 16 | married | employment | rice | balanced | – | – | – | – | 27 | 28 |
| HC | 59 | 27.9 | 9 | married | employment | rice | imbalanced | – | – | – | – | 26 | 26 |
| HC | 50 | 24.3 | 12 | married | employment | rice | balanced | – | – | – | – | 25 | 27 |
| HC | 56 | 25.9 | 9 | married | employment | rice | balanced | – | – | – | – | 26 | 28 |
| HC | 36 | 21.8 | 12 | married | unemployment | rice | balanced | – | – | – | – | 22 | 26 |
| HC | 46 | 27.7 | 9 | married | employment | rice | balanced | – | – | – | – | 24 | 30 |
| HC | 45 | 27.9 | 5 | married | unemployment | noodles | balanced | – | – | – | – | 24 | 25 |
| HC | 37 | 27.4 | 16 | married | employment | rice | balanced | – | – | – | – | 27 | 29 |
| HC | 60 | 25.0 | 12 | married | unemployment | noodles | balanced | – | – | – | – | 26 | 28 |
| HC | 49 | 28.7 | 9 | married | employment | rice | balanced | – | – | – | – | 26 | 28 |
| HC | 56 | 24.9 | 9 | married | employment | noodles | imbalanced | – | – | – | – | 24 | 27 |
| HC | 54 | 28.7 | 9 | married | employment | rice | balanced | – | – | – | – | 26 | 28 |
| HC | 55 | 27.7 | 9 | single | employment | noodles | balanced | – | – | – | – | 20 | 27 |
| HC | 54 | 18.7 | 12 | married | employment | noodles | balanced | – | – | – | – | 24 | 30 |
| HC | 53 | 26.9 | 9 | single | employment | noodles | balanced | – | – | – | – | 26 | 28 |
| HC | 56 | 24.0 | 9 | married | employment | rice | imbalanced | – | – | – | – | 26 | 28 |
| HC | 58 | 24.3 | 16 | married | employment | rice | imbalanced | – | – | – | – | 27 | 30 |
| HC | 28 | 32.1 | 16 | single | employment | rice | balanced | – | – | – | – | 27 | 30 |
| HC | 55 | 26.7 | 12 | married | employment | rice | balanced | – | – | – | – | 26 | 28 |
| HC | 29 | 22.9 | 16 | married | employment | rice | balanced | – | – | – | – | 28 | 30 |
| HC | 35 | 27.8 | 19 | married | unemployment | noodles | balanced | – | – | – | – | 28 | 30 |
| HC | 58 | 21.5 | 5 | married | unemployment | rice | balanced | – | – | – | – | 24 | 26 |
| HC | 50 | 21.5 | 5 | single | employment | rice | balanced | – | – | – | – | 26 | 27 |
| HC | 58 | 19.8 | 16 | married | employment | noodles | imbalanced | – | – | – | – | 26 | 28 |
| HC | 57 | 25.2 | 12 | married | employment | noodles | balanced | – | – | – | – | 27 | 28 |
| HC | 54 | 26.4 | 9 | married | unemployment | rice | imbalanced | – | – | – | – | 27 | 28 |

**Table S2. Demographic and Clinical Details of Recruited Subjects^a^**

|  | **AUD** | **HC** | **P *^b^*** |
| --- | --- | --- | --- |
| Sample Size | 32 | 35 | – |
| Age (years) *^c^* | 47.16±9.89 | 48.00±11.31 | 0.747 |
| BMI *^c^* | 25.20±3.77 | 24.85±3.21 | 0.690 |
| Level of education (years) *^c^* | 11.16±3.00 | 11.20±3.16 | 0.954 |
| Marital status (married /single) | 28/4 | 28/7 | 0.517 |
| Occupational status (employed/unemployed) | 26/6 | 29/6 | 1.000 |
| Staple food (rice/noodles) | 16/16 | 21/14 | 0.467 |
| Meat and vegetable intake (balanced/imbalanced) | 26/6 | 26/9 | 0.566 |
| Average daily drinking volume in the past month (drinks) *^d^* | 6.00 (4.00, 9.75) | – |  |
| Drinking days in the past month (days) *^d^* | 23.50 (20.00, 30.00) | – |  |
| Years of drinking (years) *^d^* | 30.00 (20.50, 35.7) | – |  |
| Years of alcohol addiction (years) *^d^* | 10.00 (5.00, 26.50) | – |  |
| MoCA Scores *^c^* | 23.28±3.19 | 25.89±1.95 | 0.000 |
| MMSE Scores *^c^* | 26.09±2.07 | 28.37±1.52 | 0.000 |
| *^a^*  Abbreviations: AUD, alcohol use disorder; HC, Healthy controls; BMI, Body Mass Index; MoCA, Montreal Cognitive Assessmentry; MMSE, Mini-mental State Examination | | | |
| *^b^* Two-tailed Student’s t-test for continuous variables (age, BMI, level of education, MoCA and MMSE). Chi-square analyses for categorical variables (marital status, occupational status, staple food, eat and vegetable intake). | | | |
| *^c^* Values expressed as the means ± standard deviations. | | | |
| *^d^* Values expressed as median (smaller quartile, larger quartile). | | | |

**Table S3. 16S rRNA Sequencing Data from Recruited Subjects**

| **Group** | **Raw reads number** | **Clean reads number** | **Read utilization ratio (%)** |
| --- | --- | --- | --- |
| AUD | 140,980.32 | 136,764 | 97.01 |
| AUD | 142,659.49 | 138,100 | 96.80 |
| AUD | 139,875.46 | 135,764 | 97.06 |
| AUD | 142,196.36 | 137,908 | 96.98 |
| AUD | 140,171.51 | 135,944 | 96.98 |
| AUD | 139,364.03 | 135,384 | 97.14 |
| AUD | 140,747.04 | 136,636 | 97.08 |
| AUD | 141,554.02 | 137,360 | 97.04 |
| AUD | 140,998.60 | 136,998 | 97.16 |
| AUD | 141,131.02 | 136,852 | 96.97 |
| AUD | 139,881.10 | 135,918 | 97.17 |
| AUD | 141,945.45 | 137,622 | 96.95 |
| AUD | 140,092.99 | 136,994 | 97.79 |
| AUD | 140,443.44 | 136,792 | 97.40 |
| AUD | 141,945.87 | 137,012 | 96.52 |
| AUD | 140,850.57 | 136,274 | 96.75 |
| AUD | 139,799.91 | 135,392 | 96.85 |
| AUD | 138,784.28 | 135,376 | 97.54 |
| AUD | 141,645.42 | 137,162 | 96.83 |
| AUD | 141,369.20 | 136,812 | 96.78 |
| AUD | 140,303.26 | 135,728 | 96.74 |
| AUD | 140,873.69 | 137,394 | 97.53 |
| AUD | 141,060.41 | 136,282 | 96.61 |
| AUD | 140,463.33 | 136,408 | 97.11 |
| AUD | 139,592.96 | 135,712 | 97.22 |
| AUD | 140,260.17 | 136,430 | 97.27 |
| AUD | 138,767.47 | 134,998 | 97.28 |
| AUD | 140,898.37 | 136,614 | 96.96 |
| AUD | 141,013.20 | 136,392 | 96.72 |
| AUD | 141,397.98 | 137,072 | 96.94 |
| AUD | 140,859.35 | 137,504 | 97.62 |
| AUD | 140,906.08 | 136,298 | 96.73 |
| HC | 139,958.50 | 136,328 | 97.41 |
| HC | 141,178.29 | 136,926 | 96.99 |
| HC | 138,603.45 | 134,962 | 97.37 |
| HC | 141,585.43 | 136,976 | 96.74 |
| HC | 141,363.24 | 137,190 | 97.05 |
| HC | 140,823.69 | 136,628 | 97.02 |
| HC | 139,679.70 | 135,886 | 97.28 |
| HC | 140,783.63 | 136,452 | 96.92 |
| HC | 139,827.72 | 135,800 | 97.12 |
| HC | 139,432.55 | 135,492 | 97.17 |
| HC | 139,822.41 | 136,336 | 97.51 |
| HC | 140,111.42 | 135,660 | 96.82 |
| HC | 140,077.13 | 136,052 | 97.13 |
| HC | 139,467.66 | 135,236 | 96.97 |
| HC | 140,810.89 | 136,414 | 96.88 |
| HC | 138,563.81 | 134,962 | 97.40 |
| HC | 140,189.72 | 136,090 | 97.08 |
| HC | 138,863.99 | 135,178 | 97.35 |
| HC | 139,533.29 | 135,354 | 97.00 |
| HC | 140,921.10 | 136,704 | 97.01 |
| HC | 139,119.56 | 135,232 | 97.21 |
| HC | 141,966.25 | 137,526 | 96.87 |
| HC | 140,775.68 | 136,786 | 97.17 |
| HC | 140,658.03 | 136,542 | 97.07 |
| HC | 140,067.40 | 135,330 | 96.62 |
| HC | 140,425.84 | 136,706 | 97.35 |
| HC | 141,161.17 | 137,226 | 97.21 |
| HC | 141,715.86 | 136,956 | 96.64 |
| HC | 140,267.75 | 136,302 | 97.17 |
| HC | 140,426.46 | 136,132 | 96.94 |
| HC | 140,105.14 | 135,560 | 96.76 |
| HC | 141,146.78 | 136,934 | 97.02 |
| HC | 140,260.63 | 136,214 | 97.11 |
| HC | 142,471.48 | 137,978 | 96.85 |
| HC | 139,707.52 | 135,390 | 96.91 |
| ***Sum*** | ***9,835,400.32*** | ***9,545,386*** | – |
| ***Average*** | ***140,505.72*** | ***136,362.66*** | ***97.05*** |
| ***SD*** | ***914.86*** | ***773*** | ***0.26*** |
| Abbreviations: AUD, alcohol use disorder; HC, healthy controls | | | |

**Table S4. Assessment of (α) Phylogenetic Diversity between AUD Patients and Healthy Controls**

| **Group** | **sobs** | **chao** | **ace** | **shannon** | **simpson** | **coverage** |
| --- | --- | --- | --- | --- | --- | --- |
| AUD | 111.0 | 132.0 | 128.626576 | 1.96656 | 0.28983 | 0.999637 |
| AUD | 186.0 | 227.052632 | 221.590381 | 2.667154 | 0.132576 | 0.999118 |
| AUD | 155.0 | 190.2 | 185.15729 | 1.951814 | 0.302232 | 0.999337 |
| AUD | 160.0 | 168.0 | 172.318681 | 3.367062 | 0.068617 | 0.999642 |
| AUD | 125.0 | 146.230769 | 144.638037 | 1.967739 | 0.222487 | 0.999541 |
| AUD | 188.0 | 219.0 | 212.254904 | 2.793762 | 0.13232 | 0.999271 |
| AUD | 258.0 | 291.681818 | 284.113137 | 3.082883 | 0.113435 | 0.999082 |
| AUD | 147.0 | 176.176471 | 177.079339 | 2.225078 | 0.220814 | 0.999226 |
| AUD | 176.0 | 207.5 | 206.259809 | 1.755552 | 0.397168 | 0.999324 |
| AUD | 75.0 | 102.6 | 137.437323 | 1.496807 | 0.361525 | 0.999599 |
| AUD | 139.0 | 175.909091 | 168.078955 | 2.258145 | 0.257571 | 0.99941 |
| AUD | 119.0 | 159.6 | 148.503018 | 2.430169 | 0.16444 | 0.999384 |
| AUD | 194.0 | 237.05 | 234.517411 | 2.553496 | 0.158302 | 0.998988 |
| AUD | 205.0 | 275.3 | 239.397847 | 2.155616 | 0.362019 | 0.999193 |
| AUD | 123.0 | 150.272727 | 149.66473 | 1.878229 | 0.361882 | 0.999533 |
| AUD | 203.0 | 234.538462 | 240.3172 | 3.07216 | 0.090412 | 0.999075 |
| AUD | 119.0 | 144.0 | 147.562784 | 2.96067 | 0.08057 | 0.999409 |
| AUD | 173.0 | 202.0 | 200.10625 | 2.304902 | 0.289539 | 0.999387 |
| AUD | 192.0 | 227.052632 | 223.635797 | 2.756063 | 0.127557 | 0.999208 |
| AUD | 119.0 | 169.75 | 175.581211 | 2.2165 | 0.250916 | 0.999421 |
| AUD | 125.0 | 143.055556 | 149.580614 | 1.583859 | 0.391274 | 0.999503 |
| AUD | 126.0 | 178.8 | 184.812402 | 1.785091 | 0.310924 | 0.999379 |
| AUD | 152.0 | 183.5 | 179.17085 | 1.862049 | 0.432113 | 0.999463 |
| AUD | 209.0 | 237.333333 | 236.598093 | 2.976377 | 0.12496 | 0.999199 |
| AUD | 146.0 | 177.5 | 183.094193 | 1.774339 | 0.305034 | 0.999329 |
| AUD | 168.0 | 192.391304 | 200.532712 | 2.360711 | 0.179722 | 0.999306 |
| AUD | 208.0 | 238.0 | 243.090789 | 2.089594 | 0.277088 | 0.999237 |
| AUD | 181.0 | 195.130435 | 197.588147 | 1.525246 | 0.510948 | 0.999522 |
| AUD | 204.0 | 257.04 | 254.993047 | 2.317201 | 0.169568 | 0.999 |
| HC | 114.0 | 143.0 | 176.913163 | 2.675723 | 0.121383 | 0.99937 |
| HC | 194.0 | 217.882353 | 217.777011 | 2.946134 | 0.12195 | 0.999337 |
| HC | 124.0 | 147.1 | 141.984382 | 2.326957 | 0.243541 | 0.999526 |
| HC | 207.0 | 230.076923 | 220.614138 | 2.851169 | 0.157971 | 0.99941 |
| HC | 261.0 | 290.64 | 286.624485 | 3.177272 | 0.121894 | 0.99905 |
| HC | 192.0 | 227.0 | 229.060832 | 3.170935 | 0.074732 | 0.999175 |
| HC | 252.0 | 275.428571 | 281.810315 | 3.229579 | 0.092233 | 0.998894 |
| HC | 195.0 | 215.666667 | 221.575191 | 2.318219 | 0.23686 | 0.999306 |
| HC | 205.0 | 272.363636 | 242.895616 | 3.085098 | 0.106808 | 0.999062 |
| HC | 224.0 | 255.0 | 252.013519 | 3.18419 | 0.130031 | 0.999296 |
| HC | 226.0 | 256.565217 | 255.699475 | 2.686709 | 0.158011 | 0.999191 |
| HC | 219.0 | 258.055556 | 252.201991 | 3.218181 | 0.100198 | 0.999115 |
| HC | 132.0 | 153.0 | 148.450529 | 3.143856 | 0.08148 | 0.999531 |
| HC | 166.0 | 238.066667 | 248.566418 | 1.516917 | 0.49171 | 0.999169 |
| HC | 196.0 | 287.866667 | 297.465092 | 2.414648 | 0.198399 | 0.998996 |
| HC | 147.0 | 201.0 | 178.145604 | 2.891778 | 0.110966 | 0.999342 |
| HC | 154.0 | 207.8125 | 231.682667 | 2.569724 | 0.14588 | 0.999128 |
| HC | 121.0 | 144.214286 | 144.474266 | 2.587615 | 0.14595 | 0.999481 |
| HC | 117.0 | 142.0 | 144.283236 | 2.816095 | 0.117834 | 0.999464 |
| HC | 104.0 | 129.3 | 149.306049 | 2.262125 | 0.195086 | 0.999537 |
| HC | 166.0 | 210.0 | 197.257068 | 3.169314 | 0.084469 | 0.999193 |
| HC | 257.0 | 297.037037 | 293.378835 | 2.74719 | 0.169342 | 0.998968 |
| HC | 163.0 | 182.5 | 185.868699 | 2.900523 | 0.12009 | 0.999367 |
| HC | 244.0 | 280.12 | 278.520251 | 2.801588 | 0.157513 | 0.999085 |
| HC | 130.0 | 151.0 | 159.538462 | 2.009135 | 0.305676 | 0.999475 |
| HC | 164.0 | 189.090909 | 183.093449 | 3.17537 | 0.081044 | 0.999426 |
| HC | 123.0 | 148.071429 | 177.181436 | 2.083402 | 0.281871 | 0.999468 |
| HC | 192.0 | 198.8 | 202.620902 | 3.15128 | 0.119784 | 0.999609 |
| HC | 142.0 | 194.5 | 250.194638 | 2.043043 | 0.30624 | 0.99927 |
| HC | 226.0 | 263.05 | 260.091725 | 3.431323 | 0.062838 | 0.999071 |
| HC | 291.0 | 342.130435 | 324.224835 | 3.16993 | 0.113682 | 0.998763 |
| HC | 176.0 | 197.0 | 198.517828 | 3.023142 | 0.098209 | 0.999348 |
| HC | 196.0 | 225.75 | 225.94274 | 2.025826 | 0.250486 | 0.999328 |
| HC | 312.0 | 382.0 | 355.68509 | 3.256103 | 0.159517 | 0.998591 |
| HC | 122.0 | 169.5 | 140.717619 | 3.131374 | 0.07129 | 0.999554 |
| Abbreviations: HC, healthy controls; AUD, alcohol use disorder patients | | | | | | |

**Table S5. Discriminatory OTUs between AUD Patients and Healthy Controls**

| **OTU** | **Taxonomic Assignment** | **Relative Abundance in AUD (%)** | | **Relative Abundance in HC (%)** | | **p-value** |
| --- | --- | --- | --- | --- | --- | --- |
|  |  | **Mean** | **Std. Deviation** | **Mean** | **Std. Deviation** |  |
| OTU1 | Firmicutes; Negativicutes;Selenomonadales;Veillonellaceae;Dialister;  Dialister_succinatiphilus | 2.307 | 11.569 | 2.380 | 9.273 | 0.958 |
| OTU10 | Firmicutes;Clostridia;Clostridiales;Ruminococcaceae;Gemmiger;  Gemmiger_formicilis | 1.089 | 3.232 | 1.834 | 5.144 | 0.019 |
| OTU100 | Firmicutes;Negativicutes;Selenomonadales;Veillonellaceae;Megasphaera | 0.000 | 0.001 | 0.037 | 0.176 | 0.988 |
| OTU101 | Bacteroidetes;Bacteroidia;Bacteroidales;Prevotellaceae;Prevotella | 0.000 | 0.000 | 0.067 | 0.398 | 0.354 |
| OTU102 | Firmicutes; Clostridia;Clostridiales;Lachnospiraceae;Blautia | 0.111 | 0.152 | 0.141 | 0.304 | 0.397 |
| OTU103 | Bacteroidetes; Bacteroidia;Bacteroidales;Prevotellaceae;Alloprevotella;  Alloprevotella_rava | 0.331 | 1.161 | 0.123 | 0.518 | 0.342 |
| OTU104 | Bacteria | 0.000 | 0.000 | 0.049 | 0.266 | 0.052 |
| OTU105 | Bacteroidetes ;Bacteroidia;Bacteroidales;Prevotellaceae;Prevotella;  Prevotella_stercorea | 0.081 | 0.314 | 0.089 | 0.331 | 0.876 |
| OTU106 | Firmicutes;Clostridia;Clostridiales | 0.003 | 0.014 | 0.046 | 0.183 | 0.282 |
| OTU107 | ActinoActinoBifidobacteriales;Bifidobacteriaceae;Bifidobacterium;  Bifidobacterium_longum | 0.067 | 0.214 | 0.038 | 0.147 | 0.747 |
| OTU108 | Firmicutes;Clostridia;Clostridiales;Ruminococcaceae;Ruminococcus;  Ruminococcus_bromii | 0.000 | 0.000 | 0.043 | 0.240 | 0.029 |
| OTU109 | Firmicutes;Clostridia;Clostridiales;Ruminococcaceae;Butyricicoccus;  Butyricicoccus_pullicaecorum | 0.206 | 0.436 | 0.185 | 0.206 | 0.233 |
| OTU11 | Bacteroidetes;Bacteroidia;Bacteroidales;Bacteroidaceae;Bacteroides;  Bacteroides_coprocola | 1.115 | 5.133 | 0.628 | 1.718 | 0.342 |
| OTU110 | Firmicutes;Clostridia;Clostridiales;Lachnospiraceae;Fusicatenibacter;  Fusicatenibacter_saccharivorans | 0.456 | 1.180 | 0.263 | 0.353 | 0.486 |
| OTU111 | Firmicutes;Clostridia;Clostridiales;Ruminococcaceae | 0.004 | 0.009 | 0.112 | 0.289 | 0.163 |
| OTU112 | Bacteroidetes;Bacteroidia;Bacteroidales;Rikenellaceae;Alistipes;  Alistipes_finegoldii | 0.000 | 0.000 | 0.019 | 0.111 | 0.336 |
| OTU113 | Bacteroidetes;Bacteroidia;Bacteroidales;Prevotellaceae;Prevotella;  Prevotella_copri | 0.036 | 0.110 | 0.282 | 1.308 | 0.663 |
| OTU114 | Bacteroidetes;Bacteroidia;Bacteroidales;Prevotellaceae;Prevotella | 0.000 | 0.000 | 0.033 | 0.167 | 0.052 |
| OTU115 | Firmicutes;Clostridia;Clostridiales;Ruminococcaceae | 0.002 | 0.005 | 0.065 | 0.218 | 0.057 |
| OTU116 | Firmicutes;Clostridia;Clostridiales;Ruminococcaceae;Gemmiger;  Gemmiger_formicilis | 0.691 | 2.299 | 1.075 | 1.655 | 0.006 |
| OTU117 | Firmicutes;Clostridia;Clostridiales;Lachnospiraceae | 0.038 | 0.062 | 0.068 | 0.191 | 0.855 |
| OTU118 | Bacteroidetes;Bacteroidia;Bacteroidales;Prevotellaceae;Prevotella;  Prevotella_copri | 16.509 | 22.680 | 11.106 | 18.516 | 0.610 |
| OTU119 | Firmicutes;Clostridia;Clostridiales | 0.000 | 0.001 | 0.033 | 0.179 | 0.705 |
| OTU12 | Firmicutes;Negativicutes;Selenomonadales;Veillonellaceae;Megasphaera;  Megasphaera_indica | 0.977 | 5.376 | 0.924 | 3.209 | 0.022 |
| OTU120 | Firmicutes;Clostridia;Clostridiales | 0.009 | 0.020 | 0.102 | 0.269 | 0.009 |
| OTU121 | Firmicutes | 0.003 | 0.008 | 0.100 | 0.311 | 0.019 |
| OTU122 | Bacteroidetes;Bacteroidia;Bacteroidales;Prevotellaceae;Prevotella | 0.068 | 0.380 | 0.000 | 0.001 | 0.317 |
| OTU123 | Firmicutes;Clostridia;Clostridiales;Peptostreptococcaceae;Intestinibacter;  Intestinibacter_bartlettii | 0.069 | 0.152 | 0.026 | 0.036 | 0.835 |
| OTU124 | Firmicutes;Clostridia;Clostridiales;Ruminococcaceae;Ruminococcus;  Ruminococcus_callidus | 0.028 | 0.068 | 0.059 | 0.132 | 0.313 |
| OTU125 | Firmicutes;Clostridia;Clostridiales | 0.000 | 0.002 | 0.045 | 0.235 | 0.088 |
| OTU126 | Firmicutes;Clostridia;Clostridiales;Ruminococcaceae;Ruminococcus;  Ruminococcus_albus | 0.018 | 0.059 | 0.067 | 0.157 | 0.056 |
| OTU127 | Firmicutes;Clostridia;Clostridiales;Eubacteriaceae;Eubacterium;  Eubacterium_coprostanoligenes | 0.013 | 0.039 | 0.056 | 0.126 | 0.033 |
| OTU128 | Firmicutes;Clostridia;Clostridiales;Lachnospiraceae | 0.080 | 0.091 | 0.210 | 0.224 | 0.009 |
| OTU129 | ProteoBetaproteoBurkholderiales;Sutterellaceae;Sutterella;  Sutterella_stercoricanis | 0.035 | 0.140 | 0.000 | 0.000 | 0.069 |
| OTU13 | Firmicutes;Clostridia;Clostridiales | 0.104 | 0.473 | 1.058 | 3.838 | 0.333 |
| OTU130 | Firmicutes;Clostridia;Clostridiales;Lachnospiraceae | 0.009 | 0.025 | 0.136 | 0.258 | 0.000 |
| OTU131 | Firmicutes;Clostridia;Clostridiales;Lachnospiraceae | 0.147 | 0.219 | 0.125 | 0.127 | 0.797 |
| OTU132 | Firmicutes;Clostridia;Clostridiales;Lachnospiraceae | 0.056 | 0.146 | 0.097 | 0.134 | 0.004 |
| OTU133 | Bacteroidetes;Bacteroidia;Bacteroidales;Porphyromonadaceae;  Porphyromonas;Porphyromonas_asaccharolytica | 0.036 | 0.196 | 0.000 | 0.002 | 0.185 |
| OTU134 | Firmicutes;Clostridia;Clostridiales;Peptoniphilaceae;Peptoniphilus;  Peptoniphilus_gorbachii | 0.026 | 0.148 | 0.000 | 0.001 | 0.544 |
| OTU135 | ProteoBetaproteoBurkholderiales;Sutterellaceae;Sutterella;  Sutterella_stercoricanis | 0.023 | 0.129 | 0.001 | 0.006 | 0.929 |
| OTU136 | Firmicutes;Clostridia;Clostridiales | 0.000 | 0.000 | 0.014 | 0.085 | 0.180 |
| OTU137 | Bacteroidetes;Bacteroidia;Bacteroidales;Prevotellaceae;Prevotella | 0.000 | 0.000 | 0.032 | 0.187 | 0.180 |
| OTU138 | Bacteroidetes;Bacteroidia;Bacteroidales;Porphyromonadaceae;  Porphyromonas;Porphyromonas_bennonis | 0.026 | 0.146 | 0.000 | 0.001 | 0.563 |
| OTU139 | Bacteroidetes;Bacteroidia;Bacteroidales;Bacteroidaceae;Bacteroides;  Bacteroides_dorei | 0.165 | 0.449 | 0.435 | 0.879 | 0.024 |
| OTU14 | Bacteroidetes;Bacteroidia;Bacteroidales;Bacteroidaceae;Bacteroides;  Bacteroides_stercoris | 1.958 | 6.056 | 1.344 | 2.551 | 0.460 |
| OTU140 | Firmicutes;Erysipelotrichia;Erysipelotrichales;Erysipelotrichaceae;  Turicibacter;Turicibacter_sanguinis | 0.027 | 0.075 | 0.028 | 0.105 | 0.363 |
| OTU141 | Bacteroidetes;Bacteroidia;Bacteroidales;Porphyromonadaceae;Barnesiella;Barnesiella_intestinihominis | 0.005 | 0.012 | 0.026 | 0.069 | 0.390 |
| OTU142 | Firmicutes;Clostridia;Clostridiales;Ruminococcaceae;Clostridium_IV;  Clostridium_leptum | 0.000 | 0.000 | 0.019 | 0.098 | 0.180 |
| OTU143 | Firmicutes;Clostridia;Clostridiales;Eubacteriaceae;Eubacterium | 0.013 | 0.076 | 0.000 | 0.000 | 0.142 |
| OTU144 | Bacteroidetes;Bacteroidia;Bacteroidales | 0.000 | 0.000 | 0.024 | 0.111 | 0.180 |
| OTU145 | Bacteroidetes;Bacteroidia;Bacteroidales;Rikenellaceae;Alistipes;  Alistipes_indistinctus | 0.004 | 0.016 | 0.017 | 0.071 | 0.327 |
| OTU146 | ProteoDeltaproteoDesulfovibrionales;Desulfovibrionaceae;Desulfovibrio;  Desulfovibrio_simplex | 0.012 | 0.048 | 0.052 | 0.142 | 0.018 |
| OTU147 | Bacteroidetes;Bacteroidia;Bacteroidales;Bacteroidaceae;Bacteroides;  Bacteroides_plebeius | 0.020 | 0.103 | 0.003 | 0.015 | 0.094 |
| OTU148 | Firmicutes;Clostridia;Clostridiales;Lachnospiraceae | 0.002 | 0.009 | 0.033 | 0.109 | 0.170 |
| OTU149 | Firmicutes;Clostridia;Clostridiales;Lachnospiraceae;Clostridium_XlVb;  Clostridium_lactatifermentans | 0.010 | 0.029 | 0.018 | 0.067 | 0.317 |
| OTU15 | Firmicutes;Clostridia;Clostridiales;Ruminococcaceae;Faecalibacterium;  Faecalibacterium_prausnitzii | 2.724 | 3.576 | 8.985 | 7.898 | 0.000 |
| OTU150 | Verrucomicrobia;Opitutae;Puniceicoccales;Puniceicoccaceae | 0.009 | 0.051 | 0.000 | 0.000 | 0.310 |
| OTU151 | Firmicutes;Clostridia;Clostridiales;Ruminococcaceae;Flavonifractor;  Flavonifractor_plautii | 0.031 | 0.063 | 0.048 | 0.076 | 0.091 |
| OTU152 | Firmicutes;Clostridia;Clostridiales;Ruminococcaceae | 0.008 | 0.020 | 0.022 | 0.071 | 0.141 |
| OTU153 | Firmicutes;Clostridia;Clostridiales;Clostridiales;Ezakiella;  Ezakiella_peruensis | 0.012 | 0.066 | 0.000 | 0.001 | 0.517 |
| OTU154 | Firmicutes;Clostridia;Clostridiales;Ruminococcaceae;Saccharofermentans;  Saccharofermentans_acetigenes | 0.023 | 0.129 | 0.000 | 0.000 | 0.310 |
| OTU155 | Firmicutes;Clostridia;Clostridiales;Lachnospiraceae;Clostridium_XlVa;  Clostridium_bolteae | 0.020 | 0.035 | 0.031 | 0.074 | 0.482 |
| OTU156 | Firmicutes;Clostridia;Clostridiales;Ruminococcaceae;Oscillibacter;  Oscillibacter_valericigenes | 0.024 | 0.078 | 0.061 | 0.098 | 0.059 |
| OTU157 | ProteoBetaproteoBurkholderiales;Sutterellaceae;Sutterella;  Sutterella_parvirubra | 0.011 | 0.062 | 0.016 | 0.088 | 0.624 |
| OTU158 | Firmicutes;Clostridia;Clostridiales;Ruminococcaceae | 0.000 | 0.001 | 0.018 | 0.079 | 0.038 |
| OTU159 | Firmicutes;Clostridia;Clostridiales;Ruminococcaceae;Clostridium_IV;  Clostridium_leptum | 0.041 | 0.070 | 0.026 | 0.037 | 0.963 |
| OTU16 | Bacteroidetes;Bacteroidia;Bacteroidales;Bacteroidaceae;Bacteroides;  Bacteroides_cellulosilyticus | 0.006 | 0.012 | 0.562 | 2.972 | 0.007 |
| OTU160 | Elusimicrobia;Elusimicrobia;Elusimicrobiales;Elusimicrobiaceae;  Elusimicrobium;Elusimicrobium_minutum | 0.008 | 0.043 | 0.000 | 0.000 | 0.310 |
| OTU161 | Firmicutes;Clostridia;Clostridiales;Ruminococcaceae;Butyricicoccus;  Butyricicoccus_pullicaecorum | 0.017 | 0.097 | 0.001 | 0.007 | 0.649 |
| OTU162 | Firmicutes;Negativicutes;Selenomonadales;Veillonellaceae;Allisonella;  Allisonella_histaminiformans | 0.053 | 0.292 | 0.016 | 0.075 | 0.813 |
| OTU163 | Firmicutes;Clostridia;Clostridiales;Lachnospiraceae | 0.084 | 0.112 | 0.147 | 0.169 | 0.031 |
| OTU164 | Firmicutes;Clostridia;Clostridiales;Ruminococcaceae;Clostridium_IV | 0.003 | 0.009 | 0.026 | 0.065 | 0.051 |
| OTU165 | Firmicutes;Erysipelotrichia;Erysipelotrichales;Erysipelotrichaceae;  Faecalicoccus | 0.024 | 0.070 | 0.002 | 0.007 | 0.063 |
| OTU166 | Bacteroidetes;Bacteroidia;Bacteroidales;Bacteroidaceae;Bacteroides;  Bacteroides_coprocola | 0.029 | 0.076 | 0.056 | 0.271 | 0.566 |
| OTU167 | Firmicutes;Clostridia;Clostridiales;Lachnospiraceae | 0.051 | 0.102 | 0.073 | 0.105 | 0.184 |
| OTU168 | Bacteroidetes;Bacteroidia;Bacteroidales;Porphyromonadaceae;Barnesiella;Barnesiella_intestinihominis | 0.000 | 0.001 | 0.011 | 0.064 | 0.540 |
| OTU169 | Bacteroidetes;Bacteroidia;Bacteroidales;Rikenellaceae;Alistipes;  Alistipes_indistinctus | 0.001 | 0.003 | 0.018 | 0.061 | 0.731 |
| OTU17 | Firmicutes;Erysipelotrichia;Erysipelotrichales;Erysipelotrichaceae;  Faecalicoccus;Faecalicoccus_acidiformans | 0.272 | 0.907 | 0.432 | 2.345 | 0.128 |
| OTU170 | Bacteroidetes;Bacteroidia;Bacteroidales;Prevotellaceae;Prevotella | 0.000 | 0.000 | 0.015 | 0.086 | 0.354 |
| OTU171 | CyanoCyanoUnclassified;Family_I;GpI | 0.000 | 0.001 | 0.012 | 0.047 | 0.075 |
| OTU172 | ProteoGammaproteoEnterobacteriales;Enterobacteriaceae;Raoultella;  Raoultella_ornithinolytica | 0.260 | 0.964 | 0.243 | 0.524 | 0.727 |
| OTU173 | Firmicutes;Clostridia;Clostridiales;Ruminococcaceae;Clostridium_IV | 0.000 | 0.001 | 0.008 | 0.038 | 0.344 |
| OTU174 | Firmicutes;Clostridia;Clostridiales;Clostridiales;Finegoldia;  Finegoldia_magna | 0.011 | 0.060 | 0.000 | 0.000 | 0.063 |
| OTU175 | Bacteroidetes;Bacteroidia;Bacteroidales;Prevotellaceae;Prevotella | 0.000 | 0.003 | 0.009 | 0.055 | 0.624 |
| OTU176 | Firmicutes;Clostridia;Clostridiales;Ruminococcaceae | 0.002 | 0.008 | 0.033 | 0.095 | 0.032 |
| OTU177 | Firmicutes;Clostridia;Clostridiales;Lachnospiraceae | 0.021 | 0.045 | 0.081 | 0.184 | 0.203 |
| OTU178 | Firmicutes;Clostridia;Clostridiales;Lachnospiraceae | 0.000 | 0.000 | 0.012 | 0.062 | 0.029 |
| OTU179 | Firmicutes;Clostridia;Clostridiales;Ruminococcaceae | 0.000 | 0.002 | 0.020 | 0.070 | 0.157 |
| OTU18 | Bacteroidetes;Bacteroidia;Bacteroidales;Bacteroidaceae;Bacteroides;  Bacteroides_coprophilus | 0.502 | 2.231 | 0.064 | 0.377 | 0.578 |
| OTU180 | Bacteroidetes;Bacteroidia;Bacteroidales;Porphyromonadaceae | 0.000 | 0.000 | 0.010 | 0.060 | 0.180 |
| OTU181 | Firmicutes;Clostridia;Clostridiales;Lachnospiraceae;Clostridium_XlVa | 0.003 | 0.004 | 0.034 | 0.102 | 0.024 |
| OTU182 | Firmicutes;Clostridia;Clostridiales | 0.000 | 0.000 | 0.017 | 0.064 | 0.096 |
| OTU183 | Firmicutes;Clostridia;Clostridiales;Ruminococcaceae;Faecalibacterium;  Faecalibacterium_prausnitzii | 0.008 | 0.037 | 0.000 | 0.001 | 0.495 |
| OTU184 | Firmicutes;Clostridia;Clostridiales;Ruminococcaceae | 0.009 | 0.039 | 0.005 | 0.012 | 0.722 |
| OTU185 | Bacteroidetes;Bacteroidia;Bacteroidales;Prevotellaceae;Prevotella | 0.000 | 0.000 | 0.011 | 0.067 | 0.354 |
| OTU186 | Firmicutes;Clostridia;Clostridiales;Ruminococcaceae;Oscillibacter | 0.009 | 0.020 | 0.021 | 0.042 | 0.115 |
| OTU187 | Bacteroidetes;Bacteroidia;Bacteroidales;Prevotellaceae;Prevotella;  Prevotella_copri | 0.000 | 0.000 | 0.094 | 0.553 | 0.336 |
| OTU188 | Firmicutes;Clostridia;Clostridiales;Ruminococcaceae | 0.000 | 0.000 | 0.007 | 0.033 | 0.029 |
| OTU189 | Firmicutes;Clostridia;Clostridiales;Lachnospiraceae;Anaerostipes | 0.000 | 0.000 | 0.012 | 0.068 | 0.096 |
| OTU19 | Firmicutes;Clostridia;Clostridiales;Lachnospiraceae;  Lachnospiracea_incertae_sedis;Eubacterium_eligens | 0.425 | 0.612 | 1.800 | 3.029 | 0.069 |
| OTU190 | Bacteroidetes;Bacteroidia;Bacteroidales;Porphyromonadaceae;Barnesiella;Barnesiella_intestinihominis | 0.000 | 0.000 | 0.004 | 0.026 | 0.354 |
| OTU191 | Firmicutes;Clostridia;Clostridiales;Ruminococcaceae;Ruminococcus;  Ruminococcus_flavefaciens | 0.000 | 0.000 | 0.006 | 0.038 | 0.354 |
| OTU192 | Firmicutes | 0.000 | 0.000 | 0.008 | 0.040 | 0.096 |
| OTU193 | Bacteroidetes;Bacteroidia;Bacteroidales;Porphyromonadaceae;Coprobacter;Coprobacter_fastidiosus | 0.002 | 0.010 | 0.008 | 0.030 | 0.120 |
| OTU194 | Firmicutes;Clostridia;Clostridiales;Ruminococcaceae | 0.000 | 0.000 | 0.006 | 0.036 | 0.180 |
| OTU195 | Firmicutes;Clostridia;Clostridiales;Ruminococcaceae | 0.005 | 0.026 | 0.007 | 0.023 | 0.524 |
| OTU196 | Bacteroidetes;Bacteroidia;Bacteroidales;Porphyromonadaceae | 0.000 | 0.000 | 0.007 | 0.041 | 0.354 |
| OTU197 | Firmicutes;Clostridia;Clostridiales;Lachnospiraceae;Howardella;  Howardella_ureilytica | 0.007 | 0.031 | 0.004 | 0.010 | 0.513 |
| OTU198 | Bacteroidetes;Bacteroidia;Bacteroidales;Porphyromonadaceae;  Parabacteroides;Parabacteroides_faecis | 0.003 | 0.009 | 0.022 | 0.080 | 0.836 |
| OTU199 | Bacteroidetes;Bacteroidia;Bacteroidales;Prevotellaceae;Prevotella;  Prevotella_copri | 0.094 | 0.203 | 0.016 | 0.050 | 0.031 |
| OTU2 | Bacteroidetes;Bacteroidia;Bacteroidales;Prevotellaceae;Prevotella;  Prevotella_copri | 5.251 | 10.983 | 1.906 | 4.759 | 0.247 |
| OTU20 | ProteoBetaproteoBurkholderiales;Sutterellaceae;Parasutterella;  Parasutterella_excrementihominis | 1.055 | 2.456 | 0.286 | 0.576 | 0.261 |
| OTU200 | ActinoActinoActinomycetales;Actinomycetaceae;Varibaculum;  Varibaculum_cambriense | 0.005 | 0.027 | 0.000 | 0.000 | 0.310 |
| OTU201 | Firmicutes;Clostridia;Clostridiales;Ruminococcaceae | 0.000 | 0.000 | 0.007 | 0.027 | 0.096 |
| OTU202 | ProteoEpsilonproteoCampylobacterales;Campylobacteraceae;  Campylobacter;Campylobacter_gracilis | 0.006 | 0.035 | 0.000 | 0.000 | 0.310 |
| OTU203 | Bacteroidetes;Bacteroidia;Bacteroidales;Porphyromonadaceae;  Butyricimonas;Butyricimonas_virosa | 0.007 | 0.026 | 0.002 | 0.007 | 0.657 |
| OTU204 | Bacteroidetes;Bacteroidia;Bacteroidales;Porphyromonadaceae;  Butyricimonas;Butyricimonas_virosa | 0.007 | 0.025 | 0.003 | 0.006 | 0.681 |
| OTU205 | Firmicutes;Clostridia;Clostridiales;Peptoniphilaceae;Peptoniphilus;  Peptoniphilus_coxii | 0.013 | 0.072 | 0.000 | 0.000 | 0.278 |
| OTU206 | Firmicutes;Negativicutes;Selenomonadales;Acidaminococcaceae | 0.000 | 0.000 | 0.008 | 0.045 | 0.354 |
| OTU207 | Firmicutes;Clostridia;Clostridiales;Lachnospiraceae;Clostridium_XlVa | 0.000 | 0.000 | 0.006 | 0.034 | 0.052 |
| OTU208 | Firmicutes;Clostridia;Clostridiales;Lachnospiraceae;Clostridium_XlVb | 0.008 | 0.024 | 0.015 | 0.027 | 0.449 |
| OTU209 | Candidatus_SacchariUnclassified;Unclassified;Unclassified;  SacchariTM7_phylum | 0.015 | 0.036 | 0.005 | 0.009 | 0.913 |
| OTU21 | Firmicutes;Clostridia;Clostridiales;Peptostreptococcaceae;Romboutsia;  Romboutsia_sedimentorum | 1.100 | 2.374 | 0.281 | 0.449 | 0.098 |
| OTU210 | ProteoGammaproteoPasteurellales;Pasteurellaceae | 0.004 | 0.020 | 0.015 | 0.069 | 0.088 |
| OTU211 | Firmicutes;Clostridia;Clostridiales;Lachnospiraceae | 0.001 | 0.002 | 0.018 | 0.052 | 0.012 |
| OTU212 | Firmicutes;Bacilli;Lactobacillales;Streptococcaceae;Streptococcus;  Streptococcus_parasanguinis | 0.046 | 0.108 | 0.049 | 0.094 | 0.081 |
| OTU213 | Firmicutes;Erysipelotrichia;Erysipelotrichales;Erysipelotrichaceae;  Clostridium_XVIII;Clostridium_ramosum | 0.011 | 0.033 | 0.006 | 0.016 | 0.457 |
| OTU214 | Firmicutes;Clostridia;Clostridiales;Ruminococcaceae | 0.000 | 0.000 | 0.007 | 0.034 | 0.004 |
| OTU215 | Firmicutes;Negativicutes;Selenomonadales;Veillonellaceae;Veillonella | 0.009 | 0.053 | 0.000 | 0.000 | 0.310 |
| OTU216 | Bacteroidetes;Bacteroidia;Bacteroidales;Bacteroidaceae;Bacteroides;  Bacteroides_vulgatus | 0.766 | 1.777 | 0.689 | 1.025 | 0.427 |
| OTU217 | Bacteroidetes;Bacteroidia;Bacteroidales;Porphyromonadaceae;  Porphyromonas;Porphyromonas_somerae | 0.008 | 0.047 | 0.000 | 0.001 | 0.891 |
| OTU218 | Firmicutes;Clostridia;Clostridiales | 0.000 | 0.000 | 0.005 | 0.027 | 0.052 |
| OTU219 | Firmicutes;Clostridia;Clostridiales;Clostridiales;Anaerococcus | 0.007 | 0.042 | 0.000 | 0.000 | 0.517 |
| OTU22 | Bacteroidetes;Bacteroidia;Bacteroidales;Bacteroidaceae;Bacteroides;  Bacteroides_massiliensis | 1.294 | 4.569 | 0.801 | 1.824 | 0.533 |
| OTU220 | Firmicutes;Clostridia;Clostridiales;Ruminococcaceae;Faecalibacterium;  Faecalibacterium_prausnitzii | 0.000 | 0.001 | 0.012 | 0.042 | 0.059 |
| OTU221 | Bacteria | 0.000 | 0.001 | 0.010 | 0.048 | 0.336 |
| OTU222 | Firmicutes;Clostridia;Clostridiales | 0.000 | 0.000 | 0.008 | 0.034 | 0.180 |
| OTU223 | Firmicutes;Clostridia;Clostridiales;Ruminococcaceae | 0.005 | 0.022 | 0.002 | 0.005 | 0.206 |
| OTU224 | Bacteroidetes;Bacteroidia;Bacteroidales;Bacteroidaceae;Bacteroides;  Bacteroides_coprophilus | 0.041 | 0.223 | 0.001 | 0.005 | 0.265 |
| OTU225 | Firmicutes;Clostridia;Clostridiales;Ruminococcaceae | 0.009 | 0.024 | 0.011 | 0.027 | 0.705 |
| OTU226 | Firmicutes;Negativicutes;Selenomonadales;Veillonellaceae;Megamonas;  Megamonas_funiformis | 1.525 | 4.696 | 2.157 | 5.170 | 0.231 |
| OTU227 | Firmicutes;Clostridia;Clostridiales;Ruminococcaceae | 0.000 | 0.000 | 0.005 | 0.021 | 0.102 |
| OTU228 | Firmicutes;Clostridia;Clostridiales;Clostridiales | 0.002 | 0.005 | 0.011 | 0.030 | 0.061 |
| OTU229 | Firmicutes;Negativicutes;Selenomonadales;Veillonellaceae;Megasphaera | 0.016 | 0.066 | 0.008 | 0.032 | 0.885 |
| OTU23 | Firmicutes;Clostridia;Clostridiales;Lachnospiraceae;Ruminococcus2;  Ruminococcus_faecis | 1.881 | 3.418 | 1.242 | 3.160 | 0.104 |
| OTU230 | ProteoAlphaproteoSphingomonadales;Sphingomonadaceae;Sphingomonas;Sphingomonas_paucimobilis | 0.005 | 0.031 | 0.000 | 0.001 | 0.939 |
| OTU231 | Firmicutes;Clostridia;Clostridiales;Ruminococcaceae;Intestinimonas;  Intestinimonas_butyriciproducens | 0.008 | 0.020 | 0.017 | 0.025 | 0.094 |
| OTU232 | Bacteroidetes;Bacteroidia;Bacteroidales;Prevotellaceae;Prevotella | 0.005 | 0.019 | 0.000 | 0.001 | 0.582 |
| OTU233 | Firmicutes;Clostridia;Clostridiales;Ruminococcaceae;Clostridium_III | 0.000 | 0.000 | 0.004 | 0.015 | 0.052 |
| OTU234 | Firmicutes;Clostridia;Clostridiales;Ruminococcaceae | 0.001 | 0.003 | 0.009 | 0.025 | 0.011 |
| OTU235 | Firmicutes;Clostridia;Clostridiales;Clostridiales | 0.004 | 0.025 | 0.000 | 0.000 | 0.310 |
| OTU236 | Firmicutes;Erysipelotrichia;Erysipelotrichales;Erysipelotrichaceae;  Coprobacillus;Coprobacillus_cateniformis | 0.004 | 0.016 | 0.000 | 0.000 | 0.018 |
| OTU237 | Bacteroidetes;Bacteroidia;Bacteroidales;Prevotellaceae;Prevotella;  Prevotella_copri | 0.000 | 0.000 | 0.060 | 0.237 | 0.052 |
| OTU238 | Firmicutes;Erysipelotrichia;Erysipelotrichales;Erysipelotrichaceae;  Clostridium_XVIII;Clostridium_spiroforme | 0.005 | 0.017 | 0.001 | 0.003 | 0.406 |
| OTU239 | Firmicutes;Clostridia;Clostridiales;Lachnospiraceae | 0.046 | 0.088 | 0.147 | 0.516 | 0.200 |
| OTU24 | ActinoActinoBifidobacteriales;Bifidobacteriaceae;Bifidobacterium;  Bifidobacterium_catenulatum | 0.354 | 1.081 | 0.325 | 1.432 | 0.162 |
| OTU240 | ProteoDeltaproteoDesulfovibrionales;Desulfovibrionaceae;Desulfovibrio;  Desulfovibrio_piger | 0.002 | 0.008 | 0.004 | 0.015 | 0.988 |
| OTU241 | ActinoActinoCoriobacteriales;Coriobacteriaceae;Collinsella;  Collinsella_aerofaciens | 0.006 | 0.016 | 0.001 | 0.002 | 0.218 |
| OTU242 | Firmicutes;Clostridia;Clostridiales;Peptoniphilaceae;Peptoniphilus | 0.004 | 0.020 | 0.000 | 0.000 | 0.310 |
| OTU243 | Bacteria | 0.000 | 0.000 | 0.003 | 0.015 | 0.354 |
| OTU244 | Firmicutes;Clostridia;Clostridiales;Ruminococcaceae | 0.000 | 0.002 | 0.009 | 0.035 | 0.157 |
| OTU245 | Firmicutes;Clostridia;Clostridiales;Ruminococcaceae | 0.000 | 0.000 | 0.003 | 0.019 | 0.354 |
| OTU246 | ProteoBetaproteoBurkholderiales | 0.000 | 0.000 | 0.005 | 0.028 | 0.354 |
| OTU247 | Firmicutes;Clostridia;Clostridiales;Ruminococcaceae | 0.006 | 0.010 | 0.010 | 0.022 | 0.879 |
| OTU248 | Bacteroidetes | 0.003 | 0.017 | 0.000 | 0.000 | 0.310 |
| OTU249 | Lentisphaerae;Lentisphaeria;Victivallales;Victivallaceae;Victivallis;  Victivallis_vadensis | 0.002 | 0.013 | 0.000 | 0.000 | 0.517 |
| OTU25 | Bacteroidetes;Bacteroidia;Bacteroidales;Prevotellaceae;Alloprevotella;  Alloprevotella_rava | 0.263 | 1.489 | 0.095 | 0.561 | 0.470 |
| OTU250 | ActinoActinoActinomycetales;Micrococcaceae;Rothia;  Rothia_mucilaginosa | 0.006 | 0.023 | 0.003 | 0.004 | 0.066 |
| OTU251 | Bacteroidetes;Bacteroidia;Bacteroidales | 0.000 | 0.002 | 0.003 | 0.019 | 0.963 |
| OTU252 | Bacteroidetes;Bacteroidia;Bacteroidales;Porphyromonadaceae;Odoribacter;Odoribacter_laneus | 0.015 | 0.027 | 0.017 | 0.024 | 0.720 |
| OTU253 | Firmicutes;Clostridia;Clostridiales;Ruminococcaceae;Clostridium_IV;  Clostridium_leptum | 0.001 | 0.002 | 0.006 | 0.016 | 0.070 |
| OTU254 | Bacteroidetes;Bacteroidia;Bacteroidales;Prevotellaceae;Prevotella | 0.000 | 0.000 | 0.003 | 0.016 | 0.180 |
| OTU255 | Firmicutes;Clostridia;Clostridiales;Lachnospiraceae | 0.005 | 0.012 | 0.018 | 0.024 | 0.044 |
| OTU256 | Firmicutes;Clostridia;Clostridiales;Eubacteriaceae;Eubacterium;  Eubacterium_coprostanoligenes | 0.003 | 0.016 | 0.000 | 0.000 | 0.310 |
| OTU257 | Firmicutes | 0.000 | 0.000 | 0.004 | 0.025 | 0.180 |
| OTU258 | Bacteroidetes;Bacteroidia;Bacteroidales;Rikenellaceae;Alistipes;  Alistipes_indistinctus | 0.003 | 0.011 | 0.005 | 0.011 | 0.185 |
| OTU259 | Bacteroidetes;Bacteroidia;Bacteroidales;Bacteroidaceae;Bacteroides;  Bacteroides_plebeius | 0.055 | 0.262 | 0.061 | 0.226 | 0.080 |
| OTU26 | Firmicutes;Negativicutes;Selenomonadales;Veillonellaceae;Dialister;  Dialister_invisus | 0.084 | 0.352 | 0.286 | 1.305 | 0.500 |
| OTU260 | Bacteroidetes;Bacteroidia;Bacteroidales;Rikenellaceae;Alistipes;  Alistipes_shahii | 0.010 | 0.021 | 0.044 | 0.090 | 0.391 |
| OTU261 | Firmicutes;Clostridia;Clostridiales;Lachnospiraceae;Coprococcus;  Coprococcus_catus | 0.042 | 0.080 | 0.035 | 0.044 | 0.985 |
| OTU262 | Firmicutes;Clostridia;Clostridiales;Lachnospiraceae;Clostridium_XlVa;  Clostridium_scindens | 0.006 | 0.010 | 0.005 | 0.017 | 0.066 |
| OTU263 | Bacteroidetes;Bacteroidia;Bacteroidales;Rikenellaceae;Alistipes;  Alistipes_shahii | 0.001 | 0.006 | 0.004 | 0.013 | 0.164 |
| OTU264 | Firmicutes;Clostridia;Clostridiales;Clostridiales;Anaerococcus;  Anaerococcus_octavius | 0.003 | 0.016 | 0.000 | 0.000 | 0.310 |
| OTU265 | Firmicutes;Clostridia;Clostridiales;Ruminococcaceae | 0.000 | 0.000 | 0.004 | 0.018 | 0.186 |
| OTU266 | Firmicutes;Clostridia;Clostridiales;Lachnospiraceae | 0.000 | 0.000 | 0.005 | 0.029 | 0.354 |
| OTU267 | Firmicutes;Negativicutes;Selenomonadales;Veillonellaceae;Dialister;  Dialister_propionicifaciens | 0.005 | 0.026 | 0.000 | 0.001 | 0.193 |
| OTU268 | Firmicutes;Negativicutes;Selenomonadales;Veillonellaceae;Megasphaera | 0.000 | 0.001 | 0.093 | 0.545 | 0.599 |
| OTU269 | Firmicutes;Bacilli;Lactobacillales;Lactobacillaceae;Lactobacillus;  Lactobacillus_plantarum | 0.001 | 0.004 | 0.004 | 0.020 | 0.973 |
| OTU27 | Firmicutes;Clostridia;Clostridiales;Lachnospiraceae;Coprococcus;  Coprococcus_eutactus | 0.019 | 0.078 | 0.887 | 2.369 | 0.009 |
| OTU270 | Bacteroidetes;Bacteroidia;Bacteroidales;Bacteroidaceae;Bacteroides;  Bacteroides_coprocola | 0.002 | 0.008 | 0.083 | 0.477 | 0.397 |
| OTU271 | Firmicutes | 0.000 | 0.000 | 0.002 | 0.012 | 0.599 |
| OTU272 | Bacteroidetes;Bacteroidia;Bacteroidales;Porphyromonadaceae;  Porphyromonas | 0.003 | 0.016 | 0.000 | 0.000 | 0.142 |
| OTU273 | Firmicutes;Negativicutes;Selenomonadales;Veillonellaceae;Megamonas | 0.027 | 0.056 | 0.027 | 0.114 | 0.389 |
| OTU274 | Bacteroidetes;Bacteroidia;Bacteroidales;Prevotellaceae;Alloprevotella;  Alloprevotella_rava | 0.000 | 0.000 | 0.005 | 0.027 | 0.354 |
| OTU275 | Bacteroidetes;Bacteroidia;Bacteroidales;Porphyromonadaceae;  Butyricimonas;Butyricimonas_faecihominis | 0.003 | 0.008 | 0.006 | 0.013 | 0.475 |
| OTU276 | Firmicutes;Clostridia;Clostridiales;Clostridiales;Mogibacterium;  Mogibacterium_timidum | 0.005 | 0.026 | 0.000 | 0.000 | 0.310 |
| OTU277 | Firmicutes;Clostridia;Clostridiales;Lachnospiraceae;Clostridium_XlVa;  Clostridium_bolteae | 0.062 | 0.125 | 0.177 | 0.325 | 0.111 |
| OTU278 | Firmicutes;Bacilli;Lactobacillales;Lactobacillaceae;Lactobacillus;  Lactobacillus_paralimentarius | 0.003 | 0.015 | 0.002 | 0.008 | 0.929 |
| OTU279 | Firmicutes;Clostridia;Clostridiales;Ruminococcaceae | 0.000 | 0.002 | 0.006 | 0.022 | 0.199 |
| OTU28 | Firmicutes;Negativicutes;Selenomonadales;Acidaminococcaceae;  Phascolarctobacterium;Phascolarctobacterium_faecium | 0.430 | 1.271 | 1.054 | 1.683 | 0.126 |
| OTU280 | Firmicutes;Negativicutes;Selenomonadales;Acidaminococcaceae;  Acidaminococcus;Acidaminococcus_intestini | 0.004 | 0.015 | 0.001 | 0.005 | 0.562 |
| OTU281 | Firmicutes;Bacilli;Lactobacillales;Carnobacteriaceae;Granulicatella;  Granulicatella_adiacens | 0.006 | 0.014 | 0.003 | 0.004 | 0.727 |
| OTU282 | Firmicutes;Clostridia;Clostridiales;Ruminococcaceae;Intestinimonas;  Intestinimonas_butyriciproducens | 0.001 | 0.003 | 0.002 | 0.012 | 0.970 |
| OTU283 | Firmicutes;Clostridia;Clostridiales;Clostridiales;Anaerococcus;  Anaerococcus_octavius | 0.004 | 0.023 | 0.000 | 0.000 | 0.310 |
| OTU284 | ProteoBetaproteoBurkholderiales | 0.000 | 0.000 | 0.002 | 0.011 | 0.354 |
| OTU285 | Firmicutes;Bacilli;Lactobacillales;Lactobacillaceae;Lactobacillus;  Lactobacillus_ruminis | 0.002 | 0.007 | 0.002 | 0.014 | 0.540 |
| OTU286 | Bacteroidetes;Bacteroidia;Bacteroidales;Bacteroidaceae;Bacteroides;  Bacteroides_nordii | 0.002 | 0.005 | 0.015 | 0.038 | 0.079 |
| OTU287 | Firmicutes;Clostridia;Clostridiales;Ruminococcaceae | 0.000 | 0.000 | 0.003 | 0.018 | 0.180 |
| OTU288 | ActinoActinoCoriobacteriales;Coriobacteriaceae;Olsenella | 0.002 | 0.011 | 0.000 | 0.000 | 0.310 |
| OTU289 | Firmicutes;Clostridia;Clostridiales;Ruminococcaceae | 0.000 | 0.001 | 0.003 | 0.014 | 0.599 |
| OTU29 | Firmicutes;Clostridia;Clostridiales;Lachnospiraceae;Clostridium_XlVa | 0.915 | 1.548 | 1.202 | 2.123 | 0.846 |
| OTU290 | Firmicutes;Clostridia;Clostridiales;Lachnospiraceae;Blautia;  Blautia_schinkii | 0.017 | 0.056 | 0.001 | 0.002 | 0.054 |
| OTU291 | FusoFusobacteriia;Fusobacteriales;Fusobacteriaceae | 0.002 | 0.011 | 0.000 | 0.000 | 0.142 |
| OTU292 | Firmicutes;Clostridia;Clostridiales;Lachnospiraceae | 0.120 | 0.184 | 0.067 | 0.098 | 0.691 |
| OTU293 | Firmicutes;Negativicutes;Selenomonadales;Veillonellaceae;Megasphaera;  Megasphaera_indica | 0.017 | 0.093 | 0.000 | 0.001 | 0.517 |
| OTU294 | Firmicutes;Negativicutes;Selenomonadales;Veillonellaceae;Negativicoccus;Negativicoccus_succinicivorans | 0.002 | 0.012 | 0.000 | 0.000 | 0.310 |
| OTU295 | Bacteroidetes;Bacteroidia;Bacteroidales;Bacteroidaceae;Bacteroides | 0.000 | 0.000 | 0.003 | 0.017 | 0.354 |
| OTU296 | Lentisphaerae;Oligosphaeria;Oligosphaerales;Oligosphaeraceae;  Oligosphaera;Oligosphaera_ethanolica | 0.000 | 0.000 | 0.003 | 0.015 | 0.354 |
| OTU297 | Firmicutes;Clostridia;Clostridiales;Ruminococcaceae | 0.000 | 0.002 | 0.003 | 0.013 | 0.710 |
| OTU298 | Firmicutes;Clostridia;Clostridiales | 0.000 | 0.000 | 0.003 | 0.010 | 0.016 |
| OTU299 | Firmicutes;Clostridia;Clostridiales;Peptoniphilaceae;Peptoniphilus;  Peptoniphilus_duerdenii | 0.002 | 0.010 | 0.000 | 0.002 | 0.649 |
| OTU3 | Firmicutes;Negativicutes;Selenomonadales;Veillonellaceae;Megamonas;  Megamonas_funiformis | 7.706 | 16.297 | 3.960 | 9.920 | 0.069 |
| OTU30 | Bacteroidetes;Bacteroidia;Bacteroidales;Prevotellaceae;Prevotella;  Prevotella_stercorea | 0.731 | 3.411 | 0.129 | 0.470 | 0.798 |
| OTU300 | Firmicutes;Clostridia;Clostridiales;Ruminococcaceae;Oscillibacter;  Oscillibacter_ruminantium | 0.001 | 0.002 | 0.004 | 0.010 | 0.340 |
| OTU301 | Bacteroidetes;Bacteroidia;Bacteroidales;Porphyromonadaceae;  Parabacteroides;Parabacteroides_goldsteinii | 0.002 | 0.010 | 0.002 | 0.005 | 0.431 |
| OTU302 | Firmicutes;Clostridia;Clostridiales;Ruminococcaceae | 0.000 | 0.000 | 0.001 | 0.008 | 0.354 |
| OTU303 | Firmicutes;Clostridia;Clostridiales;Lachnospiraceae | 0.045 | 0.105 | 0.013 | 0.022 | 0.255 |
| OTU304 | Firmicutes;Clostridia;Clostridiales;Ruminococcaceae | 0.000 | 0.000 | 0.003 | 0.011 | 0.096 |
| OTU305 | Firmicutes;Negativicutes;Selenomonadales;Veillonellaceae;Megasphaera;  Megasphaera_indica | 0.006 | 0.031 | 0.070 | 0.404 | 0.455 |
| OTU306 | Firmicutes;Clostridia;Clostridiales;Clostridiales | 0.001 | 0.002 | 0.005 | 0.016 | 0.419 |
| OTU307 | Bacteroidetes;Bacteroidia;Bacteroidales;Bacteroidaceae;Bacteroides;  Bacteroides_sartorii | 0.000 | 0.000 | 0.002 | 0.010 | 0.354 |
| OTU308 | Firmicutes;Clostridia;Clostridiales;Ruminococcaceae;Clostridium_IV;  Clostridium_leptum | 0.003 | 0.009 | 0.002 | 0.005 | 0.673 |
| OTU309 | FusoFusobacteriia;Fusobacteriales;Fusobacteriaceae;Fusobacterium;  Fusobacterium_varium | 0.511 | 1.496 | 0.106 | 0.451 | 0.344 |
| OTU31 | Firmicutes;Negativicutes;Selenomonadales;Veillonellaceae;Mitsuokella;  Mitsuokella_multacida | 0.876 | 4.025 | 0.031 | 0.140 | 0.223 |
| OTU310 | Bacteroidetes;Bacteroidia;Bacteroidales;Prevotellaceae;Prevotella;  Prevotella_copri | 0.000 | 0.001 | 0.024 | 0.141 | 0.582 |
| OTU311 | Firmicutes;Clostridia;Clostridiales | 0.000 | 0.000 | 0.003 | 0.014 | 0.180 |
| OTU312 | Firmicutes;Clostridia;Clostridiales | 0.000 | 0.000 | 0.002 | 0.009 | 0.180 |
| OTU313 | Firmicutes;Clostridia;Clostridiales | 0.000 | 0.000 | 0.001 | 0.007 | 0.354 |
| OTU314 | Firmicutes;Clostridia;Clostridiales;Ruminococcaceae | 0.000 | 0.002 | 0.004 | 0.011 | 0.065 |
| OTU315 | Bacteroidetes;Bacteroidia;Bacteroidales;Prevotellaceae;Prevotella | 0.084 | 0.282 | 0.091 | 0.340 | 0.881 |
| OTU316 | Bacteroidetes;Bacteroidia;Bacteroidales;Prevotellaceae;Prevotella;  Prevotella_copri | 0.156 | 0.545 | 0.048 | 0.167 | 0.887 |
| OTU317 | Firmicutes;Clostridia;Clostridiales;Ruminococcaceae;Flavonifractor;  Flavonifractor_plautii | 0.008 | 0.027 | 0.008 | 0.020 | 0.462 |
| OTU318 | Firmicutes;Clostridia;Clostridiales;Lachnospiraceae | 0.009 | 0.014 | 0.012 | 0.015 | 0.234 |
| OTU319 | Firmicutes;Erysipelotrichia;Erysipelotrichales;Erysipelotrichaceae;  Faecalicoccus;Faecalicoccus_pleomorphus | 0.000 | 0.001 | 0.002 | 0.010 | 0.092 |
| OTU32 | Firmicutes;Clostridia;Clostridiales;Lachnospiraceae;  Lachnospiracea_incertae_sedis;Ruminococcus_gnavus | 0.675 | 1.504 | 0.546 | 1.683 | 0.008 |
| OTU320 | Bacteroidetes;Bacteroidia;Bacteroidales;Prevotellaceae;Prevotella;  Prevotella_copri | 0.000 | 0.001 | 0.095 | 0.562 | 0.599 |
| OTU321 | ProteoEpsilonproteoCampylobacterales;Helicobacteraceae;Helicobacter;  Helicobacter_typhlonius | 0.001 | 0.003 | 0.001 | 0.007 | 0.624 |
| OTU322 | Bacteroidetes;Bacteroidia;Bacteroidales;Porphyromonadaceae;Barnesiella;Barnesiella_intestinihominis | 0.000 | 0.002 | 0.001 | 0.008 | 0.540 |
| OTU323 | Bacteroidetes;Bacteroidia;Bacteroidales;Bacteroidaceae;Bacteroides;  Bacteroides_ovatus | 0.052 | 0.131 | 0.203 | 0.532 | 0.022 |
| OTU324 | Firmicutes;Bacilli;Lactobacillales;Streptococcaceae;Lactococcus;  Lactococcus_lactis | 0.001 | 0.007 | 0.000 | 0.001 | 0.915 |
| OTU325 | Firmicutes;Clostridia;Clostridiales | 0.000 | 0.000 | 0.002 | 0.013 | 0.983 |
| OTU326 | Bacteroidetes;Bacteroidia;Bacteroidales;Porphyromonadaceae;  Parabacteroides | 0.002 | 0.010 | 0.001 | 0.007 | 0.949 |
| OTU327 | Firmicutes;Clostridia;Clostridiales | 0.000 | 0.000 | 0.003 | 0.011 | 0.055 |
| OTU328 | Firmicutes;Clostridia;Clostridiales;Ruminococcaceae | 0.000 | 0.000 | 0.002 | 0.011 | 0.180 |
| OTU329 | Bacteroidetes;Bacteroidia;Bacteroidales;Prevotellaceae;Prevotella;  Prevotella_copri | 0.074 | 0.351 | 0.001 | 0.003 | 0.516 |
| OTU33 | Firmicutes;Clostridia;Clostridiales;Lachnospiraceae;Clostridium_XlVa;  Clostridium_nexile | 0.444 | 1.319 | 0.091 | 0.112 | 0.104 |
| OTU330 | Firmicutes;Clostridia;Clostridiales | 0.000 | 0.000 | 0.002 | 0.011 | 0.096 |
| OTU331 | Bacteroidetes;Bacteroidia;Bacteroidales;Prevotellaceae;Prevotella;  Prevotella_copri | 0.030 | 0.058 | 0.025 | 0.053 | 0.280 |
| OTU332 | Firmicutes;Clostridia;Clostridiales;Ruminococcaceae;Anaerotruncus;  Anaerotruncus_colihominis | 0.001 | 0.003 | 0.004 | 0.012 | 0.597 |
| OTU333 | Firmicutes;Clostridia;Clostridiales;Peptoniphilaceae;Peptoniphilus;  Peptoniphilus_asaccharolyticus | 0.004 | 0.022 | 0.000 | 0.000 | 0.142 |
| OTU334 | Firmicutes;Clostridia;Clostridiales;Christensenellaceae;Christensenella;  Christensenella_minuta | 0.000 | 0.001 | 0.002 | 0.009 | 0.096 |
| OTU335 | Firmicutes;Bacilli;Lactobacillales;Leuconostocaceae;Weissella;  Weissella_confusa | 0.006 | 0.018 | 0.002 | 0.008 | 0.486 |
| OTU336 | Firmicutes;Clostridia;Clostridiales;Ruminococcaceae;Flavonifractor;  Flavonifractor_plautii | 0.003 | 0.008 | 0.006 | 0.008 | 0.010 |
| OTU337 | Firmicutes;Clostridia;Clostridiales | 0.000 | 0.001 | 0.002 | 0.007 | 0.108 |
| OTU338 | Bacteroidetes;Bacteroidia;Bacteroidales;Prevotellaceae | 0.000 | 0.001 | 0.003 | 0.013 | 0.599 |
| OTU339 | Firmicutes;Clostridia;Clostridiales;Ruminococcaceae | 0.001 | 0.005 | 0.003 | 0.011 | 0.993 |
| OTU34 | Bacteroidetes;Bacteroidia;Bacteroidales;Bacteroidaceae;Bacteroides;  Bacteroides_ovatus | 0.517 | 1.060 | 1.305 | 2.244 | 0.001 |
| OTU340 | Bacteria | 0.001 | 0.007 | 0.000 | 0.000 | 0.310 |
| OTU341 | Firmicutes;Erysipelotrichia;Erysipelotrichales;Erysipelotrichaceae;  Faecalicoccus;Faecalicoccus_pleomorphus | 0.002 | 0.008 | 0.000 | 0.000 | 0.016 |
| OTU342 | Firmicutes;Erysipelotrichia;Erysipelotrichales;Erysipelotrichaceae;  Holdemania;Holdemania_filiformis | 0.005 | 0.010 | 0.006 | 0.013 | 0.572 |
| OTU343 | Firmicutes;Negativicutes;Selenomonadales;Veillonellaceae;Megasphaera;  Megasphaera_micronuciformis | 0.002 | 0.010 | 0.001 | 0.002 | 0.044 |
| OTU344 | Firmicutes;Clostridia;Clostridiales;Lachnospiraceae;  Lachnospiracea_incertae_sedis;Eubacterium_uniforme | 0.003 | 0.012 | 0.016 | 0.058 | 0.009 |
| OTU345 | Firmicutes;Bacilli;Lactobacillales;Enterococcaceae;Enterococcus;  Enterococcus_saccharolyticus | 0.001 | 0.007 | 0.001 | 0.002 | 0.976 |
| OTU346 | Firmicutes;Erysipelotrichia;Erysipelotrichales;Erysipelotrichaceae;  Faecalicoccus;Faecalicoccus_pleomorphus | 0.005 | 0.008 | 0.004 | 0.011 | 0.262 |
| OTU347 | Bacteroidetes;Bacteroidia;Bacteroidales;Porphyromonadaceae | 0.000 | 0.000 | 0.001 | 0.005 | 0.354 |
| OTU348 | ActinoActinoActinomycetales;Actinomycetaceae;Mobiluncus;  Mobiluncus_curtisii | 0.001 | 0.007 | 0.000 | 0.000 | 0.310 |
| OTU349 | Bacteria | 0.000 | 0.000 | 0.001 | 0.005 | 0.983 |
| OTU35 | Firmicutes;Clostridia;Clostridiales | 0.192 | 1.081 | 0.001 | 0.004 | 0.889 |
| OTU350 | Firmicutes;Clostridia;Clostridiales;Clostridiales;Anaerococcus;  Anaerococcus_vaginalis | 0.004 | 0.022 | 0.000 | 0.000 | 0.034 |
| OTU351 | Firmicutes;Negativicutes;Selenomonadales;Veillonellaceae;Megasphaera;  Megasphaera_elsdenii | 0.001 | 0.002 | 0.007 | 0.035 | 0.990 |
| OTU352 | Firmicutes;Clostridia;Clostridiales | 0.000 | 0.000 | 0.002 | 0.013 | 0.354 |
| OTU353 | Firmicutes;Clostridia;Clostridiales | 0.001 | 0.002 | 0.006 | 0.014 | 0.292 |
| OTU354 | Bacteroidetes;Bacteroidia;Bacteroidales;Porphyromonadaceae | 0.000 | 0.000 | 0.002 | 0.010 | 0.354 |
| OTU355 | ProteoBetaproteoBurkholderiales;Sutterellaceae | 0.000 | 0.002 | 0.001 | 0.005 | 0.939 |
| OTU356 | Bacteroidetes;Bacteroidia;Bacteroidales;Porphyromonadaceae;  Butyricimonas | 0.000 | 0.000 | 0.001 | 0.004 | 0.180 |
| OTU357 | Firmicutes;Clostridia;Clostridiales | 0.000 | 0.001 | 0.001 | 0.005 | 0.983 |
| OTU358 | Firmicutes;Clostridia;Clostridiales | 0.000 | 0.000 | 0.001 | 0.006 | 0.354 |
| OTU359 | Bacteroidetes;Bacteroidia;Bacteroidales;Prevotellaceae;Prevotella;  Prevotella_copri | 0.040 | 0.180 | 0.000 | 0.001 | 0.133 |
| OTU36 | Bacteroidetes;Bacteroidia;Bacteroidales;Bacteroidaceae;Bacteroides;  Bacteroides_uniformis | 0.822 | 1.958 | 1.419 | 2.359 | 0.127 |
| OTU360 | ProteoGammaproteoPseudomonadales;Moraxellaceae;Acinetobacter;  Acinetobacter_johnsonii | 0.003 | 0.016 | 0.002 | 0.013 | 0.517 |
| OTU361 | Firmicutes;Negativicutes;Selenomonadales;Veillonellaceae;Megamonas;  Megamonas_funiformis | 0.002 | 0.005 | 0.005 | 0.012 | 0.786 |
| OTU362 | Firmicutes;Clostridia;Clostridiales;Ruminococcaceae;Clostridium_III | 0.000 | 0.000 | 0.001 | 0.004 | 0.354 |
| OTU363 | Bacteroidetes;Bacteroidia;Bacteroidales;Bacteroidaceae;Bacteroides;  Bacteroides_vulgatus | 0.000 | 0.001 | 0.001 | 0.003 | 0.624 |
| OTU364 | ProteoBetaproteoBurkholderiales;Comamonadaceae;Comamonas;  Comamonas_kerstersii | 0.000 | 0.002 | 0.001 | 0.005 | 0.710 |
| OTU365 | ActinoActinoActinomycetales;Actinomycetaceae;Actinomyces;  Actinomyces_odontolyticus | 0.021 | 0.035 | 0.009 | 0.010 | 0.945 |
| OTU366 | FusoFusobacteriia;Fusobacteriales;Leptotrichiaceae;Sneathia;  Sneathia_sanguinegens | 0.000 | 0.000 | 0.001 | 0.006 | 0.354 |
| OTU367 | Firmicutes;Clostridia;Clostridiales;Ruminococcaceae | 0.000 | 0.000 | 0.002 | 0.011 | 0.354 |
| OTU368 | Firmicutes;Clostridia;Clostridiales;Ruminococcaceae;Intestinimonas;  Intestinimonas_butyriciproducens | 0.001 | 0.002 | 0.005 | 0.011 | 0.385 |
| OTU369 | Firmicutes;Clostridia;Clostridiales;Ruminococcaceae;Gemmiger;  Gemmiger_formicilis | 0.002 | 0.010 | 0.030 | 0.150 | 0.004 |
| OTU37 | Firmicutes;Negativicutes;Selenomonadales;Acidaminococcaceae;  Phascolarctobacterium;Phascolarctobacterium_succinatutens | 0.188 | 0.836 | 0.234 | 1.044 | 0.552 |
| OTU370 | ProteoDeltaproteoDesulfovibrionales;Desulfovibrionaceae;Desulfovibrio | 0.001 | 0.004 | 0.001 | 0.002 | 0.293 |
| OTU371 | ProteoGammaproteoPasteurellales;Pasteurellaceae;Aggregatibacter;  Aggregatibacter_aphrophilus | 0.000 | 0.001 | 0.001 | 0.004 | 0.351 |
| OTU372 | Bacteroidetes;Bacteroidia;Bacteroidales;Prevotellaceae;Prevotella | 0.000 | 0.000 | 0.003 | 0.015 | 0.354 |
| OTU373 | ProteoBetaproteoBurkholderiales;Oxalobacteraceae;Oxalobacter;  Oxalobacter_formigenes | 0.000 | 0.001 | 0.002 | 0.007 | 0.705 |
| OTU374 | Bacteroidetes;Bacteroidia;Bacteroidales;Porphyromonadaceae;  Butyricimonas;Butyricimonas_paravirosa | 0.002 | 0.007 | 0.008 | 0.020 | 0.047 |
| OTU375 | Firmicutes;Negativicutes;Selenomonadales;Veillonellaceae;Megamonas;  Megamonas_funiformis | 0.004 | 0.011 | 0.029 | 0.091 | 0.292 |
| OTU376 | Firmicutes;Erysipelotrichia;Erysipelotrichales;Erysipelotrichaceae;  Clostridium_XVIII | 0.000 | 0.000 | 0.001 | 0.004 | 0.354 |
| OTU377 | Firmicutes;Clostridia;Clostridiales;Ruminococcaceae | 0.000 | 0.000 | 0.001 | 0.006 | 0.354 |
| OTU378 | Firmicutes;Clostridia;Clostridiales | 0.000 | 0.000 | 0.001 | 0.006 | 0.052 |
| OTU379 | Firmicutes;Clostridia;Clostridiales;Lachnospiraceae;Clostridium_XlVa | 0.001 | 0.003 | 0.004 | 0.012 | 0.156 |
| OTU38 | Firmicutes;Clostridia;Clostridiales;Lachnospiraceae;Roseburia;  Roseburia_inulinivorans | 0.975 | 1.462 | 1.692 | 2.062 | 0.130 |
| OTU380 | Firmicutes;Clostridia;Clostridiales;Lachnospiraceae | 0.007 | 0.007 | 0.019 | 0.058 | 0.776 |
| OTU381 | Firmicutes;Clostridia;Clostridiales | 0.001 | 0.004 | 0.018 | 0.063 | 0.062 |
| OTU382 | Bacteroidetes;Bacteroidia;Bacteroidales;Prevotellaceae;Prevotella;  Prevotella_copri | 0.000 | 0.000 | 0.034 | 0.182 | 0.096 |
| OTU383 | Firmicutes;Clostridia;Clostridiales;Lachnospiraceae | 0.011 | 0.018 | 0.018 | 0.035 | 0.259 |
| OTU384 | Firmicutes;Erysipelotrichia;Erysipelotrichales;Erysipelotrichaceae;  Faecalicoccus | 0.000 | 0.001 | 0.002 | 0.006 | 0.970 |
| OTU385 | Firmicutes;Clostridia;Clostridiales | 0.000 | 0.001 | 0.003 | 0.010 | 0.469 |
| OTU386 | Bacteroidetes;Bacteroidia;Bacteroidales;Prevotellaceae;Prevotella;  Prevotella_timonensis | 0.000 | 0.001 | 0.002 | 0.014 | 0.540 |
| OTU387 | ProteoGammaproteoEnterobacteriales;Enterobacteriaceae;Proteus | 0.002 | 0.012 | 0.000 | 0.000 | 0.142 |
| OTU388 | Firmicutes;Bacilli;Lactobacillales;Lactobacillaceae;Lactobacillus | 0.000 | 0.000 | 0.001 | 0.007 | 0.336 |
| OTU389 | ActinoActinoCoriobacteriales;Coriobacteriaceae | 0.001 | 0.005 | 0.001 | 0.004 | 0.518 |
| OTU39 | Firmicutes;Negativicutes;Selenomonadales;Acidaminococcaceae;  Acidaminococcus;Acidaminococcus_fermentans | 0.152 | 0.833 | 0.006 | 0.014 | 0.519 |
| OTU390 | CyanoCyanoUnclassified;Family_I;GpI | 0.000 | 0.000 | 0.000 | 0.003 | 0.354 |
| OTU391 | Bacteroidetes;Bacteroidia;Bacteroidales;Prevotellaceae;Prevotella | 0.011 | 0.059 | 0.000 | 0.003 | 0.949 |
| OTU392 | Firmicutes;Clostridia;Clostridiales;Clostridiales;Anaerococcus;  Anaerococcus_octavius | 0.001 | 0.007 | 0.000 | 0.000 | 0.310 |
| OTU393 | Firmicutes;Clostridia;Clostridiales;Lachnospiraceae;Oribacterium;  Oribacterium_sinus | 0.003 | 0.009 | 0.002 | 0.002 | 0.064 |
| OTU394 | ActinoActinoCoriobacteriales;Coriobacteriaceae;Slackia;Slackia_piriformis | 0.001 | 0.002 | 0.001 | 0.006 | 0.292 |
| OTU395 | ProteoBetaproteoBurkholderiales;Sutterellaceae;Parasutterella;  Parasutterella_excrementihominis | 0.370 | 0.771 | 0.365 | 0.985 | 0.762 |
| OTU396 | FusoFusobacteriia;Fusobacteriales;Fusobacteriaceae;Fusobacterium | 0.000 | 0.000 | 0.004 | 0.019 | 0.180 |
| OTU397 | ActinoActinoCoriobacteriales;Coriobacteriaceae;Gordonibacter | 0.000 | 0.000 | 0.001 | 0.004 | 0.052 |
| OTU398 | Firmicutes;Clostridia;Clostridiales;Peptoniphilaceae;Peptoniphilus;  Peptoniphilus_lacrimalis | 0.001 | 0.008 | 0.000 | 0.000 | 0.517 |
| OTU399 | Firmicutes;Clostridia;Clostridiales | 0.000 | 0.000 | 0.001 | 0.004 | 0.096 |
| OTU4 | Bacteroidetes;Bacteroidia;Bacteroidales;Bacteroidaceae;Bacteroides;  Bacteroides_vulgatus | 5.579 | 9.915 | 10.364 | 13.751 | 0.124 |
| OTU40 | Firmicutes;Clostridia;Clostridiales;Lachnospiraceae;Clostridium_XlVa | 0.752 | 1.190 | 1.043 | 2.017 | 0.807 |
| OTU400 | Bacteria | 0.001 | 0.004 | 0.000 | 0.000 | 0.949 |
| OTU401 | Firmicutes;Clostridia;Clostridiales | 0.000 | 0.000 | 0.001 | 0.006 | 0.195 |
| OTU402 | Firmicutes;Bacilli;Bacillales;Staphylococcaceae;Staphylococcus | 0.000 | 0.000 | 0.001 | 0.006 | 0.624 |
| OTU403 | Firmicutes;Clostridia;Clostridiales;Ruminococcaceae | 0.000 | 0.000 | 0.001 | 0.005 | 0.180 |
| OTU404 | Firmicutes;Clostridia;Clostridiales | 0.000 | 0.000 | 0.003 | 0.006 | 0.030 |
| OTU405 | Firmicutes;Clostridia;Clostridiales;Ruminococcaceae | 0.000 | 0.000 | 0.001 | 0.004 | 0.052 |
| OTU406 | Firmicutes;Clostridia;Clostridiales;Lachnospiraceae;Fusicatenibacter;  Fusicatenibacter_saccharivorans | 0.106 | 0.294 | 0.071 | 0.112 | 0.306 |
| OTU407 | Firmicutes;Negativicutes;Selenomonadales;Veillonellaceae;Mitsuokella | 0.013 | 0.071 | 0.002 | 0.009 | 0.517 |
| OTU408 | Firmicutes;Clostridia;Clostridiales;Peptococcaceae;Peptococcus;  Peptococcus_niger | 0.001 | 0.003 | 0.000 | 0.000 | 0.310 |
| OTU409 | Bacteroidetes;Bacteroidia;Bacteroidales;Prevotellaceae;Prevotella | 0.000 | 0.000 | 0.001 | 0.005 | 0.354 |
| OTU41 | Firmicutes;Erysipelotrichia;Erysipelotrichales;Erysipelotrichaceae;  Catenibacterium;Catenibacterium_mitsuokai | 0.126 | 0.697 | 0.144 | 0.738 | 0.362 |
| OTU410 | Firmicutes;Clostridia;Clostridiales;Ruminococcaceae | 0.000 | 0.002 | 0.003 | 0.006 | 0.096 |
| OTU411 | Firmicutes;Clostridia;Clostridiales;Lachnospiraceae;Anaerostipes | 0.038 | 0.078 | 0.061 | 0.169 | 0.796 |
| OTU412 | FusoFusobacteriia;Fusobacteriales;Fusobacteriaceae;Fusobacterium;  Fusobacterium_nucleatum | 0.002 | 0.006 | 0.001 | 0.004 | 0.196 |
| OTU413 | Firmicutes;Clostridia;Clostridiales;Lachnospiraceae;Pseudobutyrivibrio;  Pseudobutyrivibrio_ruminis | 0.005 | 0.017 | 0.014 | 0.028 | 0.048 |
| OTU414 | ActinoActinoCoriobacteriales;Coriobacteriaceae;Eggerthella;  Eggerthella_lenta | 0.002 | 0.006 | 0.001 | 0.001 | 0.649 |
| OTU415 | Bacteria | 0.000 | 0.000 | 0.001 | 0.005 | 0.180 |
| OTU416 | Firmicutes;Clostridia;Clostridiales;Lachnospiraceae;  Lachnospiracea_incertae_sedis;Ruminococcus_gnavus | 0.062 | 0.133 | 0.073 | 0.215 | 0.006 |
| OTU417 | FusoFusobacteriia;Fusobacteriales;Fusobacteriaceae;Fusobacterium | 0.002 | 0.005 | 0.955 | 5.645 | 0.162 |
| OTU418 | ProteoEpsilonproteoCampylobacterales;Campylobacteraceae;  Campylobacter;Campylobacter_concisus | 0.000 | 0.001 | 0.001 | 0.007 | 0.367 |
| OTU419 | Firmicutes;Clostridia;Clostridiales | 0.000 | 0.001 | 0.003 | 0.006 | 0.014 |
| OTU42 | Bacteroidetes;Bacteroidia;Bacteroidales;Porphyromonadaceae | 0.074 | 0.190 | 0.348 | 1.073 | 0.356 |
| OTU420 | Firmicutes;Clostridia;Clostridiales | 0.000 | 0.000 | 0.001 | 0.004 | 0.336 |
| OTU421 | Firmicutes;Negativicutes;Selenomonadales;Veillonellaceae;Megamonas | 0.019 | 0.047 | 0.010 | 0.034 | 0.597 |
| OTU422 | Firmicutes;Negativicutes;Selenomonadales;Veillonellaceae;Megasphaera | 0.000 | 0.002 | 0.014 | 0.067 | 0.037 |
| OTU423 | Bacteroidetes;Bacteroidia;Bacteroidales;Bacteroidaceae;Bacteroides | 0.088 | 0.421 | 0.043 | 0.161 | 0.932 |
| OTU424 | Firmicutes;Clostridia;Clostridiales;Ruminococcaceae;Clostridium_IV;  Clostridium_leptum | 0.000 | 0.001 | 0.001 | 0.003 | 0.540 |
| OTU425 | Firmicutes;Bacilli;Lactobacillales;Lactobacillaceae;Lactobacillus | 0.001 | 0.006 | 0.001 | 0.006 | 0.628 |
| OTU426 | ProteoBetaproteoBurkholderiales;Oxalobacteraceae;Oxalobacter;  Oxalobacter_formigenes | 0.001 | 0.003 | 0.001 | 0.005 | 0.808 |
| OTU427 | Firmicutes;Clostridia;Clostridiales;Ruminococcaceae;Clostridium_III | 0.000 | 0.000 | 0.002 | 0.011 | 0.180 |
| OTU428 | Firmicutes;Clostridia;Clostridiales;Lachnospiraceae | 0.331 | 1.033 | 0.316 | 0.796 | 0.309 |
| OTU429 | Firmicutes;Bacilli;Lactobacillales;Lactobacillaceae;Pediococcus;  Pediococcus_parvulus | 0.001 | 0.005 | 0.002 | 0.009 | 0.983 |
| OTU43 | Firmicutes;Clostridia;Clostridiales;Ruminococcaceae;Ruminococcus;  Ruminococcus_bromii | 0.220 | 0.730 | 0.602 | 1.397 | 0.013 |
| OTU430 | ActinoActinoCoriobacteriales;Coriobacteriaceae;Atopobium;  Atopobium_rimae | 0.003 | 0.005 | 0.001 | 0.002 | 0.669 |
| OTU431 | Bacteroidetes;Bacteroidia;Bacteroidales;Prevotellaceae;Prevotella | 0.003 | 0.009 | 0.000 | 0.000 | 0.069 |
| OTU432 | Firmicutes;Clostridia;Clostridiales | 0.000 | 0.000 | 0.002 | 0.005 | 0.055 |
| OTU433 | Bacteroidetes;Bacteroidia;Bacteroidales;Prevotellaceae;Prevotella;  Prevotella_copri | 0.012 | 0.061 | 0.005 | 0.030 | 0.355 |
| OTU434 | Firmicutes;Clostridia;Clostridiales | 0.000 | 0.001 | 0.001 | 0.003 | 0.410 |
| OTU435 | Firmicutes;Negativicutes;Selenomonadales;Veillonellaceae;Dialister;  Dialister_succinatiphilus | 0.002 | 0.008 | 0.012 | 0.066 | 0.929 |
| OTU436 | Firmicutes;Bacilli;Lactobacillales;Streptococcaceae;Streptococcus;  Streptococcus_anginosus | 0.001 | 0.002 | 0.001 | 0.004 | 0.153 |
| OTU437 | ProteoDeltaproteoDesulfovibrionales;Desulfovibrionaceae;Desulfovibrio | 0.000 | 0.000 | 0.001 | 0.006 | 0.351 |
| OTU438 | ActinoActinoActinomycetales;Micrococcaceae;Rothia;Rothia_aeria | 0.001 | 0.003 | 0.000 | 0.001 | 0.909 |
| OTU439 | Firmicutes;Clostridia;Clostridiales;Peptoniphilaceae;Peptoniphilus | 0.001 | 0.005 | 0.000 | 0.000 | 0.310 |
| OTU44 | Firmicutes;Negativicutes;Selenomonadales;Veillonellaceae;Dialister;  Dialister_succinatiphilus | 0.003 | 0.016 | 0.100 | 0.593 | 0.540 |
| OTU440 | Candidatus_SacchariUnclassified;Unclassified;Unclassified;  SacchariTM7_phylum | 0.004 | 0.008 | 0.002 | 0.004 | 0.847 |
| OTU441 | Bacteroidetes;Bacteroidia;Bacteroidales;Bacteroidaceae;Bacteroides | 0.001 | 0.003 | 0.000 | 0.001 | 0.649 |
| OTU442 | Bacteroidetes;Bacteroidia;Bacteroidales;Bacteroidaceae;Bacteroides | 0.001 | 0.006 | 0.000 | 0.000 | 0.310 |
| OTU443 | Bacteroidetes;Bacteroidia;Bacteroidales | 0.005 | 0.027 | 0.000 | 0.002 | 0.329 |
| OTU444 | Firmicutes;Negativicutes;Selenomonadales;Veillonellaceae;Megamonas | 0.005 | 0.014 | 0.000 | 0.001 | 0.135 |
| OTU445 | ActinoActinoBifidobacteriales;Bifidobacteriaceae;Bifidobacterium;  Bifidobacterium_adolescentis | 0.096 | 0.396 | 0.106 | 0.352 | 0.963 |
| OTU446 | Firmicutes;Clostridia;Clostridiales;Ruminococcaceae | 0.000 | 0.000 | 0.001 | 0.002 | 0.052 |
| OTU447 | Firmicutes;Clostridia;Clostridiales | 0.000 | 0.001 | 0.002 | 0.007 | 0.425 |
| OTU448 | Firmicutes;Clostridia;Clostridiales;Ruminococcaceae;Anaerotruncus;  Anaerotruncus_colihominis | 0.000 | 0.001 | 0.001 | 0.003 | 0.731 |
| OTU449 | Bacteria | 0.001 | 0.003 | 0.000 | 0.001 | 0.578 |
| OTU45 | Bacteroidetes;Bacteroidia;Bacteroidales;Prevotellaceae;Prevotella;  Prevotella_copri | 0.731 | 2.334 | 0.088 | 0.488 | 0.914 |
| OTU450 | Firmicutes;Negativicutes;Selenomonadales;Veillonellaceae;Megasphaera;  Megasphaera_indica | 0.004 | 0.022 | 0.000 | 0.000 | 0.949 |
| OTU451 | Firmicutes;Clostridia;Clostridiales;Lachnospiraceae | 0.672 | 1.194 | 0.805 | 1.068 | 0.181 |
| OTU452 | Firmicutes;Negativicutes;Selenomonadales;Veillonellaceae;Megamonas | 0.001 | 0.003 | 0.006 | 0.030 | 0.990 |
| OTU453 | ActinoActinoActinomycetales;Dietziaceae;Dietzia | 0.001 | 0.003 | 0.000 | 0.000 | 0.310 |
| OTU454 | Bacteroidetes;Bacteroidia;Bacteroidales;Prevotellaceae;Prevotella;  Prevotella_copri | 0.004 | 0.010 | 0.002 | 0.005 | 0.304 |
| OTU455 | Firmicutes;Negativicutes;Selenomonadales;Veillonellaceae;Dialister;  Dialister_succinatiphilus | 0.006 | 0.030 | 0.003 | 0.012 | 0.502 |
| OTU456 | Bacteroidetes;Bacteroidia;Bacteroidales;Porphyromonadaceae;  Parabacteroides;Parabacteroides_merdae | 0.000 | 0.000 | 0.001 | 0.007 | 0.983 |
| OTU457 | Firmicutes;Clostridia;Clostridiales;Lachnospiraceae;Clostridium_XlVa;  Clostridium_xylanolyticum | 0.001 | 0.002 | 0.005 | 0.010 | 0.048 |
| OTU458 | Synergistetes;Synergistia;Synergistales;Synergistaceae;Cloacibacillus;  Cloacibacillus_porcorum | 0.000 | 0.000 | 0.002 | 0.011 | 0.029 |
| OTU459 | Firmicutes;Clostridia;Clostridiales;Lachnospiraceae;Blautia | 0.046 | 0.066 | 0.058 | 0.083 | 0.127 |
| OTU46 | Bacteroidetes;Bacteroidia;Bacteroidales;Prevotellaceae;Alloprevotella;  Alloprevotella_rava | 0.382 | 1.875 | 0.000 | 0.002 | 0.243 |
| OTU460 | Firmicutes;Clostridia;Clostridiales | 0.000 | 0.000 | 0.002 | 0.006 | 0.096 |
| OTU461 | Bacteroidetes;Bacteroidia;Bacteroidales;Prevotellaceae;Prevotella;  Prevotella_copri | 0.550 | 2.086 | 0.141 | 0.431 | 0.245 |
| OTU462 | Bacteroidetes;Bacteroidia;Bacteroidales;Bacteroidaceae;Bacteroides;  Bacteroides_coprophilus | 0.020 | 0.113 | 0.000 | 0.000 | 0.310 |
| OTU463 | ActinoActinoActinomycetales;Micrococcaceae;Arthrobacter | 0.001 | 0.003 | 0.000 | 0.000 | 0.310 |
| OTU464 | Firmicutes;Clostridia;Clostridiales;Lachnospiraceae | 0.056 | 0.071 | 0.217 | 0.383 | 0.095 |
| OTU465 | Firmicutes;Clostridia;Clostridiales;Lachnospiraceae | 0.001 | 0.005 | 0.002 | 0.006 | 0.759 |
| OTU466 | Firmicutes;Clostridia;Clostridiales;Lachnospiraceae;Cellulosilyticum;  Cellulosilyticum_ruminicola | 0.001 | 0.004 | 0.000 | 0.000 | 0.310 |
| OTU467 | Firmicutes;Clostridia;Clostridiales | 0.000 | 0.001 | 0.002 | 0.004 | 0.157 |
| OTU468 | Firmicutes;Erysipelotrichia;Erysipelotrichales;Erysipelotrichaceae | 0.000 | 0.000 | 0.000 | 0.002 | 0.354 |
| OTU469 | Firmicutes;Clostridia;Clostridiales | 0.000 | 0.000 | 0.002 | 0.008 | 0.096 |
| OTU47 | Firmicutes;Clostridia;Clostridiales;Ruminococcaceae | 0.026 | 0.067 | 0.246 | 0.653 | 0.028 |
| OTU470 | Bacteroidetes;Bacteroidia;Bacteroidales;Prevotellaceae;Prevotella;  Prevotella_copri | 0.015 | 0.084 | 0.000 | 0.000 | 0.142 |
| OTU471 | Bacteroidetes;Bacteroidia;Bacteroidales;Bacteroidaceae;Bacteroides;  Bacteroides_plebeius | 0.000 | 0.001 | 0.002 | 0.012 | 0.292 |
| OTU472 | Bacteroidetes;Bacteroidia;Bacteroidales;Bacteroidaceae;Bacteroides;  Bacteroides_clarus | 0.323 | 0.951 | 0.628 | 1.169 | 1.000 |
| OTU473 | Firmicutes;Clostridia;Clostridiales;Ruminococcaceae | 0.001 | 0.003 | 0.000 | 0.000 | 0.310 |
| OTU474 | Bacteroidetes;Bacteroidia;Bacteroidales;Bacteroidaceae;Bacteroides;  Bacteroides_coprocola | 0.003 | 0.017 | 0.003 | 0.016 | 0.601 |
| OTU475 | Firmicutes;Clostridia;Clostridiales;Clostridiales | 0.001 | 0.005 | 0.000 | 0.000 | 0.949 |
| OTU476 | Firmicutes;Clostridia;Clostridiales;Ruminococcaceae;Butyricicoccus;  Butyricicoccus_pullicaecorum | 0.000 | 0.000 | 0.001 | 0.003 | 0.052 |
| OTU477 | Firmicutes;Bacilli;Lactobacillales;Streptococcaceae;Streptococcus;  Streptococcus_infantarius | 0.005 | 0.025 | 0.001 | 0.003 | 0.891 |
| OTU478 | Firmicutes;Clostridia;Clostridiales;Lachnospiraceae;Clostridium_XlVb | 0.001 | 0.003 | 0.001 | 0.005 | 0.903 |
| OTU479 | Firmicutes;Negativicutes;Selenomonadales;Veillonellaceae;Dialister;  Dialister_succinatiphilus | 0.002 | 0.009 | 0.000 | 0.001 | 0.384 |
| OTU48 | Firmicutes;Negativicutes;Selenomonadales;Veillonellaceae;Veillonella;  Veillonella_dispar | 0.406 | 1.846 | 0.134 | 0.213 | 0.066 |
| OTU480 | Firmicutes;Clostridia;Clostridiales;Ruminococcaceae | 0.000 | 0.000 | 0.001 | 0.002 | 0.096 |
| OTU481 | Verrucomicrobia;Verrucomicrobiae;Verrucomicrobiales;  Verrucomicrobiaceae;Akkermansia;Akkermansia_muciniphila | 0.048 | 0.241 | 0.046 | 0.227 | 0.253 |
| OTU482 | ActinoActinoCoriobacteriales;Coriobacteriaceae;Gordonibacter;  Gordonibacter_pamelaeae | 0.001 | 0.002 | 0.000 | 0.001 | 0.889 |
| OTU483 | Bacteroidetes;Bacteroidia;Bacteroidales;Porphyromonadaceae;  Parabacteroides;Parabacteroides_goldsteinii | 0.000 | 0.001 | 0.005 | 0.019 | 0.688 |
| OTU484 | Firmicutes;Clostridia;Clostridiales | 0.000 | 0.000 | 0.001 | 0.004 | 0.336 |
| OTU485 | Bacteroidetes;Bacteroidia;Bacteroidales;Bacteroidaceae;Bacteroides;  Bacteroides_acidifaciens | 0.001 | 0.002 | 0.003 | 0.018 | 0.628 |
| OTU486 | Bacteroidetes;Bacteroidia;Bacteroidales;Rikenellaceae;Alistipes;  Alistipes_indistinctus | 0.001 | 0.003 | 0.000 | 0.002 | 0.278 |
| OTU487 | ProteoEpsilonproteoCampylobacterales;Campylobacteraceae;  Campylobacter;Campylobacter_hominis | 0.000 | 0.002 | 0.000 | 0.000 | 0.142 |
| OTU488 | Bacteroidetes;Bacteroidia;Bacteroidales;Bacteroidaceae;Bacteroides | 0.023 | 0.133 | 0.000 | 0.000 | 0.949 |
| OTU489 | Firmicutes;Negativicutes;Selenomonadales;Veillonellaceae;Megamonas;  Megamonas_funiformis | 0.005 | 0.025 | 0.025 | 0.128 | 0.929 |
| OTU49 | ProteoBetaproteoBurkholderiales;Sutterellaceae;Sutterella;  Sutterella_wadsworthensis | 0.209 | 0.560 | 0.113 | 0.312 | 0.662 |
| OTU490 | Bacteroidetes;Bacteroidia;Bacteroidales;Porphyromonadaceae | 0.001 | 0.006 | 0.000 | 0.000 | 0.310 |
| OTU491 | Bacteria | 0.000 | 0.000 | 0.000 | 0.002 | 0.180 |
| OTU492 | Bacteroidetes;Bacteroidia;Bacteroidales;Porphyromonadaceae;  Butyricimonas | 0.001 | 0.002 | 0.001 | 0.005 | 0.483 |
| OTU493 | Firmicutes;Clostridia;Clostridiales;Lachnospiraceae | 0.012 | 0.026 | 0.009 | 0.015 | 0.122 |
| OTU494 | Firmicutes;Clostridia;Clostridiales;Ruminococcaceae;Clostridium_IV;  Clostridium_methylpentosum | 0.001 | 0.003 | 0.001 | 0.003 | 0.315 |
| OTU495 | Bacteroidetes;Bacteroidia;Bacteroidales;Prevotellaceae;Prevotella;  Prevotella_copri | 0.011 | 0.040 | 0.011 | 0.034 | 0.686 |
| OTU496 | Bacteroidetes;Bacteroidia;Bacteroidales;Bacteroidaceae;Bacteroides | 0.000 | 0.002 | 0.006 | 0.016 | 0.002 |
| OTU497 | Firmicutes;Clostridia;Clostridiales | 0.000 | 0.000 | 0.000 | 0.003 | 0.354 |
| OTU498 | Firmicutes;Clostridia;Clostridiales;Ruminococcaceae | 0.000 | 0.000 | 0.000 | 0.003 | 0.354 |
| OTU499 | Bacteroidetes;Bacteroidia;Bacteroidales;Porphyromonadaceae;Barnesiella;Barnesiella_intestinihominis | 0.000 | 0.002 | 0.000 | 0.000 | 0.310 |
| OTU5 | Firmicutes;Clostridia;Clostridiales;Lachnospiraceae | 3.765 | 9.449 | 4.228 | 5.679 | 0.026 |
| OTU50 | Bacteroidetes;Bacteroidia;Bacteroidales;Prevotellaceae;Prevotella | 0.000 | 0.001 | 0.251 | 1.480 | 0.205 |
| OTU500 | Bacteroidetes;Bacteroidia;Bacteroidales;Prevotellaceae;Prevotella;  Prevotella_copri | 0.011 | 0.059 | 0.000 | 0.000 | 0.069 |
| OTU501 | Firmicutes;Clostridia;Clostridiales;Lachnospiraceae;Blautia;Blautia_luti | 0.107 | 0.122 | 0.094 | 0.117 | 0.915 |
| OTU502 | Firmicutes;Clostridia;Clostridiales;Lachnospiraceae;Anaerostipes;  Anaerostipes_caccae | 0.000 | 0.002 | 0.001 | 0.003 | 0.455 |
| OTU503 | Bacteria | 0.001 | 0.003 | 0.000 | 0.000 | 0.310 |
| OTU504 | ProteoAlphaproteoRhizobiales;Bradyrhizobiaceae;Bradyrhizobium | 0.000 | 0.002 | 0.000 | 0.000 | 0.310 |
| OTU505 | Bacteroidetes;Bacteroidia;Bacteroidales;Prevotellaceae;Prevotella;  Prevotella_copri | 0.004 | 0.010 | 0.002 | 0.004 | 0.389 |
| OTU506 | Bacteroidetes;Bacteroidia;Bacteroidales;Porphyromonadaceae;  Butyricimonas | 0.000 | 0.000 | 0.000 | 0.003 | 0.354 |
| OTU507 | Firmicutes;Clostridia;Clostridiales;Ruminococcaceae;Oscillibacter | 0.000 | 0.000 | 0.002 | 0.006 | 0.052 |
| OTU508 | Firmicutes;Clostridia;Clostridiales;Lachnospiraceae;Roseburia;  Roseburia_inulinivorans | 0.020 | 0.046 | 0.060 | 0.082 | 0.025 |
| OTU509 | Firmicutes;Clostridia;Clostridiales;Ruminococcaceae | 0.000 | 0.000 | 0.001 | 0.003 | 0.354 |
| OTU51 | Firmicutes;Clostridia;Clostridiales;Eubacteriaceae;Eubacterium;  Eubacterium_coprostanoligenes | 0.026 | 0.092 | 0.324 | 0.934 | 0.026 |
| OTU510 | Firmicutes;Clostridia;Clostridiales;Ruminococcaceae | 0.001 | 0.002 | 0.001 | 0.004 | 0.486 |
| OTU511 | Firmicutes;Bacilli;Bacillales;Bacillales;Gemella;Gemella_sanguinis | 0.002 | 0.006 | 0.002 | 0.002 | 0.071 |
| OTU512 | Firmicutes;Bacilli;Lactobacillales;Leuconostocaceae;Leuconostoc;  Leuconostoc_citreum | 0.001 | 0.002 | 0.000 | 0.002 | 0.869 |
| OTU513 | ProteoDeltaproteoDesulfovibrionales;Desulfovibrionaceae;Desulfovibrio | 0.001 | 0.003 | 0.000 | 0.002 | 0.949 |
| OTU514 | Firmicutes;Bacilli;Lactobacillales;Lactobacillaceae;Pediococcus | 0.000 | 0.001 | 0.001 | 0.004 | 0.624 |
| OTU515 | Firmicutes;Clostridia;Clostridiales;Lachnospiraceae | 0.000 | 0.000 | 0.008 | 0.045 | 0.096 |
| OTU516 | Firmicutes;Clostridia;Clostridiales;Ruminococcaceae;Clostridium_IV;  Clostridium_leptum | 0.001 | 0.001 | 0.001 | 0.002 | 0.944 |
| OTU517 | Firmicutes;Bacilli;Bacillales;Bacillales;Gemella;Gemella_asaccharolytica | 0.000 | 0.000 | 0.000 | 0.002 | 0.354 |
| OTU518 | Firmicutes;Negativicutes;Selenomonadales;Veillonellaceae;Megamonas;  Megamonas_funiformis | 0.001 | 0.007 | 0.000 | 0.001 | 0.649 |
| OTU519 | ProteoGammaproteoEnterobacteriales;Enterobacteriaceae;Unclassified;  Obesumbacterium_proteus | 0.002 | 0.006 | 0.001 | 0.004 | 0.329 |
| OTU52 | Firmicutes;Clostridia;Clostridiales;Lachnospiraceae;Clostridium_XlVb;  Clostridium_lactatifermentans | 0.029 | 0.046 | 0.144 | 0.491 | 0.438 |
| OTU520 | Bacteroidetes;Bacteroidia;Bacteroidales;Prevotellaceae;Prevotella;  Prevotella_disiens | 0.001 | 0.004 | 0.000 | 0.000 | 0.142 |
| OTU521 | Bacteroidetes;Bacteroidia;Bacteroidales;Prevotellaceae;Prevotella;  Prevotella_multisaccharivorax | 0.000 | 0.000 | 0.000 | 0.001 | 0.354 |
| OTU522 | Firmicutes;Clostridia;Clostridiales;Lachnospiraceae | 0.011 | 0.022 | 0.046 | 0.078 | 0.003 |
| OTU523 | Firmicutes | 0.000 | 0.000 | 0.000 | 0.002 | 0.354 |
| OTU524 | ActinoActinoCoriobacteriales;Coriobacteriaceae;Slackia;  Slackia_isoflavoniconvertens | 0.000 | 0.002 | 0.000 | 0.001 | 0.517 |
| OTU525 | Firmicutes;Clostridia;Clostridiales;Clostridiales;Anaerococcus;  Anaerococcus_octavius | 0.001 | 0.003 | 0.000 | 0.000 | 0.310 |
| OTU526 | Bacteroidetes;Bacteroidia;Bacteroidales;Prevotellaceae;Paraprevotella;  Paraprevotella_xylaniphila | 0.000 | 0.002 | 0.000 | 0.001 | 0.949 |
| OTU527 | Firmicutes;Negativicutes;Selenomonadales;Veillonellaceae;Megamonas | 0.012 | 0.041 | 0.001 | 0.003 | 0.121 |
| OTU528 | ProteoDeltaproteoDesulfovibrionales;Desulfovibrionaceae;Desulfovibrio;  Desulfovibrio_piger | 0.000 | 0.000 | 0.001 | 0.003 | 0.354 |
| OTU529 | Firmicutes;Erysipelotrichia;Erysipelotrichales;Erysipelotrichaceae | 0.000 | 0.000 | 0.000 | 0.000 | 0.310 |
| OTU53 | Bacteroidetes;Bacteroidia;Bacteroidales;Bacteroidaceae;Bacteroides;  Bacteroides_coprocola | 0.101 | 0.569 | 0.000 | 0.000 | 0.310 |
| OTU530 | Firmicutes;Clostridia;Clostridiales;Ruminococcaceae;Sporobacter;  Sporobacter_termitidis | 0.000 | 0.000 | 0.001 | 0.002 | 0.096 |
| OTU531 | Firmicutes;Clostridia;Clostridiales | 0.000 | 0.002 | 0.000 | 0.000 | 0.310 |
| OTU532 | Firmicutes;Clostridia;Clostridiales;Ruminococcaceae;Ruminococcus;  Ruminococcus_bromii | 0.015 | 0.060 | 0.157 | 0.683 | 0.054 |
| OTU533 | Firmicutes;Clostridia;Clostridiales;Ruminococcaceae | 0.000 | 0.001 | 0.001 | 0.003 | 0.336 |
| OTU534 | Bacteroidetes;Bacteroidia;Bacteroidales;Prevotellaceae;Prevotella;  Prevotella_stercorea | 0.008 | 0.043 | 0.000 | 0.000 | 0.310 |
| OTU535 | Firmicutes;Clostridia;Clostridiales;Lachnospiraceae | 0.006 | 0.017 | 0.028 | 0.060 | 0.002 |
| OTU536 | Firmicutes;Negativicutes;Selenomonadales;Veillonellaceae;Megamonas;  Megamonas_funiformis | 0.001 | 0.005 | 0.008 | 0.036 | 0.759 |
| OTU537 | Firmicutes;Clostridia;Clostridiales;Ruminococcaceae;Clostridium_IV | 0.000 | 0.000 | 0.001 | 0.002 | 0.180 |
| OTU538 | Bacteroidetes;Bacteroidia;Bacteroidales;Bacteroidaceae;Bacteroides | 0.003 | 0.019 | 0.000 | 0.000 | 0.310 |
| OTU539 | ProteoBetaproteoBurkholderiales;Comamonadaceae;Curvibacter;  Curvibacter_lanceolatus | 0.001 | 0.005 | 0.000 | 0.000 | 0.495 |
| OTU54 | ProteoGammaproteoPasteurellales;Pasteurellaceae;Haemophilus;  Haemophilus_parainfluenzae | 0.234 | 1.040 | 0.418 | 1.007 | 0.000 |
| OTU540 | Firmicutes;Clostridia;Clostridiales;Lachnospiraceae;Blautia;  Blautia_schinkii | 0.023 | 0.089 | 0.001 | 0.002 | 0.041 |
| OTU541 | Bacteroidetes;Bacteroidia;Bacteroidales;Prevotellaceae;Prevotella;  Prevotella_copri | 0.032 | 0.140 | 0.000 | 0.000 | 0.265 |
| OTU542 | ProteoAlphaproteoSphingomonadales;Sphingomonadaceae;Sphingomonas;Sphingomonas_faeni | 0.000 | 0.001 | 0.000 | 0.000 | 0.310 |
| OTU543 | Firmicutes;Clostridia;Clostridiales;Ruminococcaceae;Faecalibacterium;  Faecalibacterium_prausnitzii | 0.007 | 0.031 | 0.004 | 0.011 | 0.652 |
| OTU544 | Bacteroidetes;Bacteroidia;Bacteroidales;Bacteroidaceae;Bacteroides | 0.003 | 0.009 | 0.006 | 0.019 | 0.566 |
| OTU545 | Firmicutes;Clostridia;Clostridiales;Ruminococcaceae;Ruminococcus;  Ruminococcus_albus | 0.000 | 0.002 | 0.000 | 0.000 | 0.310 |
| OTU546 | Bacteroidetes;Bacteroidia;Bacteroidales;Bacteroidaceae;Bacteroides | 0.001 | 0.005 | 0.001 | 0.001 | 0.714 |
| OTU547 | Firmicutes;Negativicutes;Selenomonadales;Veillonellaceae;Megamonas;  Megamonas_funiformis | 0.000 | 0.002 | 0.001 | 0.004 | 0.970 |
| OTU548 | ProteoGammaproteoEnterobacteriales;Enterobacteriaceae;Klebsiella;  Klebsiella_pneumoniae | 0.030 | 0.104 | 0.018 | 0.052 | 0.441 |
| OTU549 | Firmicutes;Negativicutes;Selenomonadales;Acidaminococcaceae;  Phascolarctobacterium;Phascolarctobacterium_succinatutens | 0.039 | 0.120 | 0.396 | 1.042 | 0.093 |
| OTU55 | Bacteroidetes;Bacteroidia;Bacteroidales;Prevotellaceae;Paraprevotella;  Paraprevotella_clara | 0.035 | 0.067 | 0.160 | 0.447 | 0.916 |
| OTU550 | Firmicutes;Bacilli;Lactobacillales;Lactobacillaceae;Lactobacillus;  Lactobacillus_zymae | 0.000 | 0.001 | 0.000 | 0.000 | 0.142 |
| OTU551 | Firmicutes;Clostridia;Clostridiales;Eubacteriaceae;Eubacterium;  Eubacterium_sulci | 0.001 | 0.002 | 0.001 | 0.002 | 0.800 |
| OTU552 | Firmicutes;Bacilli;Lactobacillales;Lactobacillaceae;Lactobacillus;  Lactobacillus_fermentum | 0.000 | 0.000 | 0.001 | 0.003 | 0.052 |
| OTU553 | Bacteroidetes;Bacteroidia;Bacteroidales;Prevotellaceae;Prevotella;  Prevotella_stercorea | 0.006 | 0.033 | 0.000 | 0.000 | 0.142 |
| OTU554 | Firmicutes;Clostridia;Clostridiales | 0.000 | 0.000 | 0.003 | 0.013 | 0.029 |
| OTU555 | Firmicutes;Clostridia;Clostridiales;Lachnospiraceae;Roseburia;  Roseburia_inulinivorans | 0.049 | 0.106 | 0.056 | 0.064 | 0.147 |
| OTU556 | ProteoAlphaproteoRhodospirillales;Rhodospirillaceae | 0.000 | 0.001 | 0.000 | 0.000 | 0.310 |
| OTU557 | Firmicutes;Clostridia;Clostridiales | 0.000 | 0.000 | 0.001 | 0.003 | 0.354 |
| OTU558 | Firmicutes;Erysipelotrichia;Erysipelotrichales;Erysipelotrichaceae | 0.000 | 0.000 | 0.000 | 0.002 | 0.354 |
| OTU559 | Firmicutes;Clostridia;Clostridiales;Ruminococcaceae | 0.000 | 0.001 | 0.004 | 0.022 | 0.777 |
| OTU56 | Firmicutes;Clostridia;Clostridiales | 0.001 | 0.003 | 0.260 | 0.746 | 0.012 |
| OTU560 | Firmicutes;Negativicutes;Selenomonadales;Veillonellaceae;Mitsuokella;  Mitsuokella_multacida | 0.000 | 0.001 | 0.001 | 0.002 | 0.751 |
| OTU561 | Firmicutes;Clostridia;Clostridiales;Lachnospiraceae | 0.003 | 0.007 | 0.016 | 0.024 | 0.023 |
| OTU562 | Firmicutes;Clostridia;Clostridiales;Ruminococcaceae;Clostridium_IV | 0.001 | 0.003 | 0.000 | 0.001 | 0.066 |
| OTU563 | Firmicutes;Clostridia;Clostridiales;Lachnospiraceae | 0.015 | 0.067 | 0.034 | 0.102 | 0.274 |
| OTU564 | ProteoBetaproteoBurkholderiales;Sutterellaceae;Parasutterella;  Parasutterella_secunda | 0.000 | 0.000 | 0.000 | 0.003 | 0.354 |
| OTU565 | Firmicutes;Clostridia;Clostridiales;Ruminococcaceae | 0.000 | 0.000 | 0.001 | 0.004 | 0.180 |
| OTU566 | Firmicutes;Clostridia;Clostridiales | 0.000 | 0.000 | 0.000 | 0.002 | 0.096 |
| OTU567 | Bacteroidetes;Bacteroidia;Bacteroidales;Prevotellaceae;Prevotella;  Prevotella_copri | 0.084 | 0.224 | 0.066 | 0.253 | 0.953 |
| OTU568 | FusoFusobacteriia;Fusobacteriales;Fusobacteriaceae;Fusobacterium;  Fusobacterium_mortiferum | 0.001 | 0.004 | 0.000 | 0.000 | 0.142 |
| OTU569 | Firmicutes;Clostridia;Clostridiales;Clostridiales | 0.000 | 0.001 | 0.000 | 0.000 | 0.949 |
| OTU57 | Firmicutes;Negativicutes;Selenomonadales;Veillonellaceae;Dialister;  Dialister_succinatiphilus | 0.000 | 0.000 | 0.084 | 0.497 | 0.599 |
| OTU570 | Bacteroidetes;Bacteroidia;Bacteroidales;Porphyromonadaceae;  Butyricimonas | 0.000 | 0.000 | 0.000 | 0.002 | 0.354 |
| OTU571 | Tenericutes;Mollicutes;Acholeplasmatales;Acholeplasmataceae;  Acholeplasma;Acholeplasma_parvum | 0.000 | 0.000 | 0.000 | 0.002 | 0.180 |
| OTU572 | Firmicutes;Negativicutes;Selenomonadales;Veillonellaceae;Megamonas;  Megamonas_rupellensis | 0.001 | 0.005 | 0.000 | 0.000 | 0.008 |
| OTU573 | Firmicutes;Clostridia;Clostridiales | 0.000 | 0.000 | 0.000 | 0.002 | 0.180 |
| OTU574 | ProteoBetaproteoMethylophilales;Methylophilaceae;Methylophilus;  Methylophilus_leisingeri | 0.000 | 0.003 | 0.000 | 0.000 | 0.310 |
| OTU575 | Firmicutes;Negativicutes;Selenomonadales;Veillonellaceae;Megasphaera;  Megasphaera_indica | 0.001 | 0.003 | 0.000 | 0.001 | 0.949 |
| OTU576 | Firmicutes;Clostridia;Clostridiales;Lachnospiraceae | 0.000 | 0.000 | 0.025 | 0.075 | 0.008 |
| OTU577 | Firmicutes;Clostridia;Clostridiales | 0.000 | 0.000 | 0.000 | 0.002 | 0.354 |
| OTU578 | Firmicutes;Negativicutes;Selenomonadales;Veillonellaceae;Dialister;  Dialister_micraerophilus | 0.000 | 0.000 | 0.000 | 0.002 | 0.354 |
| OTU579 | ProteoGammaproteoEnterobacteriales;Enterobacteriaceae;Plesiomonas;  Plesiomonas_shigelloides | 0.000 | 0.000 | 0.001 | 0.006 | 0.983 |
| OTU58 | Bacteroidetes;Bacteroidia;Bacteroidales;Bacteroidaceae;Bacteroides;  Bacteroides_fragilis | 0.260 | 0.569 | 0.321 | 0.643 | 0.534 |
| OTU580 | Firmicutes;Clostridia;Clostridiales;Lachnospiraceae | 0.001 | 0.002 | 0.004 | 0.008 | 0.004 |
| OTU581 | ActinoActinoActinomycetales;Micrococcaceae;Nesterenkonia;  Nesterenkonia_sandarakina | 0.000 | 0.002 | 0.000 | 0.000 | 0.310 |
| OTU582 | ProteoBetaproteoBurkholderiales;Sutterellaceae;Parasutterella;  Parasutterella_excrementihominis | 0.015 | 0.035 | 0.014 | 0.032 | 0.730 |
| OTU583 | Firmicutes;Clostridia;Clostridiales;Lachnospiraceae;Lachnoanaerobaculum;Lachnoanaerobaculum_umeaense | 0.001 | 0.002 | 0.001 | 0.001 | 0.624 |
| OTU584 | Firmicutes;Erysipelotrichia;Erysipelotrichales;Erysipelotrichaceae;  Solobacterium;Solobacterium_moorei | 0.001 | 0.003 | 0.001 | 0.002 | 0.458 |
| OTU585 | Firmicutes;Negativicutes;Selenomonadales;Veillonellaceae;Megamonas;  Megamonas_rupellensis | 0.001 | 0.003 | 0.000 | 0.001 | 0.502 |
| OTU586 | Firmicutes;Clostridia;Clostridiales;Ruminococcaceae;Clostridium_IV | 0.000 | 0.000 | 0.000 | 0.002 | 0.180 |
| OTU587 | Bacteroidetes;Bacteroidia;Bacteroidales;Porphyromonadaceae;Barnesiella;Barnesiella_intestinihominis | 0.000 | 0.000 | 0.000 | 0.002 | 0.354 |
| OTU588 | Bacteroidetes;Bacteroidia;Bacteroidales;Bacteroidaceae;Bacteroides;  Bacteroides_coprocola | 0.279 | 1.078 | 0.933 | 3.182 | 0.450 |
| OTU589 | Firmicutes;Clostridia;Clostridiales;Clostridiales;Murdochiella;  Murdochiella_asaccharolytica | 0.001 | 0.003 | 0.000 | 0.000 | 0.310 |
| OTU59 | Firmicutes;Bacilli;Lactobacillales;Streptococcaceae;Streptococcus;  Streptococcus_salivarius | 0.114 | 0.459 | 0.153 | 0.513 | 0.317 |
| OTU590 | Bacteroidetes;Bacteroidia;Bacteroidales;Bacteroidaceae;Bacteroides;  Bacteroides_dorei | 0.002 | 0.004 | 0.004 | 0.005 | 0.061 |
| OTU591 | Firmicutes;Negativicutes;Selenomonadales;Veillonellaceae;Megamonas;  Megamonas_funiformis | 0.002 | 0.007 | 0.000 | 0.001 | 0.526 |
| OTU592 | ActinoActinoCoriobacteriales;Coriobacteriaceae;Olsenella;  Olsenella_scatoligenes | 0.001 | 0.003 | 0.000 | 0.001 | 0.891 |
| OTU593 | Firmicutes;Clostridia;Clostridiales;Ruminococcaceae | 0.000 | 0.000 | 0.000 | 0.001 | 0.354 |
| OTU594 | Firmicutes;Clostridia;Clostridiales;Lachnospiraceae;Stomatobaculum;  Stomatobaculum_longum | 0.001 | 0.002 | 0.000 | 0.000 | 0.178 |
| OTU595 | ActinoActinoActinomycetales;Corynebacteriaceae;Corynebacterium | 0.001 | 0.003 | 0.000 | 0.000 | 0.517 |
| OTU596 | Firmicutes;Clostridia;Clostridiales;Lachnospiraceae;Clostridium_XlVb;  Clostridium_lactatifermentans | 0.006 | 0.009 | 0.015 | 0.021 | 0.151 |
| OTU597 | Bacteroidetes;Bacteroidia;Bacteroidales;Prevotellaceae;Prevotella;  Prevotella_copri | 0.000 | 0.000 | 0.005 | 0.028 | 0.983 |
| OTU598 | Synergistetes;Synergistia;Synergistales;Synergistaceae;Pyramidobacter;  Pyramidobacter_piscolens | 0.001 | 0.002 | 0.000 | 0.002 | 0.342 |
| OTU599 | Firmicutes;Clostridia;Clostridiales;Lachnospiraceae | 0.000 | 0.000 | 0.004 | 0.026 | 0.354 |
| OTU6 | ProteoGammaproteoEnterobacteriales;Enterobacteriaceae;Escherichia;  Escherichia | 5.596 | 10.794 | 1.516 | 3.629 | 0.006 |
| OTU60 | Firmicutes;Clostridia;Clostridiales;Clostridiaceae;  Clostridium_sensu_stricto;Clostridium_disporicum | 0.506 | 1.257 | 0.088 | 0.176 | 0.014 |
| OTU600 | Firmicutes;Clostridia;Clostridiales;Ruminococcaceae | 0.000 | 0.000 | 0.000 | 0.001 | 0.180 |
| OTU601 | Bacteroidetes;Bacteroidia;Bacteroidales;Porphyromonadaceae | 0.000 | 0.002 | 0.000 | 0.002 | 0.939 |
| OTU602 | Firmicutes;Clostridia;Clostridiales;Ruminococcaceae;Oscillibacter | 0.000 | 0.000 | 0.000 | 0.001 | 0.180 |
| OTU603 | Firmicutes;Negativicutes;Selenomonadales;Veillonellaceae;Megamonas | 0.001 | 0.002 | 0.000 | 0.000 | 0.069 |
| OTU604 | Firmicutes;Negativicutes;Selenomonadales | 0.001 | 0.003 | 0.000 | 0.000 | 0.310 |
| OTU605 | Firmicutes;Negativicutes;Selenomonadales;Veillonellaceae | 0.000 | 0.000 | 0.000 | 0.001 | 0.354 |
| OTU606 | Firmicutes;Clostridia;Clostridiales;Ruminococcaceae | 0.000 | 0.000 | 0.010 | 0.049 | 0.016 |
| OTU607 | Firmicutes;Negativicutes;Selenomonadales;Veillonellaceae | 0.000 | 0.002 | 0.000 | 0.003 | 0.540 |
| OTU608 | Firmicutes;Clostridia;Clostridiales;Lachnospiraceae;Clostridium_XlVb;  Clostridium_lactatifermentans | 0.001 | 0.006 | 0.000 | 0.000 | 0.310 |
| OTU61 | Bacteroidetes;Bacteroidia;Bacteroidales;Porphyromonadaceae;  Parabacteroides;Parabacteroides_merdae | 0.127 | 0.185 | 0.275 | 0.540 | 0.874 |
| OTU610 | Firmicutes;Clostridia;Clostridiales;Ruminococcaceae | 0.000 | 0.000 | 0.001 | 0.003 | 0.180 |
| OTU611 | Bacteroidetes;Bacteroidia;Bacteroidales | 0.000 | 0.000 | 0.000 | 0.002 | 0.354 |
| OTU612 | Firmicutes;Negativicutes;Selenomonadales;Veillonellaceae;Dialister | 0.002 | 0.010 | 0.001 | 0.007 | 0.869 |
| OTU613 | Bacteroidetes;Bacteroidia;Bacteroidales;Prevotellaceae;Prevotella;  Prevotella_copri | 0.001 | 0.003 | 0.000 | 0.000 | 0.069 |
| OTU614 | ActinoActinoCoriobacteriales;Coriobacteriaceae;Senegalimassilia;  Senegalimassilia_anaerobia | 0.001 | 0.004 | 0.000 | 0.001 | 0.664 |
| OTU615 | ActinoActinoCoriobacteriales;Coriobacteriaceae;Adlercreutzia;  Adlercreutzia_equolifaciens | 0.001 | 0.003 | 0.001 | 0.002 | 0.857 |
| OTU616 | Bacteroidetes;Bacteroidia;Bacteroidales;Prevotellaceae;Prevotella | 0.008 | 0.043 | 0.000 | 0.000 | 0.310 |
| OTU617 | Firmicutes;Clostridia;Clostridiales;Ruminococcaceae;Clostridium_IV;  Clostridium_leptum | 0.000 | 0.000 | 0.000 | 0.002 | 0.354 |
| OTU618 | Firmicutes;Negativicutes;Selenomonadales;Veillonellaceae;Megasphaera;  Megasphaera_indica | 0.000 | 0.000 | 0.007 | 0.021 | 0.055 |
| OTU619 | Firmicutes;Clostridia;Clostridiales | 0.000 | 0.000 | 0.000 | 0.002 | 0.336 |
| OTU62 | ProteoEpsilonproteoCampylobacterales;Campylobacteraceae;  Campylobacter | 0.077 | 0.437 | 0.000 | 0.000 | 0.278 |
| OTU620 | Firmicutes;Clostridia;Clostridiales | 0.000 | 0.000 | 0.000 | 0.001 | 0.354 |
| OTU621 | Firmicutes;Negativicutes;Selenomonadales;Veillonellaceae;Megamonas | 0.000 | 0.000 | 0.000 | 0.001 | 0.983 |
| OTU622 | Firmicutes;Clostridia;Clostridiales;Lachnospiraceae | 0.002 | 0.006 | 0.006 | 0.013 | 0.034 |
| OTU623 | Firmicutes;Negativicutes;Selenomonadales;Veillonellaceae;Megamonas;  Megamonas_rupellensis | 0.010 | 0.023 | 0.005 | 0.013 | 0.533 |
| OTU624 | Firmicutes;Clostridia;Clostridiales;Ruminococcaceae;Ruminococcus;  Ruminococcus_bromii | 0.000 | 0.001 | 0.000 | 0.000 | 0.949 |
| OTU625 | Firmicutes;Bacilli;Lactobacillales;Lactobacillaceae;Lactobacillus;  Lactobacillus_salivarius | 0.000 | 0.000 | 0.002 | 0.004 | 0.016 |
| OTU626 | Firmicutes;Clostridia;Clostridiales;Ruminococcaceae;Clostridium_IV;  Clostridium_methylpentosum | 0.000 | 0.000 | 0.000 | 0.001 | 0.354 |
| OTU627 | Bacteroidetes;Bacteroidia;Bacteroidales;Prevotellaceae;Prevotella;  Prevotella_stercorea | 0.002 | 0.008 | 0.009 | 0.039 | 0.902 |
| OTU628 | Bacteria | 0.000 | 0.000 | 0.000 | 0.002 | 0.354 |
| OTU629 | Firmicutes;Bacilli;Lactobacillales;Lactobacillaceae;Lactobacillus | 0.000 | 0.002 | 0.000 | 0.000 | 0.517 |
| OTU63 | Verrucomicrobia;Verrucomicrobiae;Verrucomicrobiales;  Verrucomicrobiaceae;Akkermansia;Akkermansia_muciniphila | 0.006 | 0.024 | 0.074 | 0.397 | 0.726 |
| OTU630 | Firmicutes;Negativicutes;Selenomonadales;Veillonellaceae;Megamonas;  Megamonas_funiformis | 0.000 | 0.000 | 0.001 | 0.002 | 0.096 |
| OTU631 | Bacteroidetes;Bacteroidia;Bacteroidales;Porphyromonadaceae;Barnesiella;Barnesiella_intestinihominis | 0.000 | 0.000 | 0.000 | 0.002 | 0.354 |
| OTU632 | Bacteroidetes;Bacteroidia;Bacteroidales;Prevotellaceae;Prevotella;  Prevotella_copri | 0.000 | 0.000 | 0.014 | 0.058 | 0.180 |
| OTU633 | Firmicutes;Clostridia;Clostridiales;Ruminococcaceae | 0.000 | 0.000 | 0.001 | 0.005 | 0.599 |
| OTU634 | Firmicutes;Clostridia;Clostridiales;Lachnospiraceae;Blautia;  Blautia_glucerasea | 0.073 | 0.104 | 0.057 | 0.101 | 0.306 |
| OTU635 | Firmicutes;Clostridia;Clostridiales;Ruminococcaceae;Gemmiger;  Gemmiger_formicilis | 0.001 | 0.003 | 0.009 | 0.044 | 0.039 |
| OTU636 | Firmicutes;Clostridia;Clostridiales;Lachnospiraceae;Roseburia | 0.174 | 0.328 | 0.323 | 0.452 | 0.013 |
| OTU637 | FusoFusobacteriia;Fusobacteriales;Fusobacteriaceae;Fusobacterium;  Fusobacterium_mortiferum | 0.001 | 0.003 | 0.002 | 0.013 | 0.111 |
| OTU638 | Bacteroidetes;Bacteroidia;Bacteroidales;Prevotellaceae;Prevotella;  Prevotella_copri | 0.000 | 0.000 | 0.045 | 0.168 | 0.052 |
| OTU639 | Firmicutes;Clostridia;Clostridiales;Lachnospiraceae;  Lachnospiracea_incertae_sedis;Ruminococcus_gnavus | 0.001 | 0.008 | 0.000 | 0.000 | 0.310 |
| OTU64 | Bacteroidetes;Bacteroidia;Bacteroidales;Rikenellaceae;Alistipes;  Alistipes_onderdonkii | 0.012 | 0.031 | 0.170 | 0.654 | 0.069 |
| OTU640 | Firmicutes;Negativicutes;Selenomonadales;Veillonellaceae;Megamonas;  Megamonas_funiformis | 0.016 | 0.058 | 0.013 | 0.028 | 0.684 |
| OTU641 | Firmicutes;Clostridia;Clostridiales;Lachnospiraceae;Clostridium_XlVa | 0.001 | 0.005 | 0.001 | 0.005 | 0.963 |
| OTU642 | Bacteroidetes;Bacteroidia;Bacteroidales | 0.000 | 0.000 | 0.000 | 0.002 | 0.354 |
| OTU643 | Firmicutes;Negativicutes;Selenomonadales;Veillonellaceae | 0.000 | 0.000 | 0.000 | 0.002 | 0.180 |
| OTU644 | Firmicutes;Clostridia;Clostridiales;Lachnospiraceae | 0.005 | 0.011 | 0.015 | 0.039 | 0.661 |
| OTU645 | Firmicutes;Clostridia;Clostridiales | 0.000 | 0.000 | 0.000 | 0.002 | 0.180 |
| OTU646 | Firmicutes;Clostridia;Clostridiales;Ruminococcaceae | 0.000 | 0.001 | 0.000 | 0.001 | 0.555 |
| OTU647 | Firmicutes;Clostridia;Clostridiales;Ruminococcaceae | 0.000 | 0.000 | 0.003 | 0.010 | 0.029 |
| OTU648 | Bacteroidetes;Bacteroidia;Bacteroidales;Prevotellaceae;Prevotella;  Prevotella_copri | 0.004 | 0.009 | 0.001 | 0.003 | 0.813 |
| OTU649 | Bacteroidetes;Bacteroidia;Bacteroidales;Prevotellaceae;Prevotella;  Prevotella_copri | 0.002 | 0.004 | 0.001 | 0.004 | 0.611 |
| OTU65 | Firmicutes;Clostridia;Clostridiales;Lachnospiraceae;Blautia;  Blautia_wexlerae | 0.632 | 0.823 | 0.529 | 0.743 | 0.602 |
| OTU650 | Firmicutes;Clostridia;Clostridiales | 0.000 | 0.001 | 0.000 | 0.003 | 0.939 |
| OTU651 | Firmicutes;Clostridia;Clostridiales;Lachnospiraceae | 0.060 | 0.078 | 0.106 | 0.134 | 0.057 |
| OTU652 | Bacteroidetes;Bacteroidia;Bacteroidales;Prevotellaceae;Prevotella;  Prevotella_copri | 0.004 | 0.012 | 0.002 | 0.004 | 0.754 |
| OTU653 | Bacteroidetes;Bacteroidia;Bacteroidales;Porphyromonadaceae;  Butyricimonas;Butyricimonas_synergistica | 0.000 | 0.001 | 0.000 | 0.001 | 0.983 |
| OTU654 | Firmicutes;Clostridia;Clostridiales;Ruminococcaceae | 0.000 | 0.001 | 0.006 | 0.030 | 0.440 |
| OTU655 | Firmicutes;Clostridia;Clostridiales;Lachnospiraceae;Roseburia | 0.001 | 0.003 | 0.011 | 0.045 | 0.079 |
| OTU656 | ProteoBetaproteoBurkholderiales;Oxalobacteraceae;Undibacterium;  Undibacterium_squillarum | 0.001 | 0.003 | 0.000 | 0.000 | 0.142 |
| OTU657 | ProteoBetaproteoBurkholderiales;Sutterellaceae;Parasutterella;  Parasutterella_excrementihominis | 0.001 | 0.003 | 0.000 | 0.001 | 0.101 |
| OTU658 | Firmicutes;Clostridia;Clostridiales;Eubacteriaceae;Eubacterium;  Eubacterium_callanderi | 0.000 | 0.001 | 0.000 | 0.001 | 0.292 |
| OTU659 | ProteoDeltaproteoDesulfovibrionales;Desulfovibrionaceae;Desulfovibrio;  Desulfovibrio_intestinalis | 0.000 | 0.000 | 0.000 | 0.001 | 0.052 |
| OTU66 | Firmicutes;Clostridia;Clostridiales;Ruminococcaceae | 0.133 | 0.402 | 0.193 | 0.434 | 0.029 |
| OTU660 | Firmicutes;Negativicutes;Selenomonadales;Veillonellaceae;Megamonas;  Megamonas_funiformis | 0.000 | 0.001 | 0.000 | 0.000 | 0.310 |
| OTU661 | Bacteroidetes;Bacteroidia;Bacteroidales;Prevotellaceae;Prevotella;  Prevotella_bergensis | 0.001 | 0.003 | 0.000 | 0.000 | 0.517 |
| OTU662 | ProteoGammaproteoPseudomonadales;Pseudomonadaceae;Rhizobacter;  Rhizobacter_fulvus | 0.001 | 0.003 | 0.000 | 0.000 | 0.310 |
| OTU663 | ProteoBetaproteobacteria | 0.000 | 0.001 | 0.000 | 0.000 | 0.310 |
| OTU664 | Firmicutes;Clostridia;Clostridiales;Lachnospiraceae;Ruminococcus2;  Ruminococcus_faecis | 0.010 | 0.021 | 0.010 | 0.027 | 0.692 |
| OTU665 | ProteoGammaproteoPseudomonadales;Moraxellaceae;Enhydrobacter;  Moraxella_osloensis | 0.000 | 0.001 | 0.000 | 0.000 | 0.310 |
| OTU666 | Firmicutes;Clostridia;Clostridiales;Ruminococcaceae | 0.000 | 0.000 | 0.000 | 0.001 | 0.983 |
| OTU667 | Firmicutes;Clostridia;Clostridiales;Clostridiales;Mogibacterium | 0.001 | 0.003 | 0.000 | 0.000 | 0.310 |
| OTU668 | Firmicutes;Clostridia;Clostridiales;Lachnospiraceae;Clostridium_XlVa;  Clostridium_aldenense | 0.004 | 0.007 | 0.013 | 0.048 | 0.733 |
| OTU669 | Firmicutes;Negativicutes;Selenomonadales;Veillonellaceae;Dialister;  Dialister_succinatiphilus | 0.002 | 0.009 | 0.002 | 0.006 | 0.225 |
| OTU67 | Firmicutes;Bacilli;Lactobacillales;Lactobacillaceae;Lactobacillus;  Lactobacillus_sanfranciscensis | 0.119 | 0.478 | 0.044 | 0.246 | 0.343 |
| OTU670 | Firmicutes;Clostridia;Clostridiales;Ruminococcaceae;Anaerotruncus;  Anaerotruncus_colihominis | 0.000 | 0.001 | 0.000 | 0.001 | 0.731 |
| OTU671 | Firmicutes;Clostridia;Clostridiales;Lachnospiraceae | 0.001 | 0.002 | 0.000 | 0.001 | 0.257 |
| OTU672 | Firmicutes;Clostridia;Clostridiales;Ruminococcaceae;Faecalibacterium;  Faecalibacterium_prausnitzii | 0.595 | 1.671 | 1.154 | 1.490 | 0.014 |
| OTU673 | Firmicutes;Clostridia;Clostridiales;Lachnospiraceae | 0.008 | 0.019 | 0.008 | 0.010 | 0.274 |
| OTU674 | Bacteria | 0.000 | 0.000 | 0.001 | 0.008 | 0.354 |
| OTU675 | Bacteroidetes;Bacteroidia;Bacteroidales;Bacteroidaceae;Bacteroides;  Bacteroides_plebeius | 0.001 | 0.004 | 0.000 | 0.000 | 0.278 |
| OTU676 | FusoFusobacteriia;Fusobacteriales;Fusobacteriaceae;Fusobacterium | 0.000 | 0.000 | 0.000 | 0.002 | 0.354 |
| OTU677 | Firmicutes;Clostridia;Clostridiales;Ruminococcaceae | 0.000 | 0.001 | 0.005 | 0.023 | 0.119 |
| OTU678 | ActinoActinoCoriobacteriales;Coriobacteriaceae;Collinsella | 0.000 | 0.001 | 0.000 | 0.000 | 0.310 |
| OTU679 | Bacteroidetes;Bacteroidia;Bacteroidales;Prevotellaceae;Prevotella | 0.000 | 0.001 | 0.000 | 0.001 | 0.649 |
| OTU68 | Firmicutes;Clostridia;Clostridiales;Lachnospiraceae;  Lachnospiracea_incertae_sedis;Eubacterium_eligens | 0.120 | 0.302 | 0.668 | 1.113 | 0.002 |
| OTU680 | Bacteroidetes;Bacteroidia;Bacteroidales;Prevotellaceae;Alloprevotella;  Alloprevotella_rava | 0.004 | 0.022 | 0.000 | 0.000 | 0.949 |
| OTU681 | Firmicutes;Clostridia;Clostridiales;Clostridiales;Parvimonas;  Parvimonas_micra | 0.000 | 0.002 | 0.000 | 0.002 | 0.486 |
| OTU682 | Firmicutes;Clostridia;Clostridiales;Ruminococcaceae;Gemmiger;  Gemmiger_formicilis | 0.006 | 0.022 | 0.002 | 0.004 | 0.550 |
| OTU683 | Firmicutes;Clostridia;Clostridiales | 0.000 | 0.000 | 0.000 | 0.002 | 0.180 |
| OTU684 | Firmicutes;Negativicutes;Selenomonadales;Acidaminococcaceae;  Phascolarctobacterium;Phascolarctobacterium_succinatutens | 0.000 | 0.001 | 0.000 | 0.001 | 0.367 |
| OTU685 | Bacteroidetes;Bacteroidia;Bacteroidales;Prevotellaceae | 0.003 | 0.019 | 0.001 | 0.003 | 0.384 |
| OTU686 | Bacteroidetes;Bacteroidia;Bacteroidales;Prevotellaceae;Prevotella | 0.001 | 0.005 | 0.000 | 0.000 | 0.310 |
| OTU687 | Firmicutes | 0.000 | 0.000 | 0.001 | 0.005 | 0.983 |
| OTU688 | Bacteroidetes;Bacteroidia;Bacteroidales;Prevotellaceae;Prevotella;  Prevotella_copri | 0.004 | 0.006 | 0.206 | 1.191 | 0.731 |
| OTU689 | ActinoActinoActinomycetales;Corynebacteriaceae;Corynebacterium;  Corynebacterium_durum | 0.000 | 0.001 | 0.000 | 0.000 | 0.317 |
| OTU69 | Bacteroidetes;Bacteroidia;Bacteroidales;Bacteroidaceae;Bacteroides;  Bacteroides_caccae | 0.315 | 0.623 | 0.427 | 0.794 | 0.119 |
| OTU690 | Firmicutes;Clostridia;Clostridiales;Ruminococcaceae;Sporobacter;  Sporobacter_termitidis | 0.005 | 0.017 | 0.019 | 0.041 | 0.046 |
| OTU691 | Firmicutes;Clostridia;Clostridiales | 0.000 | 0.000 | 0.000 | 0.002 | 0.096 |
| OTU692 | Firmicutes;Clostridia;Clostridiales | 0.000 | 0.001 | 0.001 | 0.003 | 0.119 |
| OTU693 | Firmicutes;Negativicutes;Selenomonadales;Veillonellaceae;Dialister;  Dialister_succinatiphilus | 0.009 | 0.053 | 0.000 | 0.000 | 0.310 |
| OTU694 | Bacteroidetes;Bacteroidia;Bacteroidales;Porphyromonadaceae | 0.000 | 0.001 | 0.000 | 0.001 | 0.970 |
| OTU695 | ProteoAlphaproteoRhodospirillales;Acetobacteraceae;Acetobacter;  Acetobacter_pasteurianus | 0.000 | 0.000 | 0.000 | 0.003 | 0.354 |
| OTU696 | Candidatus_SacchariUnclassified;Unclassified;Unclassified;  SacchariTM7_phylum | 0.001 | 0.002 | 0.000 | 0.001 | 0.199 |
| OTU697 | Lentisphaerae;Lentisphaeria;Victivallales;Victivallaceae;Victivallis;  Victivallis_vadensis | 0.000 | 0.000 | 0.000 | 0.002 | 0.354 |
| OTU698 | FusoFusobacteriia;Fusobacteriales;Fusobacteriaceae;Fusobacterium;  Fusobacterium_varium | 0.000 | 0.001 | 0.001 | 0.006 | 0.540 |
| OTU699 | Firmicutes;Clostridia;Clostridiales | 0.000 | 0.000 | 0.000 | 0.001 | 0.096 |
| OTU7 | FusoFusobacteriia;Fusobacteriales;Fusobacteriaceae;Fusobacterium;  Fusobacterium_mortiferum | 2.905 | 8.180 | 0.162 | 0.754 | 0.119 |
| OTU70 | Bacteroidetes;Bacteroidia;Bacteroidales;Prevotellaceae;Prevotella | 0.105 | 0.575 | 0.000 | 0.001 | 0.241 |
| OTU700 | ActinoActinoCoriobacteriales;Coriobacteriaceae | 0.000 | 0.000 | 0.001 | 0.002 | 0.113 |
| OTU701 | Firmicutes;Negativicutes;Selenomonadales;Veillonellaceae;Megamonas | 0.000 | 0.000 | 0.004 | 0.019 | 0.336 |
| OTU702 | Firmicutes;Clostridia;Clostridiales;Lachnospiraceae | 0.005 | 0.010 | 0.014 | 0.026 | 0.382 |
| OTU703 | Bacteroidetes;Bacteroidia;Bacteroidales;Porphyromonadaceae | 0.000 | 0.002 | 0.000 | 0.001 | 0.889 |
| OTU704 | Bacteroidetes;Bacteroidia;Bacteroidales;Prevotellaceae;Prevotella;  Prevotella_copri | 0.000 | 0.001 | 0.006 | 0.033 | 1.000 |
| OTU705 | Firmicutes;Clostridia;Clostridiales;Ruminococcaceae | 0.000 | 0.000 | 0.000 | 0.001 | 0.096 |
| OTU706 | Bacteroidetes;Bacteroidia;Bacteroidales;Bacteroidaceae;Bacteroides | 0.000 | 0.001 | 0.002 | 0.010 | 0.624 |
| OTU707 | Bacteroidetes;Bacteroidia;Bacteroidales;Prevotellaceae;Alloprevotella;  Alloprevotella_rava | 0.001 | 0.007 | 0.000 | 0.001 | 0.949 |
| OTU708 | Firmicutes;Clostridia;Clostridiales;Lachnospiraceae | 0.000 | 0.000 | 0.002 | 0.008 | 0.052 |
| OTU709 | Firmicutes;Clostridia;Clostridiales;Lachnospiraceae;Clostridium_XlVb | 0.000 | 0.000 | 0.000 | 0.001 | 0.354 |
| OTU71 | Firmicutes;Clostridia;Clostridiales;Lachnospiraceae;Lachnospira;  Lachnospira_multipara | 0.195 | 0.872 | 0.304 | 0.653 | 0.024 |
| OTU710 | Firmicutes;Clostridia;Clostridiales;Lachnospiraceae | 0.007 | 0.008 | 0.024 | 0.030 | 0.003 |
| OTU711 | Firmicutes;Negativicutes;Selenomonadales;Veillonellaceae;Megasphaera;  Megasphaera_indica | 0.001 | 0.005 | 0.000 | 0.000 | 0.310 |
| OTU712 | Firmicutes;Bacilli;Lactobacillales;Carnobacteriaceae;Granulicatella;  Granulicatella_elegans | 0.000 | 0.001 | 0.000 | 0.000 | 0.069 |
| OTU713 | Candidatus_SacchariUnclassified;Unclassified;Unclassified;  Saccharibacteria | 0.000 | 0.001 | 0.000 | 0.001 | 0.210 |
| OTU714 | Bacteroidetes;Bacteroidia;Bacteroidales;Bacteroidaceae;Bacteroides;  Bacteroides_coprocola | 0.000 | 0.000 | 0.000 | 0.000 | 0.983 |
| OTU715 | Firmicutes;Clostridia;Clostridiales;Christensenellaceae;Christensenella;  Christensenella_minuta | 0.000 | 0.000 | 0.000 | 0.001 | 0.336 |
| OTU716 | Firmicutes;Clostridia;Clostridiales;Ruminococcaceae | 0.000 | 0.000 | 0.000 | 0.002 | 0.354 |
| OTU717 | Firmicutes;Clostridia;Clostridiales;Lachnospiraceae | 0.006 | 0.012 | 0.011 | 0.034 | 0.264 |
| OTU718 | ProteoBetaproteoBurkholderiales;Sutterellaceae;Parasutterella;  Parasutterella_excrementihominis | 0.000 | 0.001 | 0.000 | 0.002 | 0.988 |
| OTU719 | Firmicutes;Clostridia;Clostridiales;Lachnospiraceae;Roseburia | 0.000 | 0.001 | 0.002 | 0.006 | 0.005 |
| OTU72 | Bacteroidetes;Bacteroidia;Bacteroidales;Rikenellaceae;Alistipes | 0.056 | 0.128 | 0.233 | 0.476 | 0.772 |
| OTU720 | ProteoBetaproteoBurkholderiales;Sutterellaceae;Sutterella;  Sutterella_wadsworthensis | 0.014 | 0.046 | 0.000 | 0.001 | 0.525 |
| OTU721 | Firmicutes;Clostridia;Clostridiales | 0.000 | 0.000 | 0.000 | 0.001 | 0.336 |
| OTU722 | Firmicutes;Clostridia;Clostridiales;Lachnospiraceae | 0.000 | 0.000 | 0.001 | 0.003 | 0.052 |
| OTU723 | Firmicutes;Clostridia;Clostridiales;Lachnospiraceae;Roseburia;  Roseburia_inulinivorans | 0.002 | 0.009 | 0.005 | 0.018 | 0.040 |
| OTU724 | Firmicutes;Bacilli;Lactobacillales;Lactobacillaceae;Lactobacillus;  Lactobacillus_mucosae | 0.000 | 0.001 | 0.000 | 0.000 | 0.141 |
| OTU725 | Firmicutes;Clostridia;Clostridiales;Lachnospiraceae;Eisenbergiella;  Eisenbergiella_tayi | 0.000 | 0.000 | 0.000 | 0.002 | 0.354 |
| OTU726 | Firmicutes;Clostridia;Clostridiales;Lachnospiraceae;Clostridium_XlVa | 0.005 | 0.008 | 0.021 | 0.055 | 0.109 |
| OTU727 | ProteoBetaproteoBurkholderiales;Sutterellaceae;Parasutterella;  Parasutterella_excrementihominis | 0.000 | 0.001 | 0.000 | 0.000 | 0.310 |
| OTU728 | Synergistetes;Synergistia;Synergistales;Synergistaceae;Pyramidobacter;  Pyramidobacter_piscolens | 0.000 | 0.000 | 0.000 | 0.001 | 0.354 |
| OTU729 | Bacteroidetes;Bacteroidia;Bacteroidales;Prevotellaceae;Prevotella;  Prevotella_stercorea | 0.004 | 0.022 | 0.000 | 0.000 | 0.310 |
| OTU73 | Firmicutes;Clostridia;Clostridiales;Lachnospiraceae;Dorea;  Dorea_longicatena | 0.289 | 0.461 | 0.156 | 0.269 | 0.283 |
| OTU730 | Bacteroidetes;Bacteroidia;Bacteroidales;Bacteroidaceae;Bacteroides;  Bacteroides_plebeius | 0.004 | 0.018 | 0.000 | 0.000 | 0.069 |
| OTU731 | Firmicutes;Clostridia;Clostridiales;Ruminococcaceae;Gemmiger;  Gemmiger_formicilis | 0.007 | 0.034 | 0.004 | 0.014 | 0.271 |
| OTU732 | Firmicutes;Bacilli;Lactobacillales;Streptococcaceae;Lactococcus;  Lactococcus_garvieae | 0.000 | 0.002 | 0.000 | 0.001 | 0.601 |
| OTU733 | Candidatus_SacchariUnclassified;Unclassified;Unclassified;  SacchariTM7_phylum | 0.001 | 0.001 | 0.000 | 0.001 | 0.553 |
| OTU734 | Firmicutes;Clostridia;Clostridiales;Lachnospiraceae | 0.003 | 0.010 | 0.014 | 0.047 | 0.175 |
| OTU735 | Bacteroidetes;Bacteroidia;Bacteroidales;Porphyromonadaceae | 0.000 | 0.001 | 0.000 | 0.000 | 0.310 |
| OTU736 | Firmicutes;Clostridia;Clostridiales;Lachnospiraceae;Clostridium_XlVa | 0.108 | 0.231 | 0.048 | 0.200 | 0.185 |
| OTU737 | Firmicutes;Negativicutes;Selenomonadales;Veillonellaceae;Dialister | 0.001 | 0.006 | 0.001 | 0.003 | 0.949 |
| OTU738 | Firmicutes;Clostridia;Clostridiales;Ruminococcaceae;  Hydrogenoanaerobacterium;Hydrogenoanaerobacterium_saccharovorans | 0.000 | 0.001 | 0.000 | 0.001 | 0.470 |
| OTU739 | Bacteroidetes;Bacteroidia;Bacteroidales;Prevotellaceae;Prevotella;  Prevotella_copri | 0.145 | 0.291 | 0.090 | 0.201 | 0.125 |
| OTU74 | Bacteroidetes;Bacteroidia;Bacteroidales;Porphyromonadaceae;  Porphyromonas;Porphyromonas_endodontalis | 0.049 | 0.276 | 0.000 | 0.001 | 0.563 |
| OTU740 | Bacteroidetes;Bacteroidia;Bacteroidales;Bacteroidaceae;Bacteroides;  Bacteroides_plebeius | 0.002 | 0.006 | 0.002 | 0.008 | 0.296 |
| OTU741 | Bacteroidetes;Bacteroidia;Bacteroidales;Prevotellaceae | 0.002 | 0.006 | 0.000 | 0.001 | 0.265 |
| OTU742 | Bacteroidetes;Bacteroidia;Bacteroidales;Prevotellaceae | 0.003 | 0.018 | 0.000 | 0.000 | 0.310 |
| OTU743 | ActinoActinoCoriobacteriales;Coriobacteriaceae;Enterorhabdus;  Enterorhabdus_caecimuris | 0.000 | 0.001 | 0.000 | 0.000 | 0.949 |
| OTU744 | Bacteroidetes;Bacteroidia;Bacteroidales;Bacteroidaceae;Bacteroides | 0.001 | 0.004 | 0.012 | 0.049 | 0.318 |
| OTU745 | Bacteroidetes;Bacteroidia;Bacteroidales;Prevotellaceae;Prevotella;  Prevotella_copri | 0.010 | 0.023 | 0.005 | 0.014 | 0.075 |
| OTU746 | Firmicutes;Negativicutes;Selenomonadales;Veillonellaceae;Megamonas | 0.001 | 0.007 | 0.001 | 0.007 | 0.939 |
| OTU747 | Firmicutes;Clostridia;Clostridiales;Ruminococcaceae;Clostridium_IV;  Clostridium_methylpentosum | 0.000 | 0.000 | 0.002 | 0.004 | 0.055 |
| OTU748 | Firmicutes;Clostridia;Clostridiales;Lachnospiraceae | 0.059 | 0.061 | 0.097 | 0.142 | 0.470 |
| OTU749 | Firmicutes;Clostridia;Clostridiales | 0.000 | 0.000 | 0.000 | 0.001 | 0.096 |
| OTU75 | Firmicutes;Erysipelotrichia;Erysipelotrichales;Erysipelotrichaceae;  Clostridium_XVIII | 0.218 | 0.375 | 0.105 | 0.216 | 0.084 |
| OTU750 | Bacteroidetes;Bacteroidia;Bacteroidales;Prevotellaceae;Prevotella | 0.000 | 0.000 | 0.001 | 0.008 | 0.354 |
| OTU751 | Firmicutes;Negativicutes;Selenomonadales;Acidaminococcaceae;  Phascolarctobacterium;Phascolarctobacterium_faecium | 0.000 | 0.001 | 0.001 | 0.005 | 0.367 |
| OTU752 | Bacteroidetes;Bacteroidia;Bacteroidales;Bacteroidaceae;Bacteroides;  Bacteroides_plebeius | 0.000 | 0.001 | 0.001 | 0.003 | 0.502 |
| OTU753 | Firmicutes;Clostridia;Clostridiales;Lachnospiraceae;Roseburia;  Roseburia_faecis | 0.000 | 0.000 | 0.001 | 0.003 | 0.008 |
| OTU754 | Firmicutes;Erysipelotrichia;Erysipelotrichales;Erysipelotrichaceae | 0.000 | 0.000 | 0.000 | 0.001 | 0.354 |
| OTU755 | Firmicutes;Negativicutes;Selenomonadales;Veillonellaceae | 0.001 | 0.006 | 0.000 | 0.000 | 0.310 |
| OTU756 | Synergistetes;Synergistia;Synergistales;Synergistaceae;Synergistes;  Synergistes_jonesii | 0.000 | 0.000 | 0.000 | 0.001 | 0.354 |
| OTU757 | ActinoActinoActinomycetales;Actinomycetaceae;Actinomyces;  Actinomyces_graevenitzii | 0.001 | 0.002 | 0.000 | 0.001 | 0.861 |
| OTU758 | Firmicutes;Clostridia;Clostridiales;Lachnospiraceae | 0.016 | 0.022 | 0.022 | 0.040 | 0.655 |
| OTU759 | Firmicutes;Negativicutes;Selenomonadales;Veillonellaceae;Megamonas;  Megamonas_funiformis | 0.006 | 0.019 | 0.007 | 0.024 | 0.529 |
| OTU76 | Firmicutes;Clostridia;Clostridiales | 0.000 | 0.001 | 0.071 | 0.349 | 0.669 |
| OTU760 | Bacteria | 0.000 | 0.001 | 0.000 | 0.000 | 0.310 |
| OTU761 | Bacteroidetes;Bacteroidia;Bacteroidales;Bacteroidaceae;Bacteroides;  Bacteroides_caccae | 0.053 | 0.165 | 0.044 | 0.108 | 0.085 |
| OTU762 | Bacteroidetes;Bacteroidia;Bacteroidales;Prevotellaceae;Prevotella;  Prevotella_copri | 0.000 | 0.000 | 0.001 | 0.006 | 0.599 |
| OTU763 | Firmicutes;Clostridia;Clostridiales;Lachnospiraceae | 0.237 | 0.440 | 0.214 | 0.332 | 0.787 |
| OTU764 | Firmicutes;Clostridia;Clostridiales;Ruminococcaceae | 0.000 | 0.000 | 0.000 | 0.001 | 0.354 |
| OTU765 | Firmicutes;Clostridia;Clostridiales;Clostridiales;Mogibacterium | 0.000 | 0.001 | 0.000 | 0.001 | 0.795 |
| OTU766 | Bacteroidetes;Bacteroidia;Bacteroidales;Prevotellaceae | 0.000 | 0.002 | 0.000 | 0.000 | 0.310 |
| OTU767 | Firmicutes;Clostridia;Clostridiales;Ruminococcaceae;Gemmiger;  Gemmiger_formicilis | 0.000 | 0.000 | 0.003 | 0.011 | 0.031 |
| OTU768 | Firmicutes;Negativicutes;Selenomonadales;Veillonellaceae;Allisonella;  Allisonella_histaminiformans | 0.003 | 0.016 | 0.001 | 0.004 | 0.611 |
| OTU769 | Firmicutes;Clostridia;Clostridiales;Ruminococcaceae | 0.002 | 0.010 | 0.001 | 0.002 | 0.689 |
| OTU77 | Firmicutes;Clostridia;Clostridiales;Ruminococcaceae;Ruminococcus;  Ruminococcus_callidus | 0.008 | 0.030 | 0.124 | 0.337 | 0.025 |
| OTU770 | Firmicutes;Clostridia;Clostridiales;Ruminococcaceae | 0.000 | 0.000 | 0.003 | 0.014 | 0.180 |
| OTU771 | Firmicutes;Clostridia;Clostridiales | 0.000 | 0.000 | 0.001 | 0.002 | 0.016 |
| OTU772 | Bacteroidetes;Bacteroidia;Bacteroidales;Rikenellaceae;Alistipes | 0.000 | 0.000 | 0.000 | 0.001 | 0.354 |
| OTU773 | Firmicutes;Clostridia;Clostridiales | 0.000 | 0.000 | 0.000 | 0.001 | 0.354 |
| OTU774 | CyanoCyanoUnclassified | 0.000 | 0.002 | 0.000 | 0.000 | 0.949 |
| OTU775 | Bacteroidetes;Bacteroidia;Bacteroidales;Prevotellaceae;Prevotella;  Prevotella_stercorea | 0.000 | 0.000 | 0.001 | 0.004 | 0.354 |
| OTU776 | Bacteroidetes;Bacteroidia;Bacteroidales;Bacteroidaceae;Bacteroides | 0.003 | 0.007 | 0.002 | 0.010 | 0.024 |
| OTU777 | Firmicutes;Clostridia;Clostridiales;Lachnospiraceae;Clostridium_XlVa | 0.000 | 0.001 | 0.002 | 0.006 | 0.213 |
| OTU778 | Firmicutes;Clostridia;Clostridiales | 0.000 | 0.000 | 0.002 | 0.011 | 0.180 |
| OTU779 | Firmicutes;Clostridia;Clostridiales;Ruminococcaceae | 0.000 | 0.000 | 0.001 | 0.005 | 0.354 |
| OTU78 | Firmicutes;Negativicutes;Selenomonadales;Veillonellaceae;Mitsuokella;  Mitsuokella_jalaludinii | 0.079 | 0.420 | 0.011 | 0.047 | 0.857 |
| OTU780 | Firmicutes;Clostridia;Clostridiales;Lachnospiraceae;Roseburia;  Roseburia_intestinalis | 0.127 | 0.234 | 0.518 | 0.646 | 0.001 |
| OTU781 | Firmicutes;Bacilli;Lactobacillales;Lactobacillaceae;Lactobacillus;  Lactobacillus_pontis | 0.000 | 0.000 | 0.001 | 0.003 | 0.108 |
| OTU782 | Bacteroidetes;Bacteroidia;Bacteroidales;Bacteroidaceae;Bacteroides;  Bacteroides_stercoris | 0.004 | 0.008 | 0.012 | 0.029 | 0.946 |
| OTU783 | Firmicutes;Clostridia;Clostridiales;Eubacteriaceae;Eubacterium | 0.000 | 0.000 | 0.000 | 0.002 | 0.354 |
| OTU784 | Bacteroidetes;Bacteroidia;Bacteroidales;Prevotellaceae;Prevotella | 0.000 | 0.001 | 0.000 | 0.000 | 0.310 |
| OTU785 | Firmicutes;Clostridia;Clostridiales;Ruminococcaceae | 0.000 | 0.000 | 0.001 | 0.006 | 0.052 |
| OTU786 | Bacteroidetes;Bacteroidia;Bacteroidales;Porphyromonadaceae | 0.000 | 0.001 | 0.000 | 0.000 | 0.310 |
| OTU787 | Verrucomicrobia;Opitutae;Puniceicoccales;Puniceicoccaceae | 0.000 | 0.000 | 0.000 | 0.001 | 0.354 |
| OTU788 | Firmicutes;Clostridia;Clostridiales;Lachnospiraceae | 0.012 | 0.034 | 0.008 | 0.011 | 0.677 |
| OTU789 | Firmicutes;Negativicutes;Selenomonadales;Veillonellaceae;Dialister;  Dialister_succinatiphilus | 0.001 | 0.004 | 0.000 | 0.000 | 0.949 |
| OTU79 | Firmicutes;Clostridia;Clostridiales;Ruminococcaceae;Oscillibacter | 0.030 | 0.050 | 0.253 | 0.583 | 0.043 |
| OTU790 | Firmicutes;Clostridia;Clostridiales;Eubacteriaceae;Acetobacterium | 0.000 | 0.000 | 0.000 | 0.001 | 0.354 |
| OTU791 | Deinococcus-Thermus;Deinococci;Deinococcales;Deinococcaceae;  Deinococcus | 0.000 | 0.001 | 0.000 | 0.000 | 0.949 |
| OTU792 | Firmicutes;Negativicutes;Selenomonadales;Veillonellaceae;Megamonas;  Megamonas_funiformis | 0.000 | 0.000 | 0.001 | 0.007 | 0.180 |
| OTU793 | Bacteroidetes;Bacteroidia;Bacteroidales;Prevotellaceae;Prevotella;  Prevotella_stercorea | 0.006 | 0.032 | 0.003 | 0.015 | 0.949 |
| OTU794 | Firmicutes;Negativicutes;Selenomonadales;Veillonellaceae;Dialister;  Dialister_pneumosintes | 0.000 | 0.000 | 0.000 | 0.000 | 0.983 |
| OTU795 | Firmicutes;Clostridia;Clostridiales;Ruminococcaceae | 0.000 | 0.001 | 0.000 | 0.000 | 0.142 |
| OTU796 | Firmicutes;Clostridia;Clostridiales;Peptoniphilaceae;Peptoniphilus;  Peptoniphilus_olsenii | 0.000 | 0.001 | 0.000 | 0.000 | 0.310 |
| OTU797 | Firmicutes;Clostridia;Clostridiales;Lachnospiraceae | 0.000 | 0.000 | 0.004 | 0.021 | 0.004 |
| OTU798 | Bacteria | 0.000 | 0.000 | 0.000 | 0.001 | 0.180 |
| OTU799 | Bacteroidetes;Bacteroidia;Bacteroidales;Prevotellaceae;Alloprevotella;  Alloprevotella_rava | 0.000 | 0.001 | 0.000 | 0.000 | 0.949 |
| OTU8 | Bacteroidetes;Bacteroidia;Bacteroidales;Bacteroidaceae;Bacteroides;  Bacteroides_plebeius | 2.956 | 7.343 | 2.844 | 9.170 | 0.054 |
| OTU80 | Firmicutes;Clostridia;Clostridiales;Lachnospiraceae;Anaerostipes;  Anaerostipes_hadrus | 0.274 | 0.471 | 0.176 | 0.242 | 0.730 |
| OTU800 | Bacteroidetes;Bacteroidia;Bacteroidales;Porphyromonadaceae | 0.000 | 0.000 | 0.000 | 0.001 | 0.354 |
| OTU801 | Firmicutes;Clostridia;Clostridiales;Ruminococcaceae | 0.000 | 0.001 | 0.000 | 0.000 | 0.949 |
| OTU802 | Firmicutes;Clostridia;Clostridiales;Lachnospiraceae;Roseburia;  Roseburia_faecis | 0.000 | 0.000 | 0.001 | 0.008 | 0.096 |
| OTU803 | ActinoActinoCoriobacteriales;Coriobacteriaceae | 0.000 | 0.000 | 0.000 | 0.001 | 0.354 |
| OTU804 | ProteoEpsilonproteoCampylobacterales;Campylobacteraceae;  Campylobacter;Campylobacter_rectus | 0.000 | 0.000 | 0.000 | 0.002 | 0.354 |
| OTU805 | Bacteria | 0.000 | 0.003 | 0.000 | 0.000 | 0.310 |
| OTU806 | ProteoBetaproteoBurkholderiales;Sutterellaceae;Parasutterella;  Parasutterella_excrementihominis | 0.004 | 0.008 | 0.002 | 0.005 | 0.436 |
| OTU81 | Firmicutes;Clostridia;Clostridiales;Ruminococcaceae | 0.007 | 0.017 | 0.109 | 0.362 | 0.149 |
| OTU82 | Firmicutes;Clostridia;Clostridiales;Ruminococcaceae | 0.034 | 0.082 | 0.294 | 0.545 | 0.002 |
| OTU83 | ProteoDeltaproteoDesulfovibrionales;Desulfovibrionaceae;Bilophila;  Bilophila_wadsworthia | 0.088 | 0.267 | 0.051 | 0.069 | 0.718 |
| OTU84 | Bacteroidetes;Bacteroidia;Bacteroidales;Porphyromonadaceae;  Parabacteroides;Parabacteroides_distasonis | 0.217 | 0.348 | 0.280 | 0.522 | 0.730 |
| OTU85 | Firmicutes;Clostridia;Clostridiales;Lachnospiraceae;  Lachnospiracea_incertae_sedis;Eubacterium_ruminantium | 0.013 | 0.049 | 0.239 | 0.475 | 0.130 |
| OTU86 | Firmicutes;Clostridia;Clostridiales;Ruminococcaceae;Acetanaerobacterium;Acetanaerobacterium_elongatum | 0.016 | 0.057 | 0.062 | 0.237 | 0.849 |
| OTU87 | Firmicutes;Clostridia;Clostridiales;Lachnospiraceae;Clostridium_XlVb;  Clostridium_colinum | 0.055 | 0.115 | 0.145 | 0.280 | 0.303 |
| OTU88 | Firmicutes;Negativicutes;Selenomonadales;Veillonellaceae;Allisonella;  Allisonella_histaminiformans | 0.132 | 0.332 | 0.129 | 0.245 | 0.081 |
| OTU89 | Bacteroidetes;Bacteroidia;Bacteroidales;Prevotellaceae;Prevotella | 0.015 | 0.083 | 0.083 | 0.307 | 0.205 |
| OTU9 | ProteoGammaproteoEnterobacteriales;Enterobacteriaceae;Klebsiella;  Klebsiella_pneumoniae | 3.686 | 12.983 | 0.860 | 1.932 | 0.821 |
| OTU90 | Firmicutes;Clostridia;Clostridiales | 0.000 | 0.001 | 0.065 | 0.277 | 0.113 |
| OTU91 | Firmicutes;Clostridia;Clostridiales | 0.000 | 0.002 | 0.034 | 0.173 | 0.029 |
| OTU92 | Bacteria | 0.000 | 0.000 | 0.036 | 0.185 | 0.016 |
| OTU93 | Firmicutes;Clostridia;Clostridiales;Clostridiaceae;Clostridium_sensu_stricto | 0.009 | 0.039 | 0.045 | 0.268 | 0.121 |
| OTU94 | Firmicutes;Clostridia;Clostridiales;Clostridiaceae;  Clostridium_sensu_stricto;Clostridium_tarantellae | 0.038 | 0.202 | 0.013 | 0.050 | 0.274 |
| OTU95 | Firmicutes;Clostridia;Clostridiales;Ruminococcaceae;Butyricicoccus;  Butyricicoccus_pullicaecorum | 0.049 | 0.190 | 0.085 | 0.155 | 0.062 |
| OTU96 | Firmicutes;Clostridia;Clostridiales;Lachnospiraceae | 0.047 | 0.123 | 0.142 | 0.367 | 0.093 |
| OTU97 | ProteoBetaproteoBurkholderiales | 0.000 | 0.002 | 0.083 | 0.465 | 0.096 |
| OTU98 | Firmicutes;Clostridia;Clostridiales;Eubacteriaceae;Eubacterium;  Eubacterium_coprostanoligenes | 0.051 | 0.198 | 0.025 | 0.070 | 0.795 |
| OTU99 | Firmicutes;Clostridia;Clostridiales;Ruminococcaceae;Clostridium_IV;  Clostridium_leptum | 0.001 | 0.005 | 0.034 | 0.171 | 0.045 |
